# The Entomopathogenic Bacterial Endosymbionts *Xenorhabdus* and *Photorhabdus*: Convergent Lifestyles from Divergent Genomes

**DOI:** 10.1371/journal.pone.0027909

**Published:** 2011-11-18

**Authors:** John M. Chaston, Garret Suen, Sarah L. Tucker, Aaron W. Andersen, Archna Bhasin, Edna Bode, Helge B. Bode, Alexander O. Brachmann, Charles E. Cowles, Kimberly N. Cowles, Creg Darby, Limaris de Léon, Kevin Drace, Zijin Du, Alain Givaudan, Erin E. Herbert Tran, Kelsea A. Jewell, Jennifer J. Knack, Karina C. Krasomil-Osterfeld, Ryan Kukor, Anne Lanois, Phil Latreille, Nancy K. Leimgruber, Carolyn M. Lipke, Renyi Liu, Xiaojun Lu, Eric C. Martens, Pradeep R. Marri, Claudine Médigue, Megan L. Menard, Nancy M. Miller, Nydia Morales-Soto, Stacie Norton, Jean-Claude Ogier, Samantha S. Orchard, Dongjin Park, Youngjin Park, Barbara A. Qurollo, Darby Renneckar Sugar, Gregory R. Richards, Zoé Rouy, Brad Slominski, Kathryn Slominski, Holly Snyder, Brian C. Tjaden, Ransome van der Hoeven, Roy D. Welch, Cathy Wheeler, Bosong Xiang, Brad Barbazuk, Sophie Gaudriault, Brad Goodner, Steven C. Slater, Steven Forst, Barry S. Goldman, Heidi Goodrich-Blair

**Affiliations:** 1 Department of Bacteriology, University of Wisconsin-Madison, Madison, Wisconsin, United States of America; 2 Monsanto Company, St. Louis, Missouri, United States of America; 3 Department of Biology, Valdosta State University, Valdosta, Georgia, United States of America; 4 Institut für Molekulare Biowissenschaften, Goethe Universität Frankfurt, Frankfurt am Main, Germany; 5 Department of Cell and Tissue Biology, University of California San Francisco, San Francisco, California, United States of America; 6 Department of Biology, Mercer University, Macon, Georgia, United States of America; 7 Institut National de la Recherche Agronomique-Université de Montpellier II, Montpellier, France; 8 Université Montpellier, Montpellier, France; 9 Department of Ecology and Evolutionary Biology, University of Arizona, Tucson, Arizona, United States of America; 10 Department of Microbiology and Immunology, University of Michigan, Ann Arbor, Michigan, United States of America; 11 Commissariat à l'Energie Atomique, Direction des Sciences du Vivant, Institut de Génomique, Genoscope and CNRS-UMR 8030, Laboratoire d'Analyse Bioinformatique en Génomique et Métabolisme, Evry, France; 12 Department of Biological Sciences, University of Wisconsin-Milwaukee, Milwaukee, Wisconsin, United States of America; 13 Department of Computer Science, Wellesley College, Wellesley, Massachusetts, United States of America; 14 Department of Biology, Syracuse University, Syracuse, New York, United States of America; 15 Department of Biology, Hiram College, Hiram, Ohio, United States of America; 16 Department of Biology, University of Florida, Gainesville, Florida, United States of America; 17 DOE Great Lakes Bioenergy Research Center, Madison, Wisconsin, United States of America; J. Craig Venter Institute, United States of America

## Abstract

Members of the genus *Xenorhabdus* are entomopathogenic bacteria that associate with nematodes. The nematode-bacteria pair infects and kills insects, with both partners contributing to insect pathogenesis and the bacteria providing nutrition to the nematode from available insect-derived nutrients. The nematode provides the bacteria with protection from predators, access to nutrients, and a mechanism of dispersal. Members of the bacterial genus *Photorhabdus* also associate with nematodes to kill insects, and both genera of bacteria provide similar services to their different nematode hosts through unique physiological and metabolic mechanisms. We posited that these differences would be reflected in their respective genomes. To test this, we sequenced to completion the genomes of *Xenorhabdus nematophila* ATCC 19061 and *Xenorhabdus bovienii* SS-2004. As expected, both *Xenorhabdus* genomes encode many anti-insecticidal compounds, commensurate with their entomopathogenic lifestyle. Despite the similarities in lifestyle between *Xenorhabdus* and *Photorhabdus* bacteria, a comparative analysis of the *Xenorhabdus, Photorhabdus luminescens*, and *P. asymbiotica* genomes suggests genomic divergence. These findings indicate that evolutionary changes shaped by symbiotic interactions can follow different routes to achieve similar end points.

## Introduction

Evolutionary biologists have long sought to distinguish the characteristics that define both convergent and divergent evolutionary history. Understanding divergence in microorganisms, such as Eubacteria, is difficult, because our concept of a bacterial species has undergone radical changes with the advent of whole-genome sequencing [1]. However, our ability to sequence and analyze whole-genomes has begun to provide critical insights into evolutionary patterns. For example, a number of approaches have been used to determine how bacterial genomes reflect evolutionary divergence and convergence, including the exploration of phylogenetic relationships based on average amino acid identity [2], shared gene orthology [3], and correlated indel alignments [4]. More recently, clustering analyses of protein domains for sequenced microbes have been used to identify and predict the niches of these organisms [5]. Those organisms with a similar distribution of protein families (Pfams), but different 16S rRNA evolutionary patterns, suggest convergent evolutionary histories, while organisms with similar 16S rRNA sequences, but different niches (both environmental and functional) suggest divergent evolutionary patterns. As genomic, environmental, and functional datasets become more correlated, these distinctions become more apparent [6], [7].

It is now clear that the composition of bacterial genomes is dynamic, and susceptible to many changes through the processes of genome reduction [8], gene duplication and divergence [9], vertical inheritance [10], and horizontal gene transfer [11], all of which occur at the confluence of multiple pressures, including the environment, mutation, and competition. While it is possible in many bacterial genomes to detect the results of these mechanisms, such as genome reduction in endosymbionts, it remains more difficult to characterize the evolutionary path of those organisms that come from similar niches and have similar phylogenetic relationships. Do they represent a single organism, or have they speciated? One example is the comparison between entomopathogenic bacteria in the genera *Xenorhabdus* and *Photorhabdus*. Both types of bacteria are mutualists with nematodes and pathogens of insects. However, genetic and physiological studies reveal that they use functionally different approaches for these roles [12]–[14], suggesting that *Xenorhabdus* and *Photorhabdus* underwent divergent evolution that arrived at convergent lifestyles.

*Xenorhabdus spp.* are motile, Gram-negative enterobacteria that form mutualistic associations with entomopathogenic soil nematodes in the genus *Steinernema* and are pathogenic towards a variety of insects [14]–[19]. In the nematode, *Xenorhabdus spp*. are carried in a specialized region of the intestine, termed the receptacle [20], of the third-stage infective juvenile (IJ) [21]. The IJs live in the soil until they invade the hemocoel of susceptible insect hosts. The bacteria are released in the insect hemocoel, where they overcome the insect's defense systems and produce numerous virulence factors that participate in suppressing insect immunity and killing the host [22]–[32]. The bacteria proliferate to high levels in the insect cadaver and produce diverse antimicrobial compounds that suppress the growth of antagonistic microorganisms [33]–[36]. *Xenorhabdus spp*. also secrete an array of exoenzymes that stimulate macromolecular degradation, the products of which, together with the bacteria themselves, are thought to provide a nutrient base for nematode growth and reproduction [37]–[41]. When nematode numbers become high and nutrients become limiting in the insect cadaver, nematode progeny re-associate with bacteria and differentiate into colonized, non-feeding IJs that emerge into the soil to forage for new hosts [20], [42], [43]. Thus, the tripartite *Xenorhabdus*-nematode-insect interaction represents a model system in which both mutualistic and pathogenic processes can be studied in a single bacterial species [44].

*Photorhabdus* species, like *Xenorhabdus,* are γ-proteobacteria that have evolved a nematode-mutualistic / insect-pathogenic lifestyle. *Photorhabdus* bacteria colonize the intestines of *Heterorhabditis spp.* nematodes, which carry them into susceptible insects that are killed and degraded for nutrients (reviewed in [45], [46]). Despite their similar lifestyles, *Xenorhabdus* and *Photorhabdus* bacteria display differences in the underlying molecular mechanisms that are used to achieve successful host interactions (reviewed in [12]). For example, both *Xenorhabdus* and *Photorhabdus* must be able to survive responses of the insect immune system, such as antimicrobial peptide (AMP) production, but each uses different mechanisms to overcome AMP challenge. For example, *Photorhabdus* uses lipopolysaccharide (LPS) modification to resist the action of the host-derived AMPs [47]–[49], but *X. nematophila* prevents induction of insect AMP expression altogether [50], [51]. In addition, screens have been conducted in both *Xenorhabdus* and *Photorhabdus* to identify mutants defective in colonizing the infective stage juvenile nematode. Thus far, no overlap in genetic determinants required for colonization has been observed between the two genera [52]–[54]. These molecular and genetic differences are underscored by morphological and life-style differences between the two systems. For example, *Xenorhabdus* and *Photorhabdus* bacteria are carried by the infective juvenile stage in different locations: in a unique *Steinernema* spp. intestinal structure called the receptacle, or an extended region of the anterior intestine of *Heterorhabditis* spp., respectively. Further, the transmission of *P. luminescens* to *H. bacteriophora* infective juvenile progeny requires bacterial colonization of maternal rectal glands and hatching of the progeny within the mother (endotokia matricida) [55]. No such rectal gland colonization has been observed in *S. carpocapsae* nematodes, nor is endotokia matricida essential for IJ colonization (Chaston and Goodrich-Blair, unpubl. data).

To better understand the biology of *Xenorhabdus*, we sequenced the complete genomes of *X. nematophila* ATCC 19061 [17] and *X. bovienii* SS-2004 [56], [57]. Comparison of these *Xenorhabdus* genomes to the sequenced genomes of *Photorhabdus luminescens* subsp. *laumondii* TT01 [58] and *P. asymbiotica* ATCC 43949 [59] provides evidence for genomic divergence between these two genera even though they share similar lifestyles. Our analysis of these two *Xenorhabdus* genomes provides insight into the complex lifestyle of these nematode symbionts, is of interest for understanding bacterially mediated insect infections, and is a resource for using these entomopathogens as biocontrol agents of agriculturally-relevant insect pests.

## Results

Both *Xenorhabdus* and *Photorhabdus* employ similar mechanisms to complete their lifecycle. Their ability to associate with entomopathogenic nematodes is a key driver in their evolution and likely shaped their respective genomes. Below, we compare their genomes, and illustrate the differences that reflect their genomic divergence despite convergent lifestyles.

### *Xenorhabdus* genome characteristics

**Figure 1:**
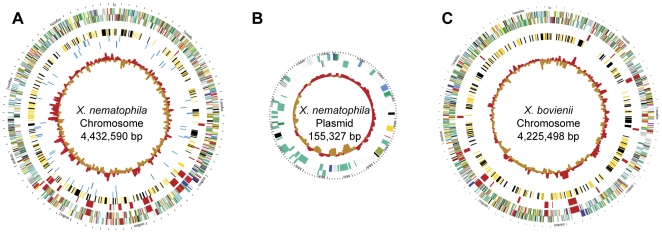
Circular maps of the *Xenorhabdus nematophila* chromosome, its plasmid, and the *Xenorhabdus bovienii* chromosome. Shown are schematic maps of the *X. nematophila* chromosome (A) the *X. nematophila* plasmid (B) and the *X. bovienii* chromosome (C). In all three maps, the outer circle represents scale in base pair coordinates, and moving inward, circles 1 and 2 indicate predicted coding regions transcribed clockwise and counterclockwise respectively. Coding sequences are color coded by their Clusters of Orthologous Groups of proteins (COG) assignments. **Information storage and processing:** green, translation, ribosomal structure and biogenesis; forest green, RNA processing and modification; sea green, transcription; medium aquamarine, replication, recombination and repair; aquamarine, chromatin structure and dynamics; **Cellular processes and signaling:** blue; cell cycle control, cell division, chromosome partitioning; purple, nuclear structure; magenta, defense mechanisms; turquoise, signal transduction mechanisms; sky blue, cell wall/membrane/envelope biogenesis; medium blue, cell motility; royal blue, cytoskeleton; slate blue, extracellular structures; cornflower blue, intracellular trafficking, secretion, and vesicular transport; lavender, posttranslational modification, protein turnover, chaperones; **Metabolism:** red, energy production and conversion; yellow, carbohydrate transport and metabolism; orange, amino acid transport and metabolism; salmon, nucleotide transport and metabolism; pink, coenzyme transport and metabolism; chocolate, lipid transport and metabolism; gold, inorganic ion transport and metabolism; firebrick, secondary metabolites biosynthesis, transport and catabolism; **Poorly characterized:** black, general function prediction only; gray, function unknown. In (A) and (C) circle 3 shows coding regions for non-ribosomal peptide and polyketide synthases, while circle 4 shows genes present in the respective genome, but absent from *Escherichia coli* K12 MG1655; *Photorhabdus luminescens* TTO1; *P. asymbiotica* ATCC 43949 and *Salmonella typhimurium* LT2. For all three maps the innermost circle represents the GC content in 1000-bp windows relative to the mean GC content of the whole sequence.

The *Xenorhabdus nematophila* ATCC 19061 and *Xenorhabdus bovienii* SS-2004 genomes are circular and composed of 4,432,590 and 4,225,498 bp, respectively (Figure 1). The *X. nematophila* genome contains 7 ribosomal RNA operons, encodes 79 tRNA genes, has an average GC content of 44.2%, and is predicted to have 4,299 protein-coding open reading frames (Table 1). *X. nematophila* also contains an extrachromosomal element of 155,327 bp, containing 175 predicted protein-coding open reading frames (Figure 1 and Table 1). The *X. bovienii* genome contains 7 ribosomal RNA operons, encodes for 83 tRNA genes, has an average GC content of 45% and is predicted to contain 4,260 protein coding regions (Figure 1 and Table 1).

**Table 1:** Comparison of the genomic features in Xenorhabdus nematophila ATCC 19061, Xenorhabdus bovienii SS-2004, Photorabdus luminescens TT01, and Photorhabdus asymbiotica ATCC 43949.

Feature	*X. nematophila* ATCC 19061	*X. nematophila plasmid*	*X. bovienii* SS-2004	*P. luminescens* TT01	*P. asymbiotica* ATCC 43949	*P. asymbiotica plasmid*
Size of chromosome (bp)	4,432,590	155,327	4,225,498	5,688,987	5,064,808	29,330
Plasmids	1	-	0	0	1	-
G+C content,%	44.19	45.97	44.97	42.8	42.4	40.5
Coding sequences	4,299	175	4,260	4,683	4,388	27
Function assigned	2,762	42	2,760	1,881	2,678	11
Conserved hypothetical proteins	104	0	99	1,393	787	0
Hypothetical protein	1,433	133	1,401	1,409	1,024	16
% of genome coding	80.52	79.62	85.64	84.00	82.92	79.10
Average length (bp)	860	711	850	969	957	859
Maximal length (bp)	17,985	5,523	28,944	49,104	20,400	4,566
% ATG initiation codons	83.14	61.71	83.73	84.88	81.18	96.29
% GTG initiation codons	7.47	21.14	6.60	7.67	9.43	0
% other initiation codons	9.39	17.15	9.67	7.45	9.39	3.7
RNA elements						
rRNA operons	7	0	7	7	7	0
tRNAs	79	0	83	85	81	0
GenBank Accession	FN667742	FN667743	FN667741	BX470251.1	FM162591.1	FM162592.1

We performed a number of genomic analyses on these two genomes including their metabolism (Text S1), transposases (Text S2), secretion systems (Text S3), small RNAs (Text S4), Tc toxins and hemolysins (Text S5), and secondary metabolites (Text S6). We also performed a detailed proteomic analysis of secreted proteins in *X. nematophila*, which we describe in Text S7, and note that a detailed analysis of regions of genome plasticity was performed previously for these two bacteria [60].

### Unlike their nematode hosts, *Xenorhabdus* and *Photorhabdus* are closely related

*Xenorhabdus* and *Photorhabdus* are more closely related to each other than to any other known species [61]. Members of these genera are known to associate with specific nematode genera and no cross-associations are known. Specifically, *Xenorhabdus* bacteria are found associated with *Steinernema* nematodes whereas *Photorhabdus* bacteria are found associated with *Heterorhabditis* nematodes.

**Figure 2:**
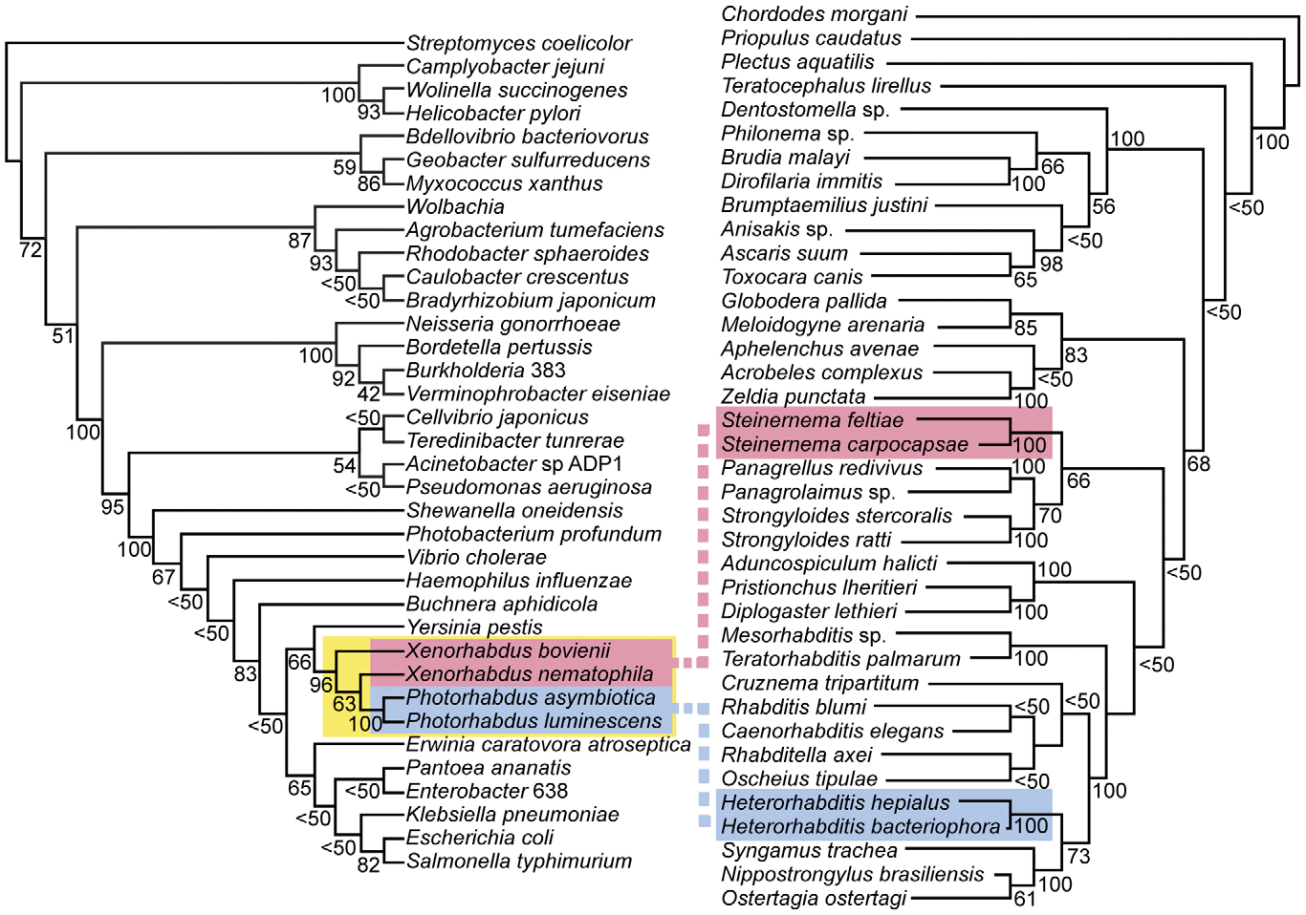
Comparison of the phylogenetic relationships between Enterobacteria and their respective nematode hosts. A 16S rRNA phylogenetic tree for selected bacteria within the phylum Proteobacteria is shown on the left. An 18S inter-ribosomal RNA sequence phylogenetic tree for selected nematodes is shown on the right. The associations of *Xenorhabdus* and *Photorhabdus* bacteria (yellow) with their known hosts are shown with pink and blue lines, respectively. Both phylogenies were constructed using maximum likelihood with bootstrap values indicated at tree nodes (100 replicates).

To confirm the phylogenetic divergence of this association with current data, we constructed two phylogenies for the bacteria and nematodes as shown in Figure 2. We first built a 16S rRNA phylogeny that included both *Xenorhabdus* species in our study and two *Photorhabdus* species, *Photorhabdus luminescens* subsp. *laumondii* TT01 and *P. asymbiotica* ATCC 43949. This tree shows the close phylogenetic relationship between the *Xenorhabdus* and *Photorhabdus* and their placement within the *Enterobacteriaceae*, relative to other bacteria in the Proteobacteria. This 16S rRNA phylogeny was further confirmed by a multi-locus sequence analysis (Text S8). In contrast, a phylogeny based on the 18S inter-ribosomal sequence of nematodes shows that the nematode hosts of *Xenorhabdus* and *Photorhabdus* are not closely related (Figure 2). Specifically, *Xenorhabdus* species are phylogenetically closer to *Photorhabdus* than their respective hosts, *Steinernema* and *Heterorhabditis*, are to each other even though both nematodes belong to the order *Rhabditida* [62].

### A genomic comparison of *Xenorhabdus* and *Photorhabdus*

**Figure 3:**
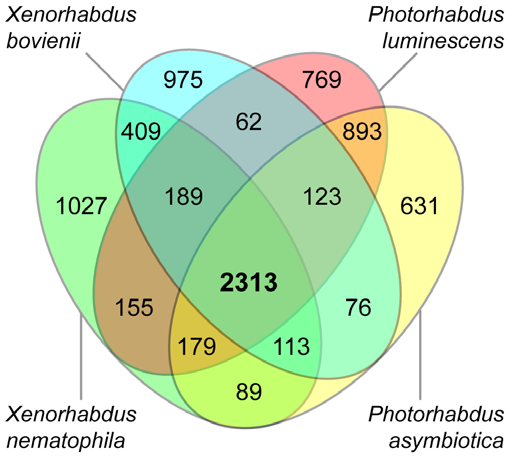
Comparison of the orthologs between sequenced *Xenorhabdus* with *Photorhabdus* bacteria. A Venn diagram showing the number of orthologs between all four genomes.

Despite the relatively close relationship between these *Xenorhabdus/Photorhabdus* lineages (their 16S rRNA genes are over 94% identical), each of these genomes has been disrupted by numerous insertions, deletions, inversions and translocations. An orthology analysis comparing the coding sequences of all four genomes reveals a total of 2,313 shared sequences, with each *Xenorhabdus* genome containing close to 1,000 species-unique genes (Figure 3). Our analysis also reveals that the two *Xenorhabdus* and *Photorhabdus* genomes share more genes exclusive with each other (409 and 893, respectively) than between *Xenorhabdus*-*Photorhabdus* pairs (62 genes for *X. nematophila* and *P. luminescens*; 76 for *X. nematophila* and *P. asymbiotica*; 155 for *X. bovienii* and *P. luminescens*; 170 for *X. bovienii* and *P. asymbiotica*). We also performed a genomic similarity analysis between each pair of genomes using both average nucleotide identity [63] and tetranucleotide frequencies [64] as shown in Figure S1. We found that for all of these similarity metrics, the *Xenorhabdus* genomes are more similar to each other than to the *Photorhabdus* genomes or to other closely related bacteria like *Yersinia pestis* CO92 and *Proteus mirabilis* HI4320. We found the same trend for the *Photorhabdus* genomes, which are more similar to each other than to the *Xenorhabdus* genomes, *Y. pestis*, or *P. mirabilis*.

Further, comparisons of the positions of orthologous genes in these genomes reveals extensive rearrangements in each genome and yields the characteristic X-shaped alignments (data not shown) apparent when inversions encompass and are symmetric to the replication origin [65], [66]. The synteny between the two *Xenorhabdus* genomes is also more highly conserved in the first half of the chromosome; however a large inversion spanning nearly 400 kb has occurred within this region in the *X. bovienii* genome. Although the *Xenorhabdus* genomes harbor large numbers of IS elements, there is no apparent relationship between the number and location of these translocatable elements and the occurrence of genome rearrangements.

### Phylogenomic analysis of X. nematophila, X. bovienii, P. luminescens, and P. asymbiotica

To begin unraveling the metabolic and physiological differences that may exist among these bacterial entomopathogens, we constructed phylogenomic maps for all four *Xenorhabdus* and *Photorhabdus* genomes [67] (Figure S2). Phylogenomics posits that those ORFs sharing a similar evolutionary history will cluster into functional modules corresponding to different aspects of the organism's lifestyle. Construction of a phylogenomic map proceeds by comparing each predicted protein in a genome against a database of predicted proteins from all other completely sequenced genomes. A phylogenetic profile for each protein is thus generated with each cell containing the bit score of the best BLAST hit to a protein in a given microbial genome. These profiles are then clustered to generate a similarity matrix and further visualized as a topographical landscape of mountains where each mountain contains groups of proteins that share phylogenetic history and potentially correspond to putative functional modules (Datasets S1, S2, S3, S4). Overall, we found that all four maps had comparable topography with the *X. nematophila* and *X. bovienii* maps more similar to each other than the *P. luminescens* and *P. asymbiotica* maps (Figure S2).

We then annotated these mountains by performing a gene ontology [68] enrichment analysis to determine if individual mountains contained genes associated with a particular function as shown in Tables S1, S2, S3, S4. In general, we found that the mountains across all four maps reflect the general lifestyle of these bacteria, as mountains enriched for genes associated with transcription and translation; metabolism; energy production and conversion; motility and chemotaxis; and transport were detected. We also found that there were a number of functional modules exclusive to either *Xenorhabdus* or *Photorhabdus* bacteria. Between the *Xenorhabdus* we found mountains over-enriched for genes associated with stress response (GO:0006950) and nuclease activity (GO:0004518). Between the *Photorhabdus*, the most striking over-enriched functional modules are those associated with pathogenesis (GO:0009405), symbiosis, encompassing mutualism through parasitism (GO:0044403), and interspecies interaction between organisms (GO:0044419). An analysis of these two mountains (mountain 35 in *P. luminescens*, Table S3; and mountain 7 in *P. asymbiotica*, Table S4) reveals that they contain a large number of type III secretion system proteins, which are known to be important during insect colonization by the *Photorhabdus*-*Heterorhabditis* pair [69]. Since neither *Xenorhabdus* species is known to contain genes encoding for type III secretion (Text S3), it is not surprising that mountains enriched for this known gene ontology designation do not exist.

### Phylogenomic analysis of conserved *Xenorhabdus* and *Photorhabdus* genes and unique *Xenorhabdus* genes

To gain predictive insights into genetic components that represent divergent and convergent approaches to insect and nematode host-association, we performed an additional phylogenomic clustering analysis of genes specific to either to the genus *Xenorhabdus* alone (class X) or to both *Xenorhabdus* and *Photorhabdus* (class XP). Genes in class XP were generated by retaining only those homologs found between the *Xenorhabdus* and *Photorhabdus* genomes but not in *Salmonella typhimurium* LT2 or *Escherichia coli* K12. We reasoned that *S. typhimurium* LT2 and *E. coli* K12 are reasonable representations of the genetic content within the *Enterobacteriaceae* and by filtering the *Xenorhabdus* and *Photorhabdus* gene sets against these two genomes, we would potentially identify those genes specific to these two genera. A total of 243 genes were identified in this manner, and subsequent phylogenomic mapping analysis revealed a map with 9 mountains (Table S5 and Dataset S5). Similarly, we constructed a phylogenomic map for the 290 orthologs found between *X. bovienii* and *X. nematophila* but not in the *Photorhabdus* genomes, *S. typhimurium* LT2, or *E. coli* K12. This resulted in a phylogenomic map with 15 mountains (Table S6 and Dataset S6). We report our following analysis using *X. nematophila* gene locus names.

One of the strengths of phylogenomic mapping is that every gene on the map is clustered according to a phylogenetic profile that determines in what other bacteria homologs of that gene are present. As a result, additional inferences for a gene can be determined by correlating it to known information about those bacteria that define its phylogenetic profile. We used this approach to analyze the genes on both of these maps by tabulating the known environmental and taxonomic associations of each bacterium that comprises each gene's phylogenetic profile. Given that both *Xenorhabdus* and *Photorhabdus* are host-associated bacteria, we expect that those mountains enriched for genes found in other host-associated bacteria could infer factors necessary for insect or nematode interactions. As a result, we obtained the organismal information provided for each microbe in the complete microbial genome collection in NCBI and used this to categorize each microbe as either host-associated or unknown- / not- host-associated (Dataset S7). A given bacterial species was scored as host-associated if it is found in association with plants, animals, or protozoans as a pathogen, mutualist, or “commensal”.

In general, we found several mountains in each of the X and XP classes that were significantly enriched for genes carried by bacteria that are either host-associated or not host-associated (Table 2) relative to all *X. nematophila* genes. Proteins encoded by the XP class could be necessary for conserved responses to selective pressures encountered in insect hosts or common between *Steinernema* spp. and *Heterorhabditis* spp. host environments. On the other hand, X class proteins are expected to be involved in *Xenorhabdus*-specific responses to *Steinernema* nematode environments and the insects they infect. These proteins could either represent a convergent response to similar host pressures or divergent responses to unique host habitats. We further determined that for most mountains enriched in genes with homologs in host-associated bacteria, those bacteria are significantly over-represented for γ-proteobacteria. This suggests the possibility that these host-association genes might partition by vertical inheritance [10].

**Table 2:** X- and XP-class phylogenomic mountain niche and taxonomy enrichment analysis.

Mount.	No. of Genes	Host-associated vs. not host-associated (*P*-value^a^)	γ-proteobacteria vs. not γ-proteobacteria (*P*-value^b^)	Identified Functional genes
XP1	40	2.81E−08, Over	3.5E−41, Over	Unknown hypothetical proteins
XP2	7	1.17E−07, Over	1.84E−40, Over	Phage genes
XP3	11	6.61E−07, Under	6.66E−07, Under	Transposases
XP4	43	8.07E−24, Over	1.18E−153, Over	TcABC toxins and proteases
XP5	2	-	2.3E−13, Under	2 genes: regulator and peptidoglycan acetylation
XP6	2	1.66E−04, Over	-	2 genes: hypothetical membrane and cytoplasmic proteins
XP7	61	2.18E−23, Over	6.18E−10, Over	Type VI secretion, transport
XP8	17	3.89E−24, Over	3.59E−18, Under	Extracellular metalloprotease precursor
XP9	9	-	1.51E−26, Over	Sodium translocation
XP10	5	-	-	Toxin / antitoxin
XP11	3	-	-	Integrase
XP12	22	-	4.85E−15, Over	Transposase / plasmid
XP13	1	3.43E−04, Under	-	1 gene: AMP-synthetase/ligase
XP14	15	9.14E−69, Under	2.55E−132, Under	Lipopolysaccharide production
XP15	5	5.06E−19, Under	-	Transposase
X1	26	-	1.55E−48, Under	Transposase
X2	7	2.36E−04, Over	1.81E−09, Over	Tellurite resistance
X3	14	4.97E−36, Over	-	Transposase
X4	4	-	-	Transposase
X5	109	-	2.53E−12, Under	“Everything else”
X6	83	-	9.22E−06, Over	Unique *Xenorhabdus* genes
X7	17	-	-	Transposase
X8	14	5.76E−70, Over	7.58E−98, Over	Phage, transposases
X9	16	4.70E−21, Over	2.93E−05. Over	Phage

a *P*-values were calculated using Fisher's Exact Test by comparing all Niche profiles for genes in the mountain against the total number of gene profiles in the *X. nematophila* genome.

b *P*-values were calculated using Fisher's Exact Test by comparing all Taxonomic profiles for genes in the mountain against the total number of gene profiles in the *X. nematophila* genome.

An analysis of the XP class phylogenomic map revealed six mountains that were over-represented for genes from host-associated bacteria (Table 2). These mountains contain genes encoding toxins and proteases (mountains XP1, XP4, XP7, and XP8; Table S5), putative membrane transporters including iron and iron-related acquisition transport systems (XP4, XP7, XP8), transcriptional regulators (XP1, XP4, XP7, and XP8), and toxin/antitoxin members or modules (XP1, XP7, and XP10). Many of these genes are well-known in the *Xenorhabdus* and *Photorhabdus* lifestyle, including the toxins, which are used to kill their respective insects (e.g. Tc Toxin [70], [71] (Text S5), XaxAB Toxin [31], [72], [73], and XhlAB hemolysin [29], [74]. As a result, these shared sets of genes likely represent important factors common between the two genera that may help in stabilizing the nematode-bacteria mutualism in general.

In addition to those mountains enriched for genes from host-associated bacteria, we found other mountains that may also play potential roles in *Xenorhabdus* and *Photorhabdus* interactions with nematodes or insects. For example, mountain XP14 (Table S5) contains members of the Wal lipoolysaccharide (LPS) biosynthesis locus [75], one of which is induced during *X. koppenhoeferi* infection of the white grub *Rhizotrogus majalis* [76] and three other known LPS biosynthesis genes (XNC1_1391, XNC1_2486, and XNC1_2487) that are necessary for both nematode mutualism and pathogenesis in *P. luminescens* [47]. The presence of LPS biosynthesis genes with XP-class genes fits with the current understanding that bacterial LPS plays a key role in both pathogenic and mutualistic associations [77]–[82].

In the X-class phylogenomic map five of the nine mountains contained genes associated with genetic mobility (e.g. transposases) while four did not (Table 2, Table S6). The latter group includes mountain X2, which contains 4 tellurite resistance genes; X5, a relatively large mountain that contains 3 of the known 14 xenocoumacin production genes involved in maintaining cadaver sterility [83]; X6, which contains predominantly genes encoding proteins of unknown function; and X9, which contains 3 groups of 5 phage-encoded genes, each group containing a putative holin protein-encoding gene (identified by manual inspection). Given that this map is specific to only orthologs between the two *Xenorhabdus* genomes, it is likely that the genes clustered within these mountains, such as those hypothetical proteins in mountain X6, are specific to *Xenorhabdus* biology.

## Discussion

The complex association of *Xenorhabdus* and *Photorhabdus* with nematodes is a beautiful example of host-microbe symbioses. In this paper, we report the complete sequencing of the *X. nematophila* ATCC 19061 and *X. bovienii* SS-2004 genomes. Our analysis reveals that *Xenorhabdus* bacteria can produce a large arsenal of insecticidal toxins, commensurate with their known entomopathogenic lifestyle. Our comparative analysis of *Xenorhabdus* and *Photorhabdus* genome provides insight into how their relationships with different nematodes have shaped their evolutionary history.

*Xenorhabdus* and *Photorhabdus* are more phylogenetically similar to each other than their nematode hosts (Figure 2), suggesting that both *Xenorhabdus* and *Photorhabdus* diverged more recently from a common ancestor. This bacterial progenitor may have been capable of colonizing both *Steinernema* and *Heterorhabditis*, and long-term association with their host may have independently given rise to *Xenorhabdus* and *Photorhabdus*. The delineation of these two genera is marked by the fact that each genus can only colonize specific nematode hosts. Importantly, *Xenorhabdus* and *Photorhabdus* are not the only bacteria known to engage in pathogenic symbioses with nematodes. For example, the γ-proteobacterium *Moraxella osloensis* can associate with the nematode *Phasmarhabditis hermaphrodita* and parasitize slugs [84]. *M. osloensis* (family *Pseudomonadaceae*) is phylogenetically distinct from either *Xenorhabdus* or *Photorhabdus* (family *Enterobacteriaceae*). Since *P. hemaphrodita* belongs to the same order as both *Steinernema* and *Heterorhabditis* (Rhabditida), this suggests that γ-proteobacteria have a long association as nematode symbionts. As a result, it is entirely possible that a progenitor of *Xenorhabdus* and *Photorhabdus* differentiated from a more ancient predecessor before associating with their respective nematode hosts. Further divergence would be expected to result in mechanisms that maintain specificity with their respective nematode hosts.

Under this model, these genomes would partition into genus- or species-specific genes that help maintain their specificity and shared homologs that are general to their similar lifestyles. This is supported by our findings that these bacteria share 3,299 orthologs between any *Xenorhabdus*-*Photorhabdus* combination, representing at least 70% of the predicted coding sequences in each genome (Figure 3). As each genus diverged, the number of shared orthologs between genera would be expected to decrease while the number of genera-specific orthologs would increase. This is also supported in our analysis, as we found 2,313 orthologs shared between all four bacteria, representing less than half of the predicted coding sequences in each of their respective genomes. Furthermore, each *Xenorhabdus* and *Photorhabdus* pair share more orthologs exclusive to each other than to any *Xenorhabdus*-*Photorhabdus* pair (Figure 3). These differences are also underscored by our whole-genome average nucleotide identity and tetranucleotide usage analyses, which show that each *Xenorhabdus* and *Photorhabdus* pair is more similar to each other than to any other combination (Figure S1).

The divergence of *Xenorhabdus* and *Photorhabdus* raises two questions: which genes are conserved and which genes have diverged? Our phylogenomic mapping analysis revealed many core physiological pathways are highly conserved across all four bacteria (Table 2 and Tables S1, S2, S3, S4, S5, S6). These include genes encoding toxins and proteases, iron-related transporters, and LPS biosynthesis. Some of these genes, like the toxins, are well known for their interactions with insects, and these toxins have likely been retained across the evolutionary history of these two genera to help maintain the mutualistic relationship they have with their respective nematode hosts (Text S5). Other genes like the LPS biosynthesis cluster are also known for both nematode mutualism and pathogenesis in *P. luminescens* [52], and our finding of homologs in *P. asymbiotica* and the two *Xenorhabdus* bacteria may indicate their similar role in these three bacteria.

In addition to these shared homologs, we also identified putative insect-environment responsive elements in our XP class phylogenomic map. Two of these genes, XNC1_2015 and XNC1_2125 (mountain XP1, Table S5), putatively encode galactophilic lectins and a search of other bacterial genomes revealed homologs in only three other bacteria: *Enterobacter cloacae, Ralstonia solanacearum,* and *Pseudomonas aeruginosa*. One of these, the PA-IL lectin, mediates *P. aeruginosa* adherence to a galactose epitope on the surface of epithelial cells [85], [86] and fibronectin [87]. Similarly, the galactophilic lectin homologs of *Xenorhabdus* and *Photorhabdus* may mediate specific adherence to insect or nematode host tissues. One particularly relevant target is insect blood cells (hemocytes), and indeed, *Drosophila melanogaster* hemocytes express a galactose-containing antigen [88]. Therefore, it is plausible that both *Xenorhabdus* and *Photorhabdus* utilize galactophilic-lectin homologs to adhere to insect hemocytes.

One set of genes revealed in our analysis has likely duplicated and diverged in these two genera. We found that the putative virulence determinants known as invasins have a core set of highly conserved genes found in all four genomes in addition to other invasion genes that are specific to either *Xenorhabdus* or *Photorhabdus* (Text S5). The *Xenorhabdus* invasion proteins are characterized by a domain of unknown function (DUF) domain, whereas the *Photorhabdus* invasins contain Ig-like domains that are related to those found in *E. coli* and *Yersinia*. In *Yersinia*, these proteins are known to play a role in uptake by their hosts, and it is entirely possible that these genes function in a similar manner in *Photorhabdus*. Given that both *Xenorhabdus* and *Photorhabdus* interact with nematode and insect hosts, these genes may play similar roles and their divergence could be linked to the specificity of their known hosts.

Convergent pathways are also present in our analysis. For example, our phylogenomic mapping analysis confirmed previous observations that *Photorhabdus* genomes contain type III secretion system (T3SS) genes that are absent in both *Xenorhabdus* genomes. The T3SS system is necessary for insect colonization by *Photorhabdus*, which uses it to secrete its numerous toxins and insect-killing factors [69], [89]. The presence of this pathway in *Photorhabdus* is likely preserved within the *Enterobacteriaceae*, as many closely related bacterial pathogens like *Yersinia* also use the T3SS to deliver toxins [90]. This would suggest that *Xenorhabdus* lost these genes as it diverged rather than *Photorhabdus* acquiring this system horizontally. In *Xenorhabdus*, delivery of toxins into the insect is not precisely known; however, possible mechanisms include two-partner secretion systems [29] (e.g. XhlAB, Text S5), the flagellar apparatus [23], [91], or outer membrane vesicles [92] (Text S7). As a result, *Xenorhabdus* and *Photorhabdus* have converged upon parallel strategies for toxin delivery using wholly different mechanisms.

We also found differences in the way that *Xenorhabdus* and *Photorhabdus* overcome oxidative stress. Oxidative stress resistance is important for insect pathogenesis [93]–[96] and has been implicated in both *Xenorhabdus* [51], [97] and *Photorhabdus* [98], [99] nematode host interactions. Our phylogenomic analysis of *Xenorhabdus* orthologs revealed a number of genes predicted to confer tellurite resistance (mountain X2, Table S6), a mechanism known to be involved in combating oxidative stress [100]. In contrast, *Photorhabdus* does not have tellurite resistance genes, and may use other mechanisms to respond to reactive oxygen stress, including catalase [96] and luciferase enzymes [101], [102] and the autoinducer-2 pathway [51]. As a result, these two genera may have converged upon ways to overcome oxidative stress using entirely divergent pathways.

A third example of convergence is sterile cadaver maintenance. In addition to selective colonization events that help ensure that the proper symbiont is passed to progeny nematodes (e.g. [55]), both *Xenorhabdus* and *Photorhabdus* produce compounds that prevent other bacteria from thriving within an infected insect cadaver. These products include antibiotics, such as the *Xenorhabdus-*specific xenocoumacins, and a wide variety of other small molecules produced by *Photorhabdus* [103]. Importantly, xenocoumacin is the major antibiotic class produced by *X. nematophila* [83], [104], and genes within the cluster encoding this compound were identified in our *Xenorhabdus*-specific phylogenomic mapping analysis (mountain X5, Table S6). While xenocoumacin production is not known in *X. bovienii*, several biosynthesis gene clusters have also been identified in *X. bovienii* and three orthologs of the *X. nematophila* xenocoumacin-producing genes are present in mountain X5 (*xcnADE*) (though the postulated natural product resulting from the *xcnADE* biosynthesis gene cluster is expected to be structurally dissimilar from xenocoumacin (Bode, unpublished data)). This suggests that *X. nematophila* and *X. bovienii* may use different variations of the same molecular mechanism for antibiotic production. In contrast, *Photorhabdus* do not produce Xenocoumacin class antibiotics but produces the antibiotic isoproylstilbene instead [105] and utilizes bacteriocins called lumicins to prevent other bacteria from thriving within the insect cadaver [106]). Functional assays show that *Xenorhabdus* genomes encode factors that kill closely related *Xenorhabdus* species [107]–[109] but these genes have no sequence similarity to the lumicin-producing genes in *Photorhabdus* with the exception of two *X. bovienii* genes (XBJ1_1085 and XBJ1_1080) that are similar to the *P. luminescens Usp*-like/catalytic domain/typO873-like DNAse/RNase components (plu1894, plu0884, and plu4177). Thus, the mechanisms *Xenorhabdus* and *Photorhabdus* use in secretion mechanisms, response to oxidative stress, and maintenance of a sterile insect cadaver all represent convergent approaches to help maintain their similar lifestyles.

Recently, there has been much discussion among microbiologists regarding what constitutes a bacterial species [110], [111]. Molecular characteristics, such as rRNA sequencing and DNA-DNA hybridization have been used to classify bacteria, but in many cases these are too highly conserved across species to be useful as classification tools. One definition of a species includes the niche of living in another organism. This ecotype model recognizes the special relationship between genes and the environment [112]–[114] and has been proposed to explain bacterial species evolution. One of the basic tenets of this model is that a common bacterial ancestry will be retained among bacterial populations residing within ecological niches. Our analysis of the *Xenorhabdus* and *Photorhabdus* genomes here appear to support this ecotype model of speciation. Selective pressures induced by bacterial-nematode interactions would result in periodic selection [112], [114], which is expected to give rise to genomic changes that ensure the specificity, stability, and maintenance of this symbiosis. Clearly, the bacterial-nematode lifestyle is successful, given its continued existence and expansion in other pairings that parasitize other organisms like slugs [84]. Our findings support the hypothesis that *Xenorhabdus* and *Photorhabdus* diverged from a common ancestor, and, due to the selective pressures of maintaining this symbiosis, evolved different mechanisms to converge upon the same lifestyle.

## Materials and Methods

### Strains

*X. nematophila* ATCC 19061 used in this study was acquired from American Type Culture Collection. The *X. bovienii* strain used in this study was deposited on Jun. 28, 2000 with the Agriculture Research Culture Collection (NRRL) International Depository Authority at 1815 North University Street, in Peoria, Ill. 61604 U.S.A., according to the Budapest Treaty on the International Recognition of the Deposit of Microorganisms for the Purpose of Patent Procedures and was designated as NRRL-30311.

### Genomic DNA extraction

Cultures of *X. nematophila* ATCC 19061 and *X. bovienii* SS-2004 were grown on LB agar supplemented with ampicillin (150 ug/ml). Cells were scraped into 10 ml of LB broth and 6 mL were subcultured into 500 mL LB for 18.5 hours at 30°C. Four 35-ml aliquots were treated in the following manner. Cells were pelleted and resuspended in 15 mL TE buffer (10 mM tris, pH 8.0; 1 mM EDTA, pH 8.0) prior to adding 4 mg proteinase K and 0.66% final concentration of SDS. The solution was incubated at 48–55°C for 2 h prior to extracting twice with 1∶1 phenol-chloroform solution and twice more in chloroform to remove residual phenol. The samples were precipitated by addition of 0.1 volumes of 3 M sodium acetate and 0.6 volumes of isopropanol, and then frozen overnight at −80°C. Samples were then spun for 10 min at 10,000 RPM, washed in cold 70% ethanol and air dried. All four aliquots were resuspended in 15 mL of TE at 4°C overnight, and gently pipetted to aid in resuspension. RNase was added to a final concentration of 25 µg /mL and the samples were incubated at 37°C for 1 h. Samples were then extracted once in one volume of phenol, once in one volume of 1∶1 phenol-chloroform, twice in equal volumes of chloroform, and precipitated with 0.1 volumes of sodium acetate and 2 volumes of 95% ethanol. Pellets were washed in 40 mL of 70% ethanol, dried completely, and re-suspended in 5 ml TE buffer at 4 C overnight and gentle pipetting to a final concentration of 0.64 mg/ml with an OD260/OD280 ratio of 1.9. A single high molecular weight band was visible by gel electrophoresis.

### Genome Sequencing

A total of 90,000 reads were generated for each genome. These reads came from two DNA libraries (insert sizes 2–4 kbp and 4–8 kbp) prepared using mechanical shearing of DNA and cloning into pUC18, followed by a shotgun sequencing approach. The genome was then assembled and edited using the Phred/Phrap/Consed software package [115]–[117]. Finishing was completed by generating an optical map, as previously described [118], cut with the restriction enzymes AflIII and EagI and aligning the assembled sequences to the map. Gaps were closed by sequencing specific products. All rRNA operons were amplified with specific flanking primers, sequenced and assembled individually. All positions with Phred scores less than 40 were re-sequenced using an independent PCR fragment as template. The error rate is estimated to be less than 1∶10,000 bp.

### Genome annotation

Genome annotation was performed using the MaGe annotation pipeline as previously described [119], and all subsequent genomic analyses presented in this study were performed using this annotation. The sequence and annotation for the *X. nematophila* chromosome, plasmid, and *X. bovienii* chromosome are deposited in GenBank under accession numbers FN667742, FN667743, and FN667741, respectively.

### Phylogenetic analysis of bacteria and nematode lineages

A representative phylogeny of the phylum Proteobacteria was constructed using the 16S rRNA sequences obtained from completely sequenced genomes as shown in Figure 2. The 16S rRNA sequence from the complete genome sequence of the Actinobacteria *Streptomyces coelicolor* A3(2) was used as an outgroup. Particular focus on members of the γ-proteobacteria, especially those *Enterobacteriaceae* known to be closely related to *Xenorhabdus* and *Photorhabdus* bacteria, were included. All 16S rRNA sequences were first aligned using MUSCLE [120] and the resulting alignment analyzed using the phylogenetic analysis program phylip [121]. A maximum likelihood tree was generated using this approach and bootstraps were also calculated (100 replicates).

A representative phylogeny of nematodes was also constructed in a similar manner. All 18S rRNA sequences for nematodes used in a previous study by Blaxter *et al*. [122] were obtained and aligned using MUSCLE [120]. The resulting alignment was also analyzed using phylip, and a maximum likelihood tree was generated with bootstraps (100 replicates).

### Ortholog analysis

An orthology analysis was performed for *X. bovienii*, *X. nematophila*, *Photorhabdus asymbiotica*, and *P. luminescens* as shown in Figure 3. This analysis was performed using the Phyloprofile Exploration tool in the MicroScope Microbial Genome Annotation Platform [123]. All four genomes were compared against each other using a minLrap ≥0.6 and identity ≥30% and these data were used to determine all possible ortholog combinations as shown in Figure 3.

### Genomic identity analysis

To determine the similarity of all four genomes at the species level, we calculated three different metrics including average nucleotide identity using BLAST (ANIb) [63], average nucleotide identity using MUMmer (ANIm) [124], and tetranucleotide frequencies [64] using the program JSpecies [125]. All analyses were performed using standard parameters as shown in Figure S1.

### Phylogenomic map construction and gene ontology analysis

Phylogenomic maps were constructed for *X. bovienii*, *X. nematophila*, *Photorhabdus asymbiotica*, and *P. luminescens* as previously described [67]. Briefly, BLASTP [126] was used to align each predicted protein in each genome against a local database of predicted proteins from 1,173 sequenced bacteria obtained from the National Center for Biotechnology Information (NCBI) (http://www.ncbi.nlm.nih.gov/genomes/lproks.cgi, accessed: 09/10/2010). We retained results for each protein match that registered a bit scores >50 and an e-value <1×e-5 in 5 or more of the sequenced genomes. A raw data matrix of bit scores was constructed with each row representing a protein and each column corresponding to a different sequenced genome. Correlations for each pair of proteins were calculated using Spearman's rank correlation. For each protein, the top 50 correlates that had the highest positive correlation scores were retained. Each protein was then assigned an (x, y) coordinate in the plane using a combination of force-directed placement and multi-dimensional scaling. These proteins were then visualized as a three-dimensional topographical map using the computer program VxInsight [127] (Figure S2 and Dataset S1, S2, S3, S4). Each mountain on the map represents those proteins that share similar phylogenetic history, and the height of each mountain is proportional to the density of the proteins within that area. Mountains were determined for each map and gene ontology (GO) [68] assignments were generated for each protein in each mountain using Interpro [128]. GO database files were constructed for each genome, and used to determine the enrichment of GO terms for each mountain using the GO::TermFinder [28] software package. The top three enriched GO terms for each mountain on each map were retained and shown in Tables S1–S4.

A phylogenomic map was also constructed using only those proteins orthologous between the *Xenorhabdus* genomes but not in the *Photorhabdus* genomes, *Salmonella typhimurium* LT2, and *Escherichia coli* K12 using the same method described above. Orthologs specific to the *Xenorhabdus* were determined using the Phyloprofile Exploration tool in the MicroScope Microbial Genome Annotation Platform [123] with an minLrap ≥0.6 and identity ≥30%. A second ortholog-specific phylogenomic map was also constructed using only those proteins orthologous within the *Xenorhabdus* and *Photorhabdus* genomes but not in *Salmonella typhimurium* LT2, and *Escherichia coli* K12. In all cases, the *X. nematophila* proteins corresponding to orthologs were used to construct each respective phylogenomic map (Table S5–S6 and Datasets S5–S6).

Host-association analyses for each mountain on these two phylogenomic maps were performed by identifying the set of microbes for which each protein in the mountain had a significant BLAST hit. The host-association information for each microbe was then determined using the Organism Information data found associated with the complete microbial genome collection in the NCBI (http://www.ncbi.nlm.nih.gov/genomes/lproks.cgi, accessed: 10/25/2010), the Integrated Microbial Genomes Systems (http://img.jgi.doe.gov/cgi-bin/w/main.cgi, accessed: 02/22/2011), and ExPasy (http://au.expasy.org/sprot/hamap/interactions.html, accessed: 02/22/2011). These were further supplemented with our own annotations for those organisms with no host-association entries (Dataset S7). Each species was labeled as “host-associated” or “not host-associated” based on if it was normally found in association with a plant, animal, or protozoan host, as any of a pathogen, mutualist, or “commensal”. Group data were taken directly from NCBI (same as previous). For each mountain, the category for each organism that carried the corresponding protein in the mountain was tabulated. These data were then compared to the same data generated for the entire *X. nematophila* genome using Fisher's Exact Test to determine if proteins within any given mountain were either over- or under-represented for a particular host-association (Table 2). A similar approach was used to determine the over- and under-representation of proteins within each mountain according to taxonomic identity as shown in Table 2.

## Supporting Information

Figure S1 **Whole-genome comparisons of** ***Xenorhabdus, Photorhabdus*** **, and other Enterics.** Analyses were: average nucleotide identity BLAST (A), average nucleotide identity MUMmer (B), and tetranucleotide usage (C). For each analysis, pair-wise similarity scores are shown as calculated using Jspecies [125]. Pair-wise comparisons for *Xenorhabdus* species and *Photorhabdus* species are highlighted in magenta and cyan, respectively. Abbreviations as follows: *Yersinia pestis* CO92 (Ypes), *Proteus mirabilis* HI4320 (Pmir), *Xenorhabdus nematophila* (Xnem), *X. bovienii* (Xbov), *Photorhabdus luminescens* (Plum), and *P. asymbiotica* (Pasy).
(TIF)

Figure S2 **Phylogenomic analysis of** ***Xenorhabdus*** **and** ***Photorhabdus*** **species**. *Xenorhabdus nematophila* (A) and *X. bovienii* (B) maps have a more similar topography to each other than to the *Photorhabdus luminescens* (C) and *P. asymbiotica* (D) maps.
(TIF)

Table S1 **Statistical enrichment of functional groups for each mountain on the** ***Xenorhabdus nematophila*** **phylogenomic map.**
(DOC)

Table S2 **Statistical enrichment of functional groups for each mountain on the** ***Xenorhabdus bovienii*** **phylogenomic map.**
(DOC)

Table S3 **Statistical enrichment of functional groups for each mountain on the** ***Photorhabdus luminescens*** **phylogenomic map.**
(DOC)

Table S4 **Statistical enrichment of functional groups for each mountain on the** ***Photorhabdus asymbiotica*** **phylogenomic map.**
(DOC)

Table S5 **Gene identities and annotations found within mountains on a phylogenomic map constructed for orthologous genes found between the** ***Xenorhabdus*** **and** ***Photorhabdus*** **genomes but not in** ***Salmonella typhimurium*** **LT2, or** ***Escherichia coli*** **K12.**
(DOC)

Table S6 **Gene identities and annotations found within mountains on a phylogenomic map constructed for orthologous genes found between** ***Xenorhabdus nematophila*** **and** ***X. bovienii*** **but not in** ***Photorhabdus luminescens*** **,** ***P. asymbiotica*** **, Salmonella** ***typhimurium*** **LT2, or** ***Escherichia coli*** **K12.**
(DOC)

Text S1 **General Metabolism.**
(DOC)

Text S2 **Transposases.**
(DOC)

Text S3 **Secretion Systems.**
(DOC)

Text S4 **Small RNAs.**
(DOC)

Text S5 **Toxins, Cytotoxins, and Invasins.**
(DOC)

Text S6 **Secondary Metabolites.**
(DOC)

Text S7 A proteomic analysis of the *Xenorhabdus nematophila* supernatant.
(DOC)

Text S8 *Xenorhabdus* multi-locus sequence analysis.
(DOC)

Dataset S1 A phylogenomic map for *Xenorhabdus nematophila* viewable with the provided computer program VxInsight.
(RAR)

Dataset S2 A phylogenomic map for *Xenorhabdus bovienii* viewable with the provided computer program VxInsight.
(RAR)

Dataset S3 A phylogenomic map for *Photorhabdus luminescens* viewable with the provided computer program VxInsight.
(RAR)

Dataset S4 A phylogenomic map for *Photorhabdus asymbiotica* viewable with the provided computer program VxInsight.
(RAR)

Dataset S5 A phylogenomic map for *Xenorhabdus*- and *Photorhabdus*-specific homologs viewable with the provided computer program VxInsight.
(RAR)

Dataset S6 A phylogenomic map for *Xenorhabdus-*specific homologs viewable with the provided computer program VxInsight.
(RAR)

Dataset S7 Spreadsheet containing host- or non-host-association designations for all sequenced genomes used to construct the phylogenomic maps in Dataset S5 and S6.
(XLS)
