# Supplementary figures and images for: The Entomopathogenic Bacterial Endosymbionts Xenorhabdus and Photorhabdus: Convergent Lifestyles from Divergent Genomes
Source: PLoS One. 2011 Nov 18;6(11):e27909. doi: 10.1371/journal.pone.0027909 (PMC3220699; doi:10.1371/journal.pone.0027909)

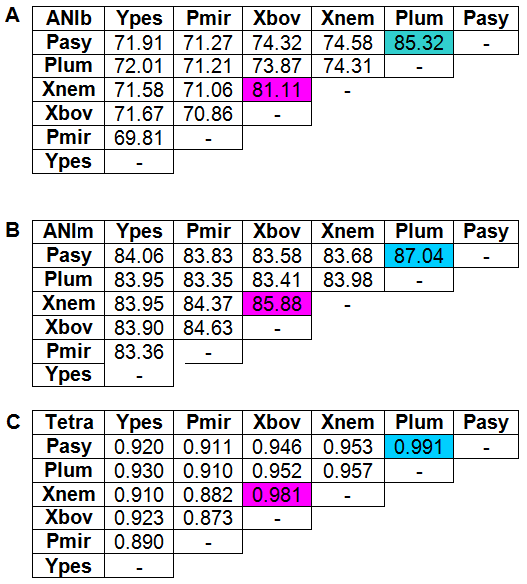

Supplement: Figure S1 — Whole-genome comparisons of Xenorhabdus, Photorhabdus , and other Enterics. Analyses were: average nucleotide identity BLAST (A), average nucleotide identity MUMmer (B), and tetranucleotide usage (C). For each analysis, pair-wise similarity scores are shown as calculated using Jspecies [125]. Pair-wise comparisons for Xenorhabdus species and Photorhabdus species are highlighted in magenta and cyan, respectively. Abbreviations as follows: Yersinia pestis CO92 (Ypes), Proteus mirabilis HI4320 (Pmir), Xenorhabdus nematophila (Xnem), X. bovienii (Xbov), Photorhabdus luminescens (Plum), and P. asymbiotica (Pasy). (TIF) [file pone.0027909.s001.tif]

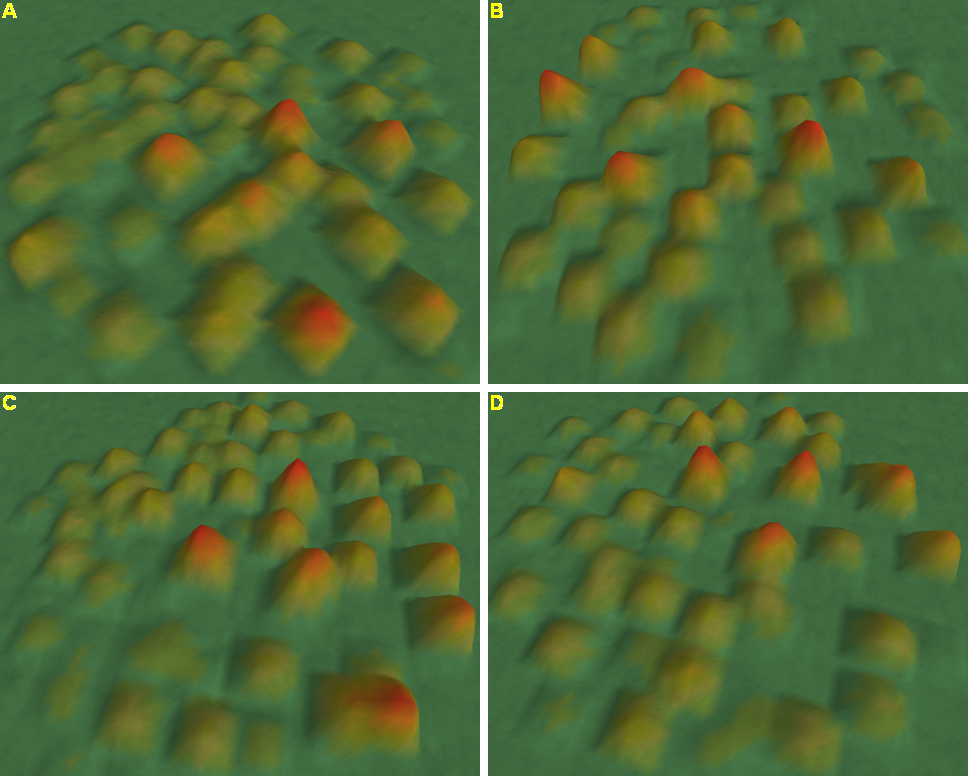

Supplement: Figure S2 — Phylogenomic analysis of Xenorhabdus and Photorhabdus species. Xenorhabdus nematophila (A) and X. bovienii (B) maps have a more similar topography to each other than to the Photorhabdus luminescens (C) and P. asymbiotica (D) maps. (TIF) [file pone.0027909.s002.tif]
