# Supplementary material for: The Entomopathogenic Bacterial Endosymbionts Xenorhabdus and Photorhabdus: Convergent Lifestyles from Divergent Genomes
Source: PLoS One. 2011 Nov 18;6(11):e27909. doi: 10.1371/journal.pone.0027909 (PMC3220699; doi:10.1371/journal.pone.0027909)
Supplement: Table S1 — Statistical enrichment of functional groups for each mountain on the Xenorhabdus nematophila phylogenomic map. (DOC) [file pone.0027909.s003.doc]

**Table S1**. Statistical enrichment of functional groups for each mountain on the *Xenorhabdus nematophila* phylogenomic map.

| **Mount** | **No. of Proteins** | **GOID** | **Term** | ***P*-value** |
| --- | --- | --- | --- | --- |
| 1 | 5 | GO:0006304 | DNA modification | 2.03E-11 |
| 1 | 5 | GO:0043412 | biopolymer modification | 2.11E-08 |
| 1 | 5 | GO:0009007 | site-specific DNA-methyltransferase (adenine-specific) activity | 1.10E-04 |
| 2 | 7 | GO:0015074 | DNA integration | 7.95E-05 |
| 2 | 7 | GO:0006259 | DNA metabolic process | 7.65E-03 |
| 2 | 7 | GO:0003676 | nucleic acid binding | 1.08E-02 |
| 3 | 8 | GO:0004803 | transposase activity | 1.71E-10 |
| 3 | 8 | GO:0006313 | transposition, DNA-mediated | 6.09E-10 |
| 3 | 8 | GO:0006310 | DNA recombination | 5.57E-09 |
| 4 | 9 | GO:0008080 | N-acetyltransferase activity | 6.65E-03 |
| 4 | 9 | GO:0016407 | acetyltransferase activity | 9.62E-03 |
| 4 | 9 | GO:0016747 | transferase activity, transferring acyl groups other than amino-acyl groups | 3.47E-02 |
| 5 | 13 | - | - | - |
| 6 | 14 | GO:0004519 | endonuclease activity | 1.54E-06 |
| 6 | 14 | GO:0004518 | nuclease activity | 1.45E-05 |
| 6 | 14 | GO:0016788 | hydrolase activity, acting on ester bonds | 1.42E-04 |
| 7 | 15 | GO:0015074 | DNA integration | 6.58E-11 |
| 7 | 15 | GO:0006259 | DNA metabolic process | 3.45E-08 |
| 7 | 15 | GO:0003676 | nucleic acid binding | 8.96E-07 |
| 8 | 16 | GO:0004803 | transposase activity | 4.16E-21 |
| 8 | 16 | GO:0006313 | transposition, DNA-mediated | 1.91E-20 |
| 8 | 16 | GO:0006310 | DNA recombination | 1.75E-18 |
| 9 | 17 | GO:0015074 | DNA integration | 1.22E-29 |
| 9 | 17 | GO:0006259 | DNA metabolic process | 7.04E-18 |
| 9 | 17 | GO:0003676 | nucleic acid binding | 3.72E-14 |
| 10 | 20 | - | - | - |
| 11 | 22 | GO:0006310 | DNA recombination | 1.36E-27 |
| 11 | 22 | GO:0000150 | recombinase activity | 3.00E-23 |
| 11 | 22 | GO:0003677 | DNA binding | 3.52E-19 |
| 12 | 23 | - | - | - |
| 13 | 25 | GO:0006950 | response to stress | 1.31E-03 |
| 13 | 25 | GO:0050896 | response to stimulus | 4.51E-03 |
| 14 | 28 | GO:0005515 | protein binding | 1.08E-29 |
| 14 | 28 | GO:0015074 | DNA integration | 7.35E-12 |
| 14 | 28 | GO:0006259 | DNA metabolic process | 2.23E-05 |
| 15 | 30 | GO:0043412 | biopolymer modification | 1.42E-02 |
| 15 | 30 | GO:0003824 | catalytic activity | 4.09E-02 |
| 16 | 31 | GO:0004803 | transposase activity | 3.00E-38 |
| 16 | 31 | GO:0006313 | transposition, DNA-mediated | 2.19E-37 |
| 16 | 31 | GO:0006310 | DNA recombination | 1.32E-33 |
| 17 | 32 | - | - | - |
| 18 | 32 | - | - | - |
| 19 | 34 | GO:0005515 | protein binding | 8.65E-05 |
| 20 | 35 | GO:0004803 | transposase activity | 9.64E-28 |
| 20 | 35 | GO:0006313 | transposition, DNA-mediated | 5.13E-27 |
| 20 | 35 | GO:0006310 | DNA recombination | 4.97E-24 |
| 21 | 39 | GO:0040011 | locomotion | 7.66E-38 |
| 21 | 39 | GO:0019861 | flagellum | 4.85E-34 |
| 21 | 39 | GO:0009288 | flagellin-based flagellum | 2.03E-29 |
| 21 | 39 | GO:0051674 | localization of cell | 6.79E-29 |
| 22 | 53 | GO:0010927 | cellular component assembly involved in morphogenesis | 2.13E-12 |
| 22 | 53 | GO:0022415 | viral reproductive process | 2.13E-12 |
| 22 | 53 | GO:0048646 | anatomical structure formation involved in morphogenesis | 2.13E-12 |
| 23 | 60 | GO:0010124 | phenylacetate catabolic process | 1.46E-08 |
| 23 | 60 | GO:0019439 | aromatic compound catabolic process | 1.46E-08 |
| 23 | 60 | GO:0042178 | xenobiotic catabolic process | 1.46E-08 |
| 24 | 63 | GO:0006259 | DNA metabolic process | 1.14E-26 |
| 24 | 63 | GO:0003676 | nucleic acid binding | 1.97E-20 |
| 24 | 63 | GO:0006139 | nucleobase, nucleoside, nucleotide and nucleic acid metabolic process | 6.03E-18 |
| 25 | 71 | - | - | - |
| 26 | 77 | GO:0008643 | carbohydrate transport | 2.22E-10 |
| 26 | 77 | GO:0051119 | sugar transmembrane transporter activity | 4.72E-10 |
| 26 | 77 | GO:0015144 | carbohydrate transmembrane transporter activity | 9.99E-10 |
| 27 | 78 | GO:0003676 | nucleic acid binding | 2.40E-11 |
| 27 | 78 | GO:0003677 | DNA binding | 5.78E-11 |
| 27 | 78 | GO:0043565 | sequence-specific DNA binding | 9.08E-07 |
| 28 | 86 | GO:0003677 | DNA binding | 1.43E-05 |
| 28 | 86 | GO:0003676 | nucleic acid binding | 6.80E-05 |
| 28 | 86 | GO:0015074 | DNA integration | 4.84E-03 |
| 29 | 128 | GO:0000036 | acyl carrier activity | 6.02E-58 |
| 29 | 128 | GO:0031177 | phosphopantetheine binding | 3.79E-41 |
| 29 | 128 | GO:0016597 | amino acid binding | 5.87E-33 |
| 30 | 136 | GO:0005215 | transporter activity | 6.00E-45 |
| 30 | 136 | GO:0006810 | transport | 8.79E-33 |
| 30 | 136 | GO:0051234 | establishment of localization | 8.79E-33 |
| 31 | 151 | GO:0009987 | cellular process | 2.36E-29 |
| 31 | 151 | GO:0044237 | cellular metabolic process | 1.02E-28 |
| 31 | 151 | GO:0044260 | cellular macromolecule metabolic process | 1.40E-23 |
| 32 | 178 | GO:0009058 | biosynthetic process | 5.65E-35 |
| 32 | 178 | GO:0003824 | catalytic activity | 6.23E-31 |
| 32 | 178 | GO:0044249 | cellular biosynthetic process | 3.67E-27 |
| 33 | 182 | GO:0008152 | metabolic process | 4.21E-07 |
| 33 | 182 | GO:0009058 | biosynthetic process | 3.73E-05 |
| 33 | 182 | GO:0005975 | carbohydrate metabolic process | 7.15E-05 |
| 34 | 225 | GO:0016020 | membrane | 4.02E-14 |
| 34 | 225 | GO:0006810 | transport | 1.01E-08 |
| 34 | 225 | GO:0051234 | establishment of localization | 1.01E-08 |
| 35 | 290 | GO:0016491 | oxidoreductase activity | 1.22E-26 |
| 35 | 290 | GO:0055114 | oxidation reduction | 1.69E-20 |
| 35 | 290 | GO:0003700 | transcription factor activity | 4.47E-14 |
| 36 | 310 | GO:0044238 | primary metabolic process | 8.25E-69 |
| 36 | 310 | GO:0044237 | cellular metabolic process | 5.00E-62 |
| 36 | 310 | GO:0008152 | metabolic process | 6.09E-62 |
| 37 | 343 | GO:0003677 | DNA binding | 4.82E-09 |
| 37 | 343 | GO:0003676 | nucleic acid binding | 1.11E-07 |
| 37 | 343 | GO:0007155 | cell adhesion | 9.52E-05 |
| 38 | 350 | GO:0003824 | catalytic activity | 1.08E-28 |
| 38 | 350 | GO:0008152 | metabolic process | 4.43E-18 |
| 38 | 350 | GO:0016020 | membrane | 5.75E-16 |

The GO::TermFinder software was used in conjunction with a generated Gene Ontology (GO) file for *X. nematophila* to assign GO annotations for each mountain. A total of 32 out of 38 mountains were found to be statistically significant for GO functional enrichment. The top 3 GO terms with a *P* value < 0.05 were retained for each mountain in this analysis.
