# Supplementary material for: The Entomopathogenic Bacterial Endosymbionts Xenorhabdus and Photorhabdus: Convergent Lifestyles from Divergent Genomes
Source: PLoS One. 2011 Nov 18;6(11):e27909. doi: 10.1371/journal.pone.0027909 (PMC3220699; doi:10.1371/journal.pone.0027909)
Supplement: Table S2 — Statistical enrichment of functional groups for each mountain on the Xenorhabdus bovienii phylogenomic map. (DOC) [file pone.0027909.s004.doc]

**Table S2.** Statistical enrichment of functional groups for each mountain on the *Xenorhabdus bovienii* phylogenomic map.

| **Mount** | **No. of**  **Proteins** | **GO ID** | **Term** | ***P*-value** |
| --- | --- | --- | --- | --- |
| 1 | 5 | GO:0005515 | protein binding | 2.17E-07 |
| 1 | 5 | GO:0006259 | DNA metabolic process | 6.19E-05 |
| 1 | 5 | GO:0003677 | DNA binding | 6.69E-05 |
| 2 | 6 | - | - | - |
| 3 | 9 | GO:0003676 | nucleic acid binding | 9.43E-04 |
| 3 | 9 | GO:0005488 | binding | 1.79E-02 |
| 4 | 13 | GO:0006313 | transposition, DNA-mediated | 8.75E-13 |
| 4 | 13 | GO:0032196 | transposition | 8.75E-13 |
| 4 | 13 | GO:0006310 | DNA recombination | 7.27E-12 |
| 5 | 14 | GO:0015074 | DNA integration | 1.05E-08 |
| 5 | 14 | GO:0003677 | DNA binding | 2.74E-04 |
| 5 | 14 | GO:0003676 | nucleic acid binding | 1.09E-03 |
| 6 | 15 | GO:0000155 | two-component sensor activity | 3.98E-04 |
| 6 | 15 | GO:0016775 | phosphotransferase activity, nitrogenous group as acceptor | 5.65E-04 |
| 6 | 15 | GO:0004672 | protein kinase activity | 6.64E-04 |
| 7 | 24 | GO:0006313 | transposition, DNA-mediated | 6.61E-29 |
| 7 | 24 | GO:0032196 | transposition | 6.61E-29 |
| 7 | 24 | GO:0006310 | DNA recombination | 7.96E-27 |
| 8 | 25 | GO:0003677 | DNA binding | 3.71E-02 |
| 9 | 27 | GO:0004803 | transposase activity | 1.61E-10 |
| 9 | 27 | GO:0006313 | transposition, DNA-mediated | 8.40E-09 |
| 9 | 27 | GO:0032196 | transposition | 8.40E-09 |
| 10 | 29 | GO:0004803 | transposase activity | 5.28E-20 |
| 10 | 29 | GO:0006313 | transposition, DNA-mediated | 5.75E-17 |
| 10 | 29 | GO:0032196 | transposition | 5.75E-17 |
| 11 | 30 | GO:0004519 | endonuclease activity | 7.50E-04 |
| 11 | 30 | GO:0004518 | nuclease activity | 3.53E-03 |
| 11 | 30 | GO:0016787 | hydrolase activity | 8.48E-03 |
| 12 | 31 | GO:0005515 | protein binding | 8.72E-09 |
| 12 | 31 | GO:0005488 | binding | 9.72E-05 |
| 13 | 33 | GO:0009236 | cobalamin biosynthetic process | 1.27E-31 |
| 13 | 33 | GO:0006779 | porphyrin biosynthetic process | 4.14E-28 |
| 13 | 33 | GO:0006778 | porphyrin metabolic process | 8.84E-28 |
| 14 | 42 | GO:0040011 | locomotion | 1.06E-39 |
| 14 | 42 | GO:0019861 | flagellum | 7.85E-36 |
| 14 | 42 | GO:0006928 | cell motion | 2.61E-31 |
| 15 | 45 | GO:0016043 | cellular component organization | 7.73E-07 |
| 15 | 45 | GO:0007047 | cell wall organization | 2.70E-06 |
| 15 | 45 | GO:0045229 | external encapsulating structure organization | 6.28E-06 |
| 16 | 46 | GO:0005515 | protein binding | 5.79E-45 |
| 16 | 46 | GO:0003676 | nucleic acid binding | 3.59E-23 |
| 16 | 46 | GO:0005488 | binding | 1.05E-15 |
| 17 | 47 | GO:0055085 | transmembrane transport | 1.04E-22 |
| 17 | 47 | GO:0006810 | transport | 2.07E-19 |
| 17 | 47 | GO:0051234 | establishment of localization | 2.07E-19 |
| 18 | 60 | GO:0004559 | alpha-mannosidase activity | 5.16E-05 |
| 18 | 60 | GO:0015923 | mannosidase activity | 5.16E-05 |
| 18 | 60 | GO:0006306 | DNA methylation | 3.09E-03 |
| 19 | 65 | GO:0050896 | response to stimulus | 1.50E-02 |
| 19 | 65 | GO:0006950 | response to stress | 3.33E-02 |
| 20 | 68 | GO:0007059 | chromosome segregation | 1.00E-03 |
| 20 | 68 | GO:0006996 | organelle organization | 5.88E-03 |
| 20 | 68 | GO:0030261 | chromosome condensation | 5.88E-03 |
| 21 | 79 | GO:0006519 | cellular amino acid and derivative metabolic process | 2.89E-30 |
| 21 | 79 | GO:0006520 | cellular amino acid metabolic process | 2.85E-28 |
| 21 | 79 | GO:0044262 | cellular carbohydrate metabolic process | 1.65E-25 |
| 22 | 83 | GO:0016020 | membrane | 1.70E-13 |
| 22 | 83 | GO:0006810 | transport | 1.74E-08 |
| 22 | 83 | GO:0051234 | establishment of localization | 1.74E-08 |
| 22 | 83 | GO:0051179 | localization | 6.92E-08 |
| 23 | 86 | - | - | - |
| 24 | 88 | GO:0043170 | macromolecule metabolic process | 4.48E-13 |
| 24 | 88 | GO:0043283 | biopolymer metabolic process | 1.68E-12 |
| 24 | 88 | GO:0008134 | transcription factor binding | 1.03E-11 |
| 25 | 93 | GO:0009401 | phosphoenolpyruvate-dependent sugar phosphotransferase system | 8.15E-15 |
| 25 | 93 | GO:0008643 | carbohydrate transport | 1.18E-14 |
| 25 | 93 | GO:0015144 | carbohydrate transmembrane transporter activity | 1.32E-11 |
| 26 | 105 | GO:0010927 | cellular component assembly involved in morphogenesis | 3.01E-10 |
| 26 | 105 | GO:0022414 | reproductive process | 3.01E-10 |
| 26 | 105 | GO:0048646 | anatomical structure formation involved in morphogenesis | 3.01E-10 |
| 27 | 118 | GO:0016020 | membrane | 7.80E-08 |
| 27 | 118 | GO:0016021 | integral to membrane | 2.17E-05 |
| 27 | 118 | GO:0031224 | intrinsic to membrane | 2.17E-05 |
| 28 | 120 | GO:0000036 | acyl carrier activity | 5.77E-33 |
| 28 | 120 | GO:0031177 | phosphopantetheine binding | 2.47E-20 |
| 28 | 120 | GO:0016597 | amino acid binding | 3.39E-15 |
| 29 | 152 | GO:0006313 | transposition, DNA-mediated | 6.94E-189 |
| 29 | 152 | GO:0006310 | DNA recombination | 1.25E-170 |
| 29 | 152 | GO:0006259 | DNA metabolic process | 2.89E-141 |
| 30 | 163 | GO:0008152 | metabolic process | 1.15E-07 |
| 30 | 163 | GO:0003824 | catalytic activity | 1.83E-07 |
| 30 | 163 | GO:0008270 | zinc ion binding | 3.72E-05 |
| 31 | 172 | GO:0044237 | cellular metabolic process | 1.04E-38 |
| 31 | 172 | GO:0009987 | cellular process | 1.52E-36 |
| 31 | 172 | GO:0044260 | cellular macromolecule metabolic process | 7.75E-33 |
| 32 | 174 | GO:0003824 | catalytic activity | 7.39E-06 |
| 32 | 174 | GO:0016020 | membrane | 1.31E-05 |
| 32 | 174 | GO:0008152 | metabolic process | 5.18E-05 |
| 33 | 263 | GO:0051186 | cofactor metabolic process | 5.93E-16 |
| 33 | 263 | GO:0016491 | oxidoreductase activity | 5.15E-15 |
| 33 | 263 | GO:0055114 | oxidation reduction | 1.82E-14 |
| 34 | 275 | GO:0044238 | primary metabolic process | 4.10E-56 |
| 34 | 275 | GO:0044237 | cellular metabolic process | 1.61E-52 |
| 34 | 275 | GO:0008152 | metabolic process | 6.49E-51 |
| 35 | 294 | GO:0030528 | transcription regulator activity | 3.88E-29 |
| 35 | 294 | GO:0003700 | transcription factor activity | 4.97E-29 |
| 35 | 294 | GO:0006355 | regulation of transcription, DNA-dependent | 1.60E-28 |
| 36 | 338 | GO:0007155 | cell adhesion | 6.00E-16 |
| 36 | 338 | GO:0022610 | biological adhesion | 6.00E-16 |
| 36 | 338 | GO:0008219 | cell death | 6.84E-08 |

The GO::TermFinder software was used in conjunction with a generated Gene Ontology (GO) file for *X. bovienii* to assign GO annotations for each mountain. A total of 34 out of 36 mountains were found to be statistically significant for GO functional enrichment. The top 3 GO terms with a *P* value < 0.05 were retained for each mountain in this analysis.
