# Supplementary material for: The Entomopathogenic Bacterial Endosymbionts Xenorhabdus and Photorhabdus: Convergent Lifestyles from Divergent Genomes
Source: PLoS One. 2011 Nov 18;6(11):e27909. doi: 10.1371/journal.pone.0027909 (PMC3220699; doi:10.1371/journal.pone.0027909)
Supplement: Table S3 — Statistical enrichment of functional groups for each mountain on the Photorhabdus luminescens phylogenomic map. (DOC) [file pone.0027909.s005.doc]

**Table S3.** Statistical enrichment of functional groups for each mountain on the *Photorhabdus luminescens* phylogenomic map.

| **Mount** | **No. of**  **Proteins** | **GOID** | **Term** | ***P*-value** |
| --- | --- | --- | --- | --- |
| 1 | 2 | GO:0043565 | sequence-specific DNA binding | 3.11E-03 |
| 1 | 2 | GO:0003677 | DNA binding | 4.53E-02 |
| 2 | 6 | GO:0016788 | hydrolase activity, acting on ester bonds | 1.70E-02 |
| 3 | 7 | GO:0004803 | transposase activity | 1.04E-13 |
| 3 | 7 | GO:0006313 | transposition, DNA-mediated | 3.33E-13 |
| 3 | 7 | GO:0006310 | DNA recombination | 2.47E-11 |
| 4 | 8 | - | - |  |
| 5 | 9 | GO:0043565 | sequence-specific DNA binding | 3.73E-07 |
| 5 | 9 | GO:0003677 | DNA binding | 2.95E-04 |
| 5 | 9 | GO:0003676 | nucleic acid binding | 9.49E-04 |
| 6 | 10 | - | - |  |
| 7 | 11 | GO:0003677 | DNA binding | 4.08E-02 |
| 8 | 12 | GO:0006810 | transport | 1.19E-02 |
| 8 | 12 | GO:0051234 | establishment of localization | 1.19E-02 |
| 8 | 12 | GO:0055085 | transmembrane transport | 2.63E-02 |
| 9 | 13 | GO:0015074 | DNA integration | 7.73E-09 |
| 9 | 13 | GO:0004803 | transposase activity | 7.89E-09 |
| 9 | 13 | GO:0006313 | transposition, DNA-mediated | 1.57E-08 |
| 10 | 14 | GO:0043226 | organelle | 2.33E-02 |
| 10 | 14 | GO:0043229 | intracellular organelle | 2.33E-02 |
| 10 | 14 | GO:0044444 | cytoplasmic part | 2.57E-02 |
| 11 | 21 | GO:0017111 | nucleoside-triphosphatase activity | 9.77E-05 |
| 11 | 21 | GO:0016462 | pyrophosphatase activity | 1.27E-04 |
| 11 | 21 | GO:0016818 | hydrolase activity, acting on acid anhydrides, in phosphorus-containing anhydrides | 1.40E-04 |
| 12 | 25 | GO:0005488 | binding | 4.78E-03 |
| 13 | 26 | GO:0004803 | transposase activity | 4.99E-34 |
| 13 | 26 | GO:0006313 | transposition, DNA-mediated | 2.01E-33 |
| 13 | 26 | GO:0006310 | DNA recombination | 1.64E-28 |
| 14 | 28 | - | - | - |
| 15 | 36 | - | - | - |
| 16 | 39 | GO:0003676 | nucleic acid binding | 9.21E-08 |
| 16 | 39 | GO:0003677 | DNA binding | 2.21E-07 |
| 16 | 39 | GO:0004803 | transposase activity | 6.76E-05 |
| 17 | 42 | GO:0005515 | protein binding | 1.10E-63 |
| 17 | 42 | GO:0015074 | DNA integration | 1.15E-15 |
| 17 | 42 | GO:0006259 | DNA metabolic process | 3.38E-07 |
| 18 | 43 | GO:0009235 | cobalamin metabolic process | 1.06E-22 |
| 18 | 43 | GO:0009236 | cobalamin biosynthetic process | 1.06E-22 |
| 18 | 43 | GO:0006779 | porphyrin biosynthetic process | 1.03E-19 |
| 19 | 45 | GO:0016641 | oxidoreductase activity, acting on the CH-NH2 group of donors, oxygen as acceptor | 7.08E-11 |
| 19 | 45 | GO:0048038 | quinone binding | 7.08E-11 |
| 19 | 45 | GO:0005507 | copper ion binding | 3.81E-10 |
| 20 | 47 | - | - | - |
| 21 | 52 | GO:0003677 | DNA binding | 4.51E-04 |
| 21 | 52 | GO:0003676 | nucleic acid binding | 1.74E-03 |
| 21 | 52 | GO:0043565 | sequence-specific DNA binding | 7.94E-03 |
| 22 | 53 | GO:0019861 | flagellum | 4.46E-29 |
| 22 | 53 | GO:0006928 | cell motion | 3.54E-25 |
| 22 | 53 | GO:0051674 | localization of cell | 3.54E-25 |
| 23 | 57 | GO:0006519 | cellular amino acid and derivative metabolic process | 1.08E-35 |
| 23 | 57 | GO:0044106 | cellular amine metabolic process | 6.59E-35 |
| 23 | 57 | GO:0019752 | carboxylic acid metabolic process | 1.77E-32 |
| 24 | 61 | GO:0000271 | polysaccharide biosynthetic process | 4.70E-02 |
| 25 | 64 | - | - | - |
| 26 | 68 | GO:0003676 | nucleic acid binding | 5.91E-03 |
| 26 | 68 | GO:0005488 | binding | 1.29E-02 |
| 26 | 68 | GO:0003677 | DNA binding | 4.08E-02 |
| 27 | 77 | GO:0005623 | cell | 1.16E-04 |
| 27 | 77 | GO:0044464 | cell part | 1.16E-04 |
| 27 | 77 | GO:0007059 | chromosome segregation | 4.66E-03 |
| 28 | 78 | GO:0000156 | two-component response regulator activity | 7.83E-55 |
| 28 | 78 | GO:0000160 | two-component signal transduction system (phosphorelay) | 3.47E-52 |
| 28 | 78 | GO:0004871 | signal transducer activity | 2.09E-47 |
| 29 | 89 | GO:0005102 | receptor binding | 7.95E-07 |
| 29 | 89 | GO:0009607 | response to biotic stimulus | 3.17E-06 |
| 29 | 89 | GO:0051707 | response to other organism | 3.17E-06 |
| 30 | 91 | GO:0005230 | extracellular ligand-gated ion channel activity | 2.46E-08 |
| 30 | 91 | GO:0015276 | ligand-gated ion channel activity | 2.46E-08 |
| 30 | 91 | GO:0022836 | gated channel activity | 2.46E-08 |
| 31 | 101 | GO:0000036 | acyl carrier activity | 3.21E-32 |
| 31 | 101 | GO:0031177 | phosphopantetheine binding | 5.91E-30 |
| 31 | 101 | GO:0016597 | amino acid binding | 1.17E-24 |
| 32 | 105 | GO:0009401 | phosphoenolpyruvate-dependent sugar phosphotransferase system | 2.55E-32 |
| 32 | 105 | GO:0015144 | carbohydrate transmembrane transporter activity | 1.84E-21 |
| 32 | 105 | GO:0015294 | solute:cation symporter activity | 1.10E-20 |
| 33 | 108 | GO:0007047 | cell wall organization | 4.65E-24 |
| 33 | 108 | GO:0045229 | external encapsulating structure organization | 2.30E-22 |
| 33 | 108 | GO:0030288 | outer membrane-bounded periplasmic space | 9.41E-16 |
| 34 | 116 | GO:0016020 | membrane | 2.44E-10 |
| 34 | 116 | GO:0022900 | electron transport chain | 3.66E-05 |
| 34 | 116 | GO:0006814 | sodium ion transport | 6.18E-05 |
| 35 | 142 | GO:0009405 | pathogenesis | 4.21E-21 |
| 35 | 142 | GO:0044403 | symbiosis, encompassing mutualism through parasitism | 4.21E-21 |
| 35 | 142 | GO:0044419 | interspecies interaction between organisms | 4.21E-21 |
| 36 | 147 | GO:0003824 | catalytic activity | 5.45E-19 |
| 36 | 147 | GO:0044237 | cellular metabolic process | 1.21E-16 |
| 36 | 147 | GO:0009987 | cellular process | 9.42E-16 |
| 37 | 162 | GO:0044459 | plasma membrane part | 4.51E-06 |
| 37 | 162 | GO:0015173 | aromatic amino acid transmembrane transporter activity | 3.09E-05 |
| 37 | 162 | GO:0019752 | carboxylic acid metabolic process | 4.99E-05 |
| 38 | 181 | GO:0005215 | transporter activity | 3.16E-46 |
| 38 | 181 | GO:0051234 | establishment of localization | 2.08E-31 |
| 38 | 181 | GO:0016887 | ATPase activity | 1.97E-17 |
| 39 | 195 | GO:0005975 | carbohydrate metabolic process | 1.95E-04 |
| 39 | 195 | GO:0008653 | lipopolysaccharide metabolic process | 3.52E-04 |
| 39 | 195 | GO:0009103 | lipopolysaccharide biosynthetic process | 3.52E-04 |
| 40 | 245 | GO:0044260 | cellular macromolecule metabolic process | 1.32E-44 |
| 40 | 245 | GO:0034960 | cellular biopolymer metabolic process | 1.07E-43 |
| 40 | 245 | GO:0043283 | biopolymer metabolic process | 5.09E-41 |
| 41 | 306 | GO:0044237 | cellular metabolic process | 2.24E-64 |
| 41 | 306 | GO:0008152 | metabolic process | 2.91E-64 |
| 41 | 306 | GO:0043170 | macromolecule metabolic process | 5.10E-46 |
| 42 | 333 | GO:0003824 | catalytic activity | 4.56E-23 |
| 42 | 333 | GO:0016020 | membrane | 1.68E-19 |
| 42 | 333 | GO:0008565 | protein transporter activity | 1.19E-11 |
| 43 | 368 | GO:0003677 | DNA binding | 5.12E-11 |
| 43 | 368 | GO:0006139 | nucleobase, nucleoside, nucleotide and nucleic acid metabolic process | 7.15E-09 |
| 43 | 368 | GO:0034960 | cellular biopolymer metabolic process | 4.09E-08 |
| 44 | 383 | GO:0008152 | metabolic process | 1.80E-32 |
| 44 | 383 | GO:0016491 | oxidoreductase activity | 6.45E-30 |
| 44 | 383 | GO:0003700 | transcription factor activity | 1.72E-22 |
| 44 | 383 | GO:0030528 | transcription regulator activity | 1.45E-19 |

The GO::TermFinder software was used in conjunction with a generated Gene Ontology (GO) file for *P. luminescens* to assign GO annotations for each mountain. A total of 39 out of 44 mountains were found to be statistically significant for GO functional enrichment. The top 3 GO terms with a *P* value < 0.05 were retained for each mountain in this analysis.
