# Supplementary material for: The Entomopathogenic Bacterial Endosymbionts Xenorhabdus and Photorhabdus: Convergent Lifestyles from Divergent Genomes
Source: PLoS One. 2011 Nov 18;6(11):e27909. doi: 10.1371/journal.pone.0027909 (PMC3220699; doi:10.1371/journal.pone.0027909)
Supplement: Table S4 — Statistical enrichment of functional groups for each mountain on the Photorhabdus asymbiotica phylogenomic map. (DOC) [file pone.0027909.s006.doc]

**Table S4.** Statistical enrichment of functional groups for each mountain on the *Photorhabdus asymbiotica* phylogenomic map.

| **Mount** | **No. of**  **Proteins** | **GOID** | **Term** | ***P*-value** |
| --- | --- | --- | --- | --- |
| 1 | 4 | GO:0006304 | DNA modification | 9.82E-09 |
| 1 | 4 | GO:0043412 | biopolymer modification | 2.60E-06 |
| 1 | 4 | GO:0009007 | site-specific DNA-methyltransferase (adenine-specific) activity | 1.89E-04 |
| 2 | 7 | - | - | - |
| 3 | 8 | - | - | - |
| 4 | 11 | - | - | - |
| 4 | 11 | GO:0004803 | transposase activity | 5.75E-10 |
| 4 | 11 | GO:0006313 | transposition, DNA-mediated | 2.10E-09 |
| 4 | 11 | GO:0006310 | DNA recombination | 4.02E-07 |
| 5 | 18 | - | - | - |
| 6 | 24 | - | - | - |
| 7 | 28 | GO:0009405 | pathogenesis | 2.17E-25 |
| 7 | 28 | GO:0044403 | symbiosis, encompassing mutualism through parasitism | 2.17E-25 |
| 7 | 28 | GO:0044419 | interspecies interaction between organisms | 2.17E-25 |
| 8 | 30 | - | - | - |
| 8 | 30 | GO:0003824 | catalytic activity | 1.28E-02 |
| 9 | 31 | GO:0010927 | cellular component assembly involved in morphogenesis | 2.55E-10 |
| 9 | 31 | GO:0019067 | viral assembly, maturation, egress, and release | 2.55E-10 |
| 9 | 31 | GO:0048646 | anatomical structure formation involved in morphogenesis | 2.55E-10 |
| 10 | 39 | - | - | - |
| 11 | 40 | GO:0043565 | sequence-specific DNA binding | 9.81E-06 |
| 11 | 40 | GO:0003677 | DNA binding | 1.05E-04 |
| 11 | 40 | GO:0003676 | nucleic acid binding | 4.64E-04 |
| 12 | 41 | GO:0016539 | intein-mediated protein splicing | 3.16E-05 |
| 12 | 41 | GO:0016485 | protein processing | 1.89E-04 |
| 12 | 41 | GO:0051604 | protein maturation | 3.15E-04 |
| 13 | 44 | GO:0005789 | endoplasmic reticulum membrane | 1.53E-06 |
| 13 | 44 | GO:0012505 | endomembrane system | 1.53E-06 |
| 13 | 44 | GO:0031227 | intrinsic to endoplasmic reticulum membrane | 1.53E-06 |
| 13 | 44 | GO:0031300 | intrinsic to organelle membrane | 1.53E-06 |
| 14 | 48 | GO:0008080 | N-acetyltransferase activity | 1.84E-02 |
| 14 | 48 | GO:0016787 | hydrolase activity | 3.05E-02 |
| 14 | 48 | GO:0016407 | acetyltransferase activity | 3.11E-02 |
| 15 | 49 | GO:0040011 | locomotion | 1.36E-36 |
| 15 | 49 | GO:0019861 | flagellum | 1.26E-32 |
| 15 | 49 | GO:0051674 | localization of cell | 6.03E-29 |
| 16 | 54 | - | - | - |
| 17 | 56 | GO:0004806 | triacylglycerol lipase activity | 1.18E-07 |
| 17 | 56 | GO:0004091 | carboxylesterase activity | 3.93E-07 |
| 17 | 56 | GO:0016788 | hydrolase activity, acting on ester bonds | 2.64E-06 |
| 18 | 63 | GO:0006519 | cellular amino acid and derivative metabolic process | 6.22E-34 |
| 18 | 63 | GO:0044262 | cellular carbohydrate metabolic process | 1.11E-29 |
| 18 | 63 | GO:0009308 | amine metabolic process | 1.57E-29 |
| 19 | 64 | GO:0007059 | chromosome segregation | 4.57E-04 |
| 19 | 64 | GO:0006996 | organelle organization | 3.30E-03 |
| 19 | 64 | GO:0030261 | chromosome condensation | 3.30E-03 |
| 20 | 70 | GO:0000156 | two-component response regulator activity | 3.56E-21 |
| 20 | 70 | GO:0000160 | two-component signal transduction system (phosphorelay) | 1.42E-19 |
| 20 | 70 | GO:0004871 | signal transducer activity | 1.73E-17 |
| 21 | 76 | GO:0005198 | structural molecule activity | 7.22E-10 |
| 21 | 76 | GO:0008131 | amine oxidase activity | 3.47E-08 |
| 21 | 76 | GO:0016641 | oxidoreductase activity, acting on the CH-NH2 group of donors, oxygen as acceptor | 1.26E-07 |
| 22 | 78 | GO:0031177 | phosphopantetheine binding | 9.42E-28 |
| 22 | 78 | GO:0022892 | substrate-specific transporter activity | 4.63E-25 |
| 22 | 78 | GO:0016597 | amino acid binding | 8.88E-23 |
| 23 | 81 | GO:0005215 | transporter activity | 6.79E-42 |
| 23 | 81 | GO:0051234 | establishment of localization | 4.03E-29 |
| 23 | 81 | GO:0016887 | ATPase activity | 7.20E-20 |
| 24 | 91 | GO:0015144 | carbohydrate transmembrane transporter activity | 4.71E-23 |
| 24 | 91 | GO:0051119 | sugar transmembrane transporter activity | 3.88E-19 |
| 24 | 91 | GO:0005402 | cation:sugar symporter activity | 4.86E-18 |
| 25 | 101 | GO:0005342 | organic acid transmembrane transporter activity | 2.46E-11 |
| 25 | 101 | GO:0046943 | carboxylic acid transmembrane transporter activity | 1.01E-08 |
| 25 | 101 | GO:0005275 | amine transmembrane transporter activity | 1.29E-08 |
| 25 | 101 | GO:0003700 | transcription factor activity | 1.40E-07 |
| 26 | 107 | GO:0010181 | FMN binding | 3.38E-05 |
| 26 | 107 | GO:0048029 | monosaccharide binding | 4.76E-05 |
| 26 | 107 | GO:0022900 | electron transport chain | 5.51E-05 |
| 27 | 120 | GO:0008565 | protein transporter activity | 2.48E-16 |
| 27 | 120 | GO:0051234 | establishment of localization | 3.92E-12 |
| 27 | 120 | GO:0005215 | transporter activity | 8.82E-12 |
| 28 | 124 | GO:0005102 | receptor binding | 8.49E-09 |
| 28 | 124 | GO:0009607 | response to biotic stimulus | 3.78E-08 |
| 28 | 124 | GO:0051707 | response to other organism | 3.78E-08 |
| 29 | 137 | GO:0015643 | toxin binding | 2.11E-07 |
| 29 | 137 | GO:0030153 | bacteriocin immunity | 4.73E-07 |
| 29 | 137 | GO:0004519 | endonuclease activity | 1.33E-05 |
| 30 | 159 | GO:0003824 | catalytic activity | 3.42E-05 |
| 30 | 159 | GO:0044237 | cellular metabolic process | 5.02E-04 |
| 30 | 159 | GO:0005975 | carbohydrate metabolic process | 6.59E-04 |
| 31 | 169 | GO:0005488 | binding | 2.84E-05 |
| 31 | 169 | GO:0044238 | primary metabolic process | 3.45E-05 |
| 31 | 169 | GO:0008152 | metabolic process | 6.73E-05 |
| 32 | 178 | GO:0044260 | cellular macromolecule metabolic process | 3.50E-45 |
| 32 | 178 | GO:0034960 | cellular biopolymer metabolic process | 5.83E-44 |
| 32 | 178 | GO:0044237 | cellular metabolic process | 1.52E-42 |
| 33 | 320 | GO:0030528 | transcription regulator activity | 2.07E-29 |
| 33 | 320 | GO:0050794 | regulation of cellular process | 6.76E-28 |
| 33 | 320 | GO:0006355 | regulation of transcription, DNA-dependent | 1.61E-27 |
| 34 | 339 | GO:0044238 | primary metabolic process | 8.48E-84 |
| 34 | 339 | GO:0044237 | cellular metabolic process | 4.65E-67 |
| 34 | 339 | GO:0043170 | macromolecule metabolic process | 1.75E-60 |
| 35 | 378 | GO:0009289 | pilus | 1.73E-06 |
| 35 | 378 | GO:0007155 | cell adhesion | 4.11E-06 |
| 35 | 378 | GO:0003676 | nucleic acid binding | 5.57E-06 |

The GO::TermFinder software was used in conjunction with a generated Gene Ontology (GO) file for *P. asymbiotica* to assign GO annotations for each mountain. A total of 28 out of 35 mountains were found to be statistically significant for GO functional enrichment. The top 3 GO terms with a *P* value < 0.05 were retained for each mountain in this analysis.
