# Supplementary material for: The Entomopathogenic Bacterial Endosymbionts Xenorhabdus and Photorhabdus: Convergent Lifestyles from Divergent Genomes
Source: PLoS One. 2011 Nov 18;6(11):e27909. doi: 10.1371/journal.pone.0027909 (PMC3220699; doi:10.1371/journal.pone.0027909)
Supplement: Table S5 — Gene identities and annotations found within mountains on a phylogenomic map constructed for orthologous genes found between the Xenorhabdus and Photorhabdus genomes but not in Salmonella typhimurium LT2, or Escherichia coli K12. (DOC) [file pone.0027909.s007.doc]

**Table S5.** Gene identities and annotations found within mountains on a phylogenomic map constructed for orthologous genes found between the *Xenorhabdus* and *Photorhabdus* genomes but not in *Salmonella typhimurium* LT2, or *Escherichia coli* K12.

| **Gene ID** | **Length** | **Mountain** | **Protein ID** | **Annotation** |
| --- | --- | --- | --- | --- |
| XNC1_0065 | 210 | XP1 | 300721147 | hypothetical protein |
| XNC1_0075 | 246 | XP1 | 300721157 | conserved hypothetical protein; putative exported protein |
| XNC1_0107 | 147 | XP1 | 300721187 | hypothetical protein |
| XNC1_0157 | 317 | XP1 | 300721232 | WalW protein |
| XNC1_0604 | 337 | XP1 | 300721635 | putative Gamma-butyrobetaine dioxygenase |
| XNC1_0774 | 102 | XP1 | 300721789 | putative transcriptional regulator (LuxR family, partial) |
| XNC1_0917 | 83 | XP1 | 300721926 | transposase (fragment) |
| XNC1_0925 | 434 | XP1 | 300721934 | hypothetical protein |
| XNC1_1015 | 74 | XP1 | 300722022 | putative phage integrase |
| XNC1_1183 | 70 | XP1 | 300722176 | hypothetical protein |
| XNC1_1201 | 215 | XP1 | 300722193 | hypothetical protein |
| XNC1_1379 | 121 | XP1 | 300722356 | hypothetical protein |
| XNC1_1382 | 170 | XP1 | 300722359 | hypothetical protein |
| XNC1_1473 | 51 | XP1 | 300722446 | conserved hypothetical protein; putative exported protein |
| XNC1_1531 | 56 | XP1 | 300722502 | hypothetical protein |
| XNC1_2015 | 125 | XP1 | 300722959 | PA-I galactophilic lectin (PA-IL) (Galactose-binding lectin) |
| XNC1_2031 | 101 | XP1 | 300722975 | hypothetical protein |
| XNC1_2125 | 121 | XP1 | 300723067 | putative PA-I galactophilic lectin (PA-IL) (Galactose-binding lectin) |
| XNC1_2260 | 74 | XP1 | 300723196 | hypothetical protein |
| XNC1_2416 | 273 | XP1 | 300723339 | hypothetical protein |
| XNC1_2473 | 157 | XP1 | 300723392 | hypothetical protein |
| XNC1_2642 | 55 | XP1 | 300723537 | putative outer membrane siderophore receptor CjrC |
| XNC1_2779 | 58 | XP1 | 300723667 | hypothetical protein |
| XNC1_3279 | 55 | XP1 | 300724132 | hypothetical protein |
| XNC1_3323 | 72 | XP1 | 300724176 | hypothetical protein |
| XNC1_3330 | 45 | XP1 | 300724182 | hypothetical protein |
| XNC1_3766 | 408 | XP1 | 300724567 | XaxA |
| XNC1_3767 | 350 | XP1 | 300724568 | XaxB |
| XNC1_3831 | 121 | XP1 | 300724627 | conserved hypothetical protein; putative exported protein |
| XNC1_3900 | 97 | XP1 | 300724691 | conserved hypothetical protein; putative exported protein |
| XNC1_3947 | 188 | XP1 | 300724738 | hypothetical protein |
| XNC1_3958 | 119 | XP1 | 300724749 | hypothetical protein |
| XNC1_4126 | 98 | XP1 | 300724906 | hypothetical protein |
| XNC1_4162 | 50 | XP1 | 300724938 | hypothetical protein |
| XNC1_4248 | 64 | XP1 | 300725019 | hypothetical protein |
| XNC1_4347 | 157 | XP1 | 300725111 | hypothetical protein |
| XNC1_4417 | 489 | XP1 | 300725175 | hypothetical protein |
| XNC1_4599 | 309 | XP1 | 300725344 | hypothetical protein |
| XNC1_4602 | 376 | XP1 | 300725347 | putative Long-chain-fatty-acid--luciferin-component ligase |
| XNC1_4603 | 49 | XP1 | 300725348 | hypothetical protein |
| XNC1_2055 | 131 | XP2 | 300722999 | hypothetical protein |
| XNC1_3075 | 131 | XP2 | 300723934 | hypothetical protein |
| XNC1_3132 | 153 | XP2 | 300723990 | hypothetical protein |
| XNC1_3596 | 228 | XP2 | 300724413 | hypothetical protein |
| XNC1_3727 | 331 | XP2 | 300724530 | conserved hypothetical protein; putative exported protein |
| XNC1_3728 | 383 | XP2 | 300724531 | conserved hypothetical protein; putative exported protein |
| XNC1_3729 | 373 | XP2 | 300724532 | hypothetical protein |
| XNC1_p0137 | 145 | XP3 | 296491949 | hypothetical protein |
| XNC1_0096 | 294 | XP3 | 300721178 | transposase |
| XNC1_0167 | 294 | XP3 | 300721242 | transposase |
| XNC1_1179 | 294 | XP3 | 300722172 | transposase |
| XNC1_2098 | 294 | XP3 | 300723040 | transposase |
| XNC1_2863 | 294 | XP3 | 300723742 | transposase |
| XNC1_3630 | 294 | XP3 | 300724443 | transposase |
| XNC1_3696 | 294 | XP3 | 300724501 | transposase |
| XNC1_4268 | 294 | XP3 | 300725036 | transposase |
| XNC1_4579 | 294 | XP3 | 300725325 | transposase |
| XNC1_4598 | 294 | XP3 | 300725343 | transposase |
| XNC1_0066 | 182 | XP4 | 300721148 | conserved hypothetical protein; putative exported protein |
| XNC1_0072 | 267 | XP4 | 300721154 | putative Protein tonB |
| XNC1_0676 | 117 | XP4 | 300721699 | Putative membrane protein (fragment) |
| XNC1_0677 | 61 | XP4 | 300721700 | hypothetical protein |
| XNC1_0772 | 85 | XP4 | 300721787 | putative transcriptional regulator LuxR family (partial) |
| XNC1_0861 | 160 | XP4 | 300721872 | Putative membrane protein precursor (fragment) |
| XNC1_1065 | 178 | XP4 | 300722065 | hypothetical protein |
| XNC1_1177 | 164 | XP4 | 300722170 | hypothetical protein |
| XNC1_1381 | 4970 | XP4 | 300722358 | toxin RtxA |
| XNC1_1957 | 424 | XP4 | 300722903 | hypothetical protein |
| XNC1_2096 | 210 | XP4 | 300723038 | Complete genome; segment 8/17 |
| XNC1_2186 | 1516 | XP4 | 300723127 | B component of insecticidal toxin complex (Tc) |
| XNC1_2187 | 2471 | XP4 | 300723128 | partially similar to A component of insecticidal toxin complex (Tc) |
| XNC1_2188 | 1030 | XP4 | 300723129 | C component of insecticidal toxin complex (Tc) |
| XNC1_2275 | 273 | XP4 | 300723211 | hypothetical protein |
| XNC1_2334 | 1541 | XP4 | 300723268 | A component of insecticidal toxin complex (Tc) (fragment) |
| XNC1_2335 | 1519 | XP4 | 300723269 | B component of insecticidal toxin complex (Tc) |
| XNC1_2336 | 969 | XP4 | 300723270 | C component of insecticidal toxin complex (Tc) |
| XNC1_2560 | 1156 | XP4 | 300723467 | A component of insecticidal toxin complex (Tc) (fragment) |
| XNC1_2561 | 1391 | XP4 | 300723468 | A component of insecticidal toxin complex (Tc) (fragment) |
| XNC1_2566 | 2523 | XP4 | 300723473 | A component of insecticidal toxin complex (Tc) |
| XNC1_2567 | 1016 | XP4 | 300723474 | C component of insecticidal toxin complex (Tc) |
| XNC1_2568 | 1475 | XP4 | 300723475 | B component of insecticidal toxin complex (Tc) |
| XNC1_2569 | 2524 | XP4 | 300723476 | A component of insecticidal toxin complex (Tc) |
| XNC1_2648 | 267 | XP4 | 300723543 | TonB-like protein |
| XNC1_2761 | 377 | XP4 | 300723656 | conserved hypothetical protein; putative exported protein |
| XNC1_2830 | 91 | XP4 | 300723709 | putative lipoprotein |
| XNC1_2893 | 123 | XP4 | 300723769 | putative Cryptic phage CTXphi transcriptional repressor rstR |
| XNC1_2904 | 123 | XP4 | 300723780 | putative Cryptic phage CTXphi transcriptional repressor rstR |
| XNC1_2977 | 231 | XP4 | 300723843 | LuxR-family transcriptional regulator |
| XNC1_3264 | 374 | XP4 | 300724117 | FliA regulated lipase |
| XNC1_3333 | 219 | XP4 | 300724185 | Putative lipoprotein |
| XNC1_3334 | 118 | XP4 | 300724186 | Putative lipoprotein |
| XNC1_3335 | 223 | XP4 | 300724187 | Putative lipoprotein |
| XNC1_3921 | 131 | XP4 | 300724712 | hypothetical protein |
| XNC1_3923 | 47 | XP4 | 300724714 | Putative membrane protein precursor (fragment) |
| XNC1_4024 | 110 | XP4 | 300724812 | Alkaline proteinase inhibitor precursor (PrtA-specific inhibitor) (fragment) |
| XNC1_4025 | 478 | XP4 | 300724813 | Secreted alkaline metalloproteinase |
| XNC1_4039 | 127 | XP4 | 300724827 | Prepilin peptidase dependent protein C |
| XNC1_4040 | 161 | XP4 | 300724828 | hypothetical protein |
| XNC1_4171 | 169 | XP4 | 300724947 | conserved hypothetical protein; putative exported protein |
| XNC1_4542 | 265 | XP4 | 300725295 | Conserved Hypothetical protein (probable component of SSt VI cluster) |
| XNC1_4558 | 68 | XP4 | 300725310 | putative tautomerase K2 |
| XNC1_p0124 | 291 | XP5 | 296491936 | hypothetical protein |
| XNC1_2950 | 362 | XP5 | 300723818 | putative periplasmic protein |
| XNC1_0074 | 491 | XP6 | 300721156 | conserved hypothetical protein; putative exported protein |
| XNC1_1174 | 226 | XP6 | 300722167 | hypothetical protein |
| XNC1_0012 | 482 | XP7 | 300721101 | putative monooxygenase, flavin-binding family |
| XNC1_0067 | 287 | XP7 | 300721149 | hypothetical protein |
| XNC1_0073 | 992 | XP7 | 300721155 | putative receptor protein |
| XNC1_0163 | 269 | XP7 | 300721238 | Lipopolysaccharide core biosynthesis glycosyl transferase kdtX |
| XNC1_0171 | 334 | XP7 | 300721246 | WalO protein |
| XNC1_0540 | 92 | XP7 | 300721571 | Predicted transcriptional regulator |
| XNC1_0793 | 150 | XP7 | 300721808 | 3-dehydroquinate dehydratase, type II |
| XNC1_0924 | 267 | XP7 | 300721933 | conserved hypothetical protein; putative exported protein |
| XNC1_1026 | 305 | XP7 | 300722030 | hypothetical protein |
| XNC1_1044 | 600 | XP7 | 300722046 | putative long-chain-fatty-acid--CoA ligase (Long-chain acyl-CoA synthetase) (LACS) |
| XNC1_1176 | 271 | XP7 | 300722169 | Exoenzyme S synthesis regulatory protein exsA |
| XNC1_1178 | 89 | XP7 | 300722171 | hypothetical protein |
| XNC1_1193 | 728 | XP7 | 300722185 | hypothetical protein |
| XNC1_1221 | 335 | XP7 | 300722213 | Pyoverdine biosynthesis protein |
| XNC1_1222 | 296 | XP7 | 300722214 | Pyoverdine biosynthesis protein |
| XNC1_1235 | 817 | XP7 | 300722227 | Heme/hemopexin utilization protein C precursor |
| XNC1_1359 | 899 | XP7 | 300722341 | Chitobiase precursor (N-acetyl-beta-glucosaminidase) (Beta-N-acetylhexosaminidase) |
| XNC1_1372 | 289 | XP7 | 300722349 | Putative type I secretion protein |
| XNC1_1375 | 329 | XP7 | 300722352 | conserved hypothetical protein; putative exported protein |
| XNC1_1377 | 475 | XP7 | 300722354 | RTX toxin ABC transporter protein RtxD |
| XNC1_1380 | 153 | XP7 | 300722357 | RTX toxin activating protein |
| XNC1_1402 | 373 | XP7 | 300722379 | hypothetical protein |
| XNC1_1403 | 256 | XP7 | 300722380 | hypothetical protein |
| XNC1_1482 | 204 | XP7 | 300722455 | hypothetical protein |
| XNC1_1487 | 394 | XP7 | 300722460 | hypothetical protein |
| XNC1_1856 | 357 | XP7 | 300722808 | Hemin transport protein hmuS |
| XNC1_1925 | 317 | XP7 | 300722872 | Proline iminopeptidase (PIP) (Prolyl aminopeptidase) (PAP) |
| XNC1_2039 | 2242 | XP7 | 300722983 | Non-ribosomal peptide synthetase (fragment) |
| XNC1_2130 | 126 | XP7 | 300723072 | putative Fosfomycin resistance protein fosX |
| XNC1_2258 | 668 | XP7 | 300723194 | putative outer membrane copper receptor (OprC) |
| XNC1_2391 | 118 | XP7 | 300723315 | hypothetical protein |
| XNC1_2442 | 568 | XP7 | 300723363 | hypothetical protein |
| XNC1_2494 | 287 | XP7 | 300723412 | hypothetical protein |
| XNC1_2647 | 132 | XP7 | 300723542 | putative Oligopeptidase (Fragment) |
| XNC1_2744 | 208 | XP7 | 300723639 | hypothetical protein |
| XNC1_3045 | 294 | XP7 | 300723906 | hypothetical protein |
| XNC1_3170 | 304 | XP7 | 300724025 | hypothetical protein |
| XNC1_3282 | 213 | XP7 | 300724135 | putative DNA-3-methyladenine glycosylase II |
| XNC1_3502 | 307 | XP7 | 300724335 | hypothetical protein |
| XNC1_3564 | 1554 | XP7 | 300724386 | Putative TpsA-related protein |
| XNC1_3595 | 307 | XP7 | 300724412 | hypothetical protein |
| XNC1_3799 | 190 | XP7 | 300724597 | Fimbrial adaptor, MrxG |
| XNC1_3952 | 339 | XP7 | 300724743 | putative integrase |
| XNC1_4021 | 465 | XP7 | 300724809 | Alkaline protease secretion protein aprF |
| XNC1_4022 | 445 | XP7 | 300724810 | Alkaline protease secretion protein aprE |
| XNC1_4023 | 598 | XP7 | 300724811 | Alkaline protease secretion ATP-binding protein aprD |
| XNC1_4144 | 198 | XP7 | 300724920 | hypothetical protein |
| XNC1_4281 | 353 | XP7 | 300725049 | putative L-iditol 2-dehydrogenase |
| XNC1_4538 | 466 | XP7 | 300725291 | Conserved Hypothetical protein with ImpA domain (probable component of SST VI cluster) |
| XNC1_4539 | 1201 | XP7 | 300725292 | Conserved hypothetical protein (probable component of SST VI cluster) |
| XNC1_4540 | 480 | XP7 | 300725293 | Conserved hypothetical protein with ImpA domain(probable component of SST VI cluster) |
| XNC1_4541 | 223 | XP7 | 300725294 | conserved hypothetical protein (probable component of SST VI cluster) |
| XNC1_4545 | 255 | XP7 | 300725297 | conserved hypothetical protein (probable component of SST VI cluster) |
| XNC1_4547 | 184 | XP7 | 300725299 | conserved hypothetical protein (probable lipoprotein of SST VI cluster) |
| XNC1_4548 | 431 | XP7 | 300725300 | Conserved hypothetical protein with FHA domain (probable component of SST VI cluster) |
| XNC1_4549 | 350 | XP7 | 300725301 | conserved hypothetical protein (probable component of the SST VI cluster) |
| XNC1_4550 | 610 | XP7 | 300725302 | conserved hypothetical protein (probable component of the SST VI cluster) |
| XNC1_4551 | 146 | XP7 | 300725303 | conserved hypothetical protein (probable component of the SST VI cluster; lysozyme-related protein) |
| XNC1_4554 | 172 | XP7 | 300725306 | Hemolysin-coregulated protein Hcp(probable Type VI secreted cytotoxin system component) |
| XNC1_4555 | 557 | XP7 | 300725307 | XhlB, XhlA hemolysin secretion/activation protein (TpsB) |
| XNC1_4556 | 1470 | XP7 | 300725308 | XhlA, Cell surface associated hemolysin (TpsA) |
| XNC1_0088 | 549 | XP8 | 300721170 | hypothetical protein |
| XNC1_0769 | 215 | XP8 | 300721784 | Pyrrolidone-carboxylate peptidase 2 (5-oxoprolyl-peptidase 2) (Pyroglutamyl-peptidase I 2) (PGP-I 2) (Pyrase 2) |
| XNC1_1223 | 428 | XP8 | 300722215 | hypothetical protein |
| XNC1_1384 | 247 | XP8 | 300722361 | Methyltransferase |
| XNC1_1393 | 935 | XP8 | 300722370 | Pullulanase precursor (Alpha-dextrin endo-1,6-alpha-glucosidase) (Pullulan 6-glucanohydrolase) |
| XNC1_1948 | 229 | XP8 | 300722895 | putative transcriptional regulator |
| XNC1_2083 | 506 | XP8 | 300723025 | putative Alkaline phosphatase |
| XNC1_2271 | 257 | XP8 | 300723207 | hypothetical protein |
| XNC1_2445 | 245 | XP8 | 300723366 | hypothetical protein |
| XNC1_2468 | 151 | XP8 | 300723387 | hypothetical protein |
| XNC1_2997 | 199 | XP8 | 300723863 | Chitin-binding protein(CBP21 precursor) |
| XNC1_2998 | 131 | XP8 | 300723864 | hypothetical protein |
| XNC1_3120 | 314 | XP8 | 300723978 | conserved hypothetical protein; putative exported protein |
| XNC1_3324 | 335 | XP8 | 300724177 | Extracellular metalloprotease precursor |
| XNC1_3325 | 350 | XP8 | 300724178 | Extracellular metalloprotease precursor |
| XNC1_3888 | 260 | XP8 | 300724679 | hypothetical protein |
| XNC1_4278 | 199 | XP8 | 300725046 | hypothetical protein |
| XNC1_0020 | 819 | XP9 | 300721109 | Type I restriction-modification enzyme subunit M |
| XNC1_0877 | 461 | XP9 | 300721888 | hypothetical protein |
| XNC1_2243 | 674 | XP9 | 300723179 | putative Tyrosine decarboxylase |
| XNC1_2655 | 222 | XP9 | 300723550 | hypothetical protein |
| XNC1_3030 | 74 | XP9 | 300723891 | conserved hypothetical protein; putative exported protein |
| XNC1_3035 | 264 | XP9 | 300723896 | Na(+)-translocating NADH-quinone reductase subunit C (Na(+)-translocating NQR subunit C) (Na(+)-NQR subunit C) (NQR complex subunit C) (NQR-1 subunit C) |
| XNC1_3036 | 412 | XP9 | 300723897 | Na(+)-translocating NADH-quinone reductase subunit B (Na(+)-translocating NQR subunit B) (Na(+)-NQR subunit B) (NQR complex subunit B) (NQR-1 subunit B) |
| XNC1_3037 | 447 | XP9 | 300723898 | Na(+)-translocating NADH-quinone reductase subunit A (Na(+)-translocating NQR subunit A) (Na(+)-NQR subunit A) (NQR complex subunit A) (NQR-1 subunit A) |
| XNC1_3332 | 446 | XP9 | 300724184 | putative Na+ driven multidrug efflux pump |
| XNC1_0144 | 105 | XP10 | 300721219 | hypothetical protein |
| XNC1_0145 | 113 | XP10 | 300721220 | hypothetical protein |
| XNC1_2344 | 118 | XP10 | 300723277 | hypothetical protein |
| XNC1_2345 | 119 | XP10 | 300723278 | hypothetical protein |
| XNC1_2881 | 94 | XP10 | 300723760 | hypothetical protein |
| XNC1_3129 | 335 | XP11 | 300723987 | putative phage integrase |
| XNC1_3506 | 335 | XP11 | 300724337 | putative phage integrase |
| XNC1_3598 | 335 | XP11 | 300724415 | putative phage integrase |
| XNC1_p0074 | 222 | XP12 | 296491892 | hypothetical protein |
| XNC1_p0093 | 339 | XP12 | 296491909 | transposase |
| XNC1_p0094 | 339 | XP12 | 296491910 | transposase |
| XNC1_p0102 | 342 | XP12 | 296491916 | transposase |
| XNC1_p0145 | 222 | XP12 | 296491957 | hypothetical protein |
| XNC1_0084 | 692 | XP12 | 300721166 | hypothetical protein |
| XNC1_0258 | 243 | XP12 | 300721325 | putative transposase |
| XNC1_0594 | 227 | XP12 | 300721625 | transposase (fragment) |
| XNC1_0599 | 346 | XP12 | 300721630 | transposase |
| XNC1_0600 | 346 | XP12 | 300721631 | transposase |
| XNC1_0833 | 244 | XP12 | 300721848 | Putative transposase (fragment) |
| XNC1_0841 | 278 | XP12 | 300721854 | transposase |
| XNC1_1739 | 318 | XP12 | 300722695 | transposase |
| XNC1_1905 | 348 | XP12 | 300722854 | transposase |
| XNC1_2046 | 277 | XP12 | 300722990 | putative transposase |
| XNC1_2395 | 252 | XP12 | 300723319 | transposase (fragment) |
| XNC1_2610 | 268 | XP12 | 300723507 | putative transposase (fragment) |
| XNC1_2747 | 340 | XP12 | 300723642 | transposase |
| XNC1_3010 | 249 | XP12 | 300723876 | transposase (fragment) |
| XNC1_4226 | 98 | XP12 | 300725000 | hypothetical protein |
| XNC1_4234 | 346 | XP12 | 300725008 | transposase |
| XNC1_4407 | 273 | XP12 | 300725165 | Transposase |
| XNC1_4600 | 414 | XP13 | 300725345 | Putative AMP-dependent synthetase/ligase |
| XNC1_0112 | 349 | XP14 | 300721192 | Myo-inositol 2-dehydrogenase |
| XNC1_0158 | 367 | XP14 | 300721233 | WalW protein |
| XNC1_0159 | 376 | XP14 | 300721234 | Lipopolysaccharide core biosynthesis protein RfaG (Glucosyltransferase I) |
| XNC1_0169 | 392 | XP14 | 300721244 | WalM protein |
| XNC1_0170 | 367 | XP14 | 300721245 | WalN protein |
| XNC1_0172 | 372 | XP14 | 300721247 | WalR protein |
| XNC1_0270 | 403 | XP14 | 300721337 | Argininosuccinate synthase (Citrulline--aspartate ligase) |
| XNC1_0377 | 371 | XP14 | 300721420 | Histone deacetylase-like amidohydrolase (HDAC-like amidohydrolase) (HDAH) |
| XNC1_0682 | 242 | XP14 | 300721705 | putative Phosphonate-transporting ATPase |
| XNC1_1391 | 382 | XP14 | 300722368 | putative Aminotransferase |
| XNC1_2486 | 338 | XP14 | 300723405 | putative epimerase |
| XNC1_2487 | 447 | XP14 | 300723406 | putative UDP-glucose 6-dehydrogenase (Ugd) (Udg) |
| XNC1_2951 | 393 | XP14 | 300723819 | Probable alginate O-acetylation protein AlgI |
| XNC1_3289 | 540 | XP14 | 300724142 | putative glutamine-dependent NAD(+) synthetase (NAD(+) synthase [glutamine-hydrolyzing]) (NadE) |
| XNC1_4601 | 811 | XP14 | 300725346 | putative Long-chain-fatty-acyl-CoA reductase |
| XNC1_0721 | 538 | XP15 | 300721742 | transposase |
| XNC1_3339 | 538 | XP15 | 300724189 | putative transposase |
| XNC1_3829 | 538 | XP15 | 300724625 | transposase |
| XNC1_3832 | 538 | XP15 | 300724628 | transposase |
| XNC1_4479 | 147 | XP15 | 300725237 | hypothetical protein |
