# Supplementary material for: The Entomopathogenic Bacterial Endosymbionts Xenorhabdus and Photorhabdus: Convergent Lifestyles from Divergent Genomes
Source: PLoS One. 2011 Nov 18;6(11):e27909. doi: 10.1371/journal.pone.0027909 (PMC3220699; doi:10.1371/journal.pone.0027909)
Supplement: Table S6 — Gene identities and annotations found within mountains on a phylogenomic map constructed for orthologous genes found between Xenorhabdus nematophila and X. bovienii but not in Photorhabdus luminescens , P. asymbiotica , Salmonella typhimurium LT2, or Escherichia coli K12. (DOC) [file pone.0027909.s008.doc]

**Table S6.** Gene identities and annotations found within mountains on a phylogenomic map constructed for orthologous genes found between *Xenorhabdus nematophila* and *X. bovienii* but not in *Photorhabdus* *luminescens*, *P. asymbiotica*, Salmonella *typhimurium* LT2, or *Escherichia coli* K12.

| **Gene ID** | **Length** | **Mountain** | **Protein ID** | **Annotation** |
| --- | --- | --- | --- | --- |
| XNC1_0557 | 107 | X1 | 300721588 | transposase |
| XNC1_0558 | 136 | X1 | 300721589 | transposase |
| XNC1_0609 | 268 | X1 | 300721640 | putative ABC-2 type transport system permease protein |
| XNC1_0775 | 107 | X1 | 300721790 | transposase |
| XNC1_0776 | 134 | X1 | 300721791 | transposase |
| XNC1_0848 | 136 | X1 | 300721861 | transposase |
| XNC1_0849 | 107 | X1 | 300721862 | transposase |
| XNC1_1476 | 107 | X1 | 300722449 | transposase |
| XNC1_1477 | 136 | X1 | 300722450 | transposase |
| XNC1_1777 | 136 | X1 | 300722733 | transposase |
| XNC1_1801 | 107 | X1 | 300722754 | transposase |
| XNC1_1995 | 136 | X1 | 300722941 | transposase |
| XNC1_2389 | 140 | X1 | 300723313 | hypothetical protein |
| XNC1_2394 | 74 | X1 | 300723318 | transposase (fragment) |
| XNC1_2398 | 107 | X1 | 300723322 | transposase |
| XNC1_2407 | 204 | X1 | 300723330 | transposase |
| XNC1_2938 | 107 | X1 | 300723808 | transposase |
| XNC1_2939 | 136 | X1 | 300723809 | transposase |
| XNC1_3002 | 136 | X1 | 300723868 | transposase |
| XNC1_3117 | 259 | X1 | 300723975 | hypothetical protein |
| XNC1_3421 | 136 | X1 | 300724263 | putative transposase |
| XNC1_3637 | 136 | X1 | 300724448 | transposase |
| XNC1_3638 | 107 | X1 | 300724449 | transposase |
| XNC1_3657 | 107 | X1 | 300724466 | transposase |
| XNC1_4531 | 136 | X1 | 300725284 | transposase |
| XNC1_4532 | 107 | X1 | 300725285 | transposase |
| XNC1_2963 | 192 | X2 | 300723829 | tellurium resistance protein terD |
| XNC1_2965 | 151 | X2 | 300723831 | tellurium resistance protein terB |
| XNC1_2966 | 390 | X2 | 300723832 | tellurium resistance protein terA |
| XNC1_2967 | 195 | X2 | 300723833 | tellurium resistance protein terZ |
| XNC1_2968 | 384 | X2 | 300723834 | hypothetical protein |
| XNC1_2969 | 359 | X2 | 300723835 | hypothetical protein |
| XNC1_2970 | 312 | X2 | 300723836 | hypothetical protein |
| XNC1_p0086 | 237 | X3 | 296491904 | transposase |
| XNC1_0070 | 130 | X3 | 300721152 | transposase (fragment) |
| XNC1_0231 | 231 | X3 | 300721300 | transposase |
| XNC1_0260 | 231 | X3 | 300721327 | transposase |
| XNC1_0695 | 231 | X3 | 300721718 | transposase |
| XNC1_0701 | 231 | X3 | 300721724 | transposase |
| XNC1_0777 | 138 | X3 | 300721792 | transposase (fragment) |
| XNC1_0779 | 87 | X3 | 300721794 | transposase (fragment) |
| XNC1_2170 | 231 | X3 | 300723111 | transposase |
| XNC1_2332 | 65 | X3 | 300723266 | transposase (fragment) |
| XNC1_4446 | 75 | X3 | 300725204 | transposase (fragment) |
| XNC1_4448 | 125 | X3 | 300725206 | transposase (fragment) |
| XNC1_4584 | 231 | X3 | 300725329 | transposase |
| XNC1_4639 | 178 | X3 | 300725381 | transposase (fragment) |
| XNC1_0718 | 318 | X4 | 300721741 | transposase |
| XNC1_0832 | 318 | X4 | 300721847 | transposase |
| XNC1_1899 | 322 | X4 | 300722848 | transposase |
| XNC1_3015 | 318 | X4 | 300723881 | transposase |
| XNC1_p0095 | 181 | X5 | 296491911 | hypothetical protein |
| XNC1_0017 | 244 | X5 | 300721106 | Zinc metalloprotease |
| XNC1_0018 | 1029 | X5 | 300721107 | Type I site-specific deoxyribonuclease HsdR |
| XNC1_0019 | 452 | X5 | 300721108 | Type I restriction-modification enzyme subunit S |
| XNC1_0089 | 259 | X5 | 300721171 | GntR-family transcriptional regulator |
| XNC1_0091 | 485 | X5 | 300721173 | Cytosine/purines, uracil, thiamine, allantoin permease family protein (modular protein) |
| XNC1_0184 | 248 | X5 | 300721259 | hypothetical protein |
| XNC1_0186 | 279 | X5 | 300721261 | hypothetical protein |
| XNC1_0187 | 339 | X5 | 300721262 | putative phage gene |
| XNC1_0189 | 170 | X5 | 300721264 | putative bacteriophage protein |
| XNC1_0190 | 72 | X5 | 300721265 | hypothetical protein |
| XNC1_0431 | 401 | X5 | 300721467 | hypothetical protein; putative membrane protein |
| XNC1_0602 | 377 | X5 | 300721633 | hypothetical protein |
| XNC1_0603 | 137 | X5 | 300721634 | putative Metallothiol transferase fosB (Fosfomycin resistance protein) |
| XNC1_0607 | 291 | X5 | 300721638 | hypothetical protein |
| XNC1_0610 | 260 | X5 | 300721641 | hypothetical protein |
| XNC1_0611 | 223 | X5 | 300721642 | putative Arsenical resistance protein ArsH |
| XNC1_0612 | 381 | X5 | 300721643 | hypothetical protein |
| XNC1_0646 | 1054 | X5 | 300721672 | Putative non-ribosomal peptide synthetase (fragment) |
| XNC1_0732 | 258 | X5 | 300721751 | hypothetical protein |
| XNC1_0745 | 437 | X5 | 300721762 | putative sodium/chloride ion channel |
| XNC1_0859 | 170 | X5 | 300721870 | hypothetical protein |
| XNC1_0867 | 286 | X5 | 300721878 | hypothetical protein; putative exported protein |
| XNC1_0918 | 78 | X5 | 300721927 | hypothetical protein |
| XNC1_1004 | 453 | X5 | 300722011 | Similar to Biotin carboxylase |
| XNC1_1005 | 238 | X5 | 300722012 | hypothetical protein |
| XNC1_1006 | 408 | X5 | 300722013 | Major facilitator family transporter |
| XNC1_1014 | 119 | X5 | 300722021 | Putative phage integrase (fragment) |
| XNC1_1032 | 286 | X5 | 300722036 | hypothetical protein |
| XNC1_1068 | 533 | X5 | 300722068 | putative ATPase component of ABC transporters with duplicated ATPase domains |
| XNC1_1102 | 61 | X5 | 300722100 | hypothetical protein |
| XNC1_1210 | 1036 | X5 | 300722202 | hypothetical protein |
| XNC1_1224 | 209 | X5 | 300722216 | putative glutathione S-transferase |
| XNC1_1236 | 173 | X5 | 300722228 | hypothetical protein; putative exported protein |
| XNC1_1248 | 418 | X5 | 300722240 | hypothetical protein; putative membrane protein |
| XNC1_1439 | 192 | X5 | 300722414 | hypothetical protein |
| XNC1_1467 | 118 | X5 | 300722440 | hypothetical protein |
| XNC1_1468 | 372 | X5 | 300722441 | hypothetical protein |
| XNC1_1475 | 734 | X5 | 300722448 | Non-ribosomal peptide synthetase |
| XNC1_1643 | 526 | X5 | 300722610 |  |
| XNC1_1647 | 72 | X5 | 300722614 | hypothetical protein |
| XNC1_1648 | 218 | X5 | 300722615 | Phenazine biosynthesis PhzC/PhzF protein |
| XNC1_1707 | 383 | X5 | 300722664 | Acyl-CoA dehydrogenase involved in xenocoumacin synthesis |
| XNC1_1708 | 85 | X5 | 300722665 | Putative acyl carrier protein potentially involved in xenocoumacin synthesis |
| XNC1_1711 | 2672 | X5 | 300722668 | Non-ribosomal peptide synthase involved in Xenocoumacin synthesis |
| XNC1_1780 | 1654 | X5 | 300722736 | putative Nematicidal protein 2 |
| XNC1_1950 | 128 | X5 | 300722896 | hypothetical protein |
| XNC1_1963 | 150 | X5 | 300722909 | hypothetical protein |
| XNC1_1964 | 65 | X5 | 300722910 | Regulatory protein cro |
| XNC1_1965 | 237 | X5 | 300722911 | putative prophage repressor protein ( phage repressor protein cI) |
| XNC1_1971 | 190 | X5 | 300722917 | hypothetical protein |
| XNC1_1996 | 249 | X5 | 300722942 | NgrE (modular protein) |
| XNC1_2009 | 68 | X5 | 300722955 | hypothetical protein |
| XNC1_2086 | 189 | X5 | 300723028 | hypothetical protein |
| XNC1_2194 | 361 | X5 | 300723135 | putative lipoprotein precursor |
| XNC1_2202 | 103 | X5 | 300723143 | hypothetical protein |
| XNC1_2244 | 281 | X5 | 300723180 | hypothetical protein |
| XNC1_2245 | 334 | X5 | 300723181 | hypothetical protein |
| XNC1_2257 | 163 | X5 | 300723193 | HTH-type transcriptional regulator pecS |
| XNC1_2376 | 85 | X5 | 300723303 | TraE (fragment) |
| XNC1_2520 | 116 | X5 | 300723436 | Conserved Hypothetical protein (putative lipoprotein of SST VI cluster) |
| XNC1_2528 | 509 | X5 | 300723444 | conserved hypothetical protein (probable component of SST VI cluster) |
| XNC1_2593 | 144 | X5 | 300723491 | Glutathione-dependent formaldehyde-activating, GFA |
| XNC1_2660 | 109 | X5 | 300723555 | hypothetical protein |
| XNC1_2678 | 487 | X5 | 300723573 | putative divalent cation transport protein ( divalent cation transporter) |
| XNC1_2699 | 160 | X5 | 300723594 | conserved hypothetical protein; putative exported protein |
| XNC1_2700 | 123 | X5 | 300723595 | hypothetical protein; putative exported protein |
| XNC1_2701 | 128 | X5 | 300723596 | hypothetical protein |
| XNC1_2702 | 102 | X5 | 300723597 | hypothetical protein |
| XNC1_2722 | 159 | X5 | 300723617 | putative UmoD |
| XNC1_2757 | 70 | X5 | 300723652 | hypothetical protein |
| XNC1_2873 | 74 | X5 | 300723752 | hypothetical protein |
| XNC1_2883 | 69 | X5 | 300723762 | hypothetical protein |
| XNC1_2909 | 118 | X5 | 300723783 | hypothetical protein |
| XNC1_2910 | 405 | X5 | 300723784 | hypothetical protein |
| XNC1_2943 | 202 | X5 | 300723813 | transposase (fragment) |
| XNC1_2960 | 116 | X5 | 300723826 | hypothetical protein |
| XNC1_2971 | 161 | X5 | 300723837 | hypothetical protein |
| XNC1_2974 | 1040 | X5 | 300723840 | Extracellular serine protease precursor |
| XNC1_2991 | 269 | X5 | 300723857 | transposase (fragment) |
| XNC1_2995 | 103 | X5 | 300723861 | transposase (fragment) |
| XNC1_3221 | 165 | X5 | 300724075 | hypothetical protein |
| XNC1_3222 | 106 | X5 | 300724076 | hypothetical protein |
| XNC1_3370 | 85 | X5 | 300724216 | hypothetical protein |
| XNC1_3452 | 69 | X5 | 300724292 | hypothetical protein |
| XNC1_3463 | 125 | X5 | 300724302 | hypothetical protein |
| XNC1_3473 | 114 | X5 | 300724309 | hypothetical protein |
| XNC1_3480 | 64 | X5 | 300724314 | hypothetical protein |
| XNC1_3485 | 86 | X5 | 300724319 | hypothetical protein |
| XNC1_3500 | 184 | X5 | 300724333 | hypothetical protein |
| XNC1_3530 | 129 | X5 | 300724354 | hypothetical protein |
| XNC1_3540 | 69 | X5 | 300724363 | hypothetical protein |
| XNC1_3552 | 230 | X5 | 300724374 | hypothetical protein |
| XNC1_3560 | 118 | X5 | 300724382 | hypothetical protein |
| XNC1_3641 | 129 | X5 | 300724452 | hypothetical protein |
| XNC1_3652 | 69 | X5 | 300724463 | hypothetical protein |
| XNC1_3671 | 230 | X5 | 300724480 | hypothetical protein |
| XNC1_3773 | 212 | X5 | 300724573 | putative transcriptional regulator, TetR family |
| XNC1_3910 | 554 | X5 | 300724701 | Beta-ketoacyl synthase |
| XNC1_4108 | 228 | X5 | 300724894 | hypothetical protein |
| XNC1_4133 | 64 | X5 | 300724909 | hypothetical protein |
| XNC1_4206 | 324 | X5 | 300724982 | putative transposase |
| XNC1_4207 | 62 | X5 | 300724983 | putative transposase (fragment) |
| XNC1_4208 | 410 | X5 | 300724984 | putative alanine racemase |
| XNC1_4236 | 253 | X5 | 300725010 | transposase |
| XNC1_4295 | 164 | X5 | 300725063 | putative transposase |
| XNC1_4405 | 313 | X5 | 300725163 | putative Transposase (TnpA-like protein) |
| XNC1_4528 | 57 | X5 | 300725281 | hypothetical protein |
| XNC1_4609 | 345 | X5 | 300725354 | transposase (fragment) |
| XNC1_p0110 | 323 | X6 | 296491924 | hypothetical protein |
| XNC1_0063 | 45 | X6 | 300721145 | hypothetical protein |
| XNC1_0215 | 63 | X6 | 300721284 | hypothetical protein |
| XNC1_0559 | 57 | X6 | 300721590 | hypothetical protein |
| XNC1_0560 | 104 | X6 | 300721591 | hypothetical protein |
| XNC1_0606 | 298 | X6 | 300721637 | hypothetical protein |
| XNC1_0755 | 47 | X6 | 300721772 | hypothetical protein |
| XNC1_0871 | 122 | X6 | 300721882 | putative Transposase |
| XNC1_1031 | 162 | X6 | 300722035 | hypothetical protein; putative exported protein |
| XNC1_1038 | 132 | X6 | 300722040 | hypothetical protein |
| XNC1_1234 | 70 | X6 | 300722226 | hypothetical protein |
| XNC1_1292 | 60 | X6 | 300722278 | hypothetical protein |
| XNC1_1445 | 49 | X6 | 300722420 | hypothetical protein |
| XNC1_1488 | 51 | X6 | 300722461 | hypothetical protein |
| XNC1_1505 | 48 | X6 | 300722478 | hypothetical protein |
| XNC1_1649 | 172 | X6 | 300722616 | hypothetical protein; putative exported protein |
| XNC1_1656 | 42 | X6 | 300722621 | hypothetical protein |
| XNC1_1721 | 48 | X6 | 300722678 | hypothetical protein |
| XNC1_1813 | 61 | X6 | 300722766 | hypothetical protein |
| XNC1_1848 | 56 | X6 | 300722800 | hypothetical protein; putative exported protein |
| XNC1_1903 | 122 | X6 | 300722852 | hypothetical protein |
| XNC1_1953 | 57 | X6 | 300722899 | hypothetical protein; putative exported protein |
| XNC1_2001 | 52 | X6 | 300722947 | hypothetical protein |
| XNC1_2006 | 145 | X6 | 300722952 | hypothetical protein |
| XNC1_2090 | 122 | X6 | 300723032 | putative transposase (fragment) |
| XNC1_2127 | 211 | X6 | 300723069 | hypothetical protein |
| XNC1_2128 | 63 | X6 | 300723070 | hypothetical protein; putative exported protein |
| XNC1_2225 | 53 | X6 | 300723163 | hypothetical protein |
| XNC1_2440 | 44 | X6 | 300723361 | hypothetical protein |
| XNC1_2545 | 125 | X6 | 300723456 | hypothetical protein |
| XNC1_2574 | 86 | X6 | 300723479 | hypothetical protein |
| XNC1_2576 | 75 | X6 | 300723480 | hypothetical protein |
| XNC1_2603 | 82 | X6 | 300723501 | hypothetical protein |
| XNC1_2794 | 53 | X6 | 300723678 | hypothetical protein |
| XNC1_2879 | 112 | X6 | 300723758 | hypothetical protein |
| XNC1_2882 | 127 | X6 | 300723761 | hypothetical protein |
| XNC1_2884 | 104 | X6 | 300723763 | hypothetical protein |
| XNC1_2921 | 67 | X6 | 300723795 | hypothetical protein |
| XNC1_3098 | 224 | X6 | 300723957 | hypothetical protein |
| XNC1_3137 | 206 | X6 | 300723995 | Putative integrase |
| XNC1_3199 | 122 | X6 | 300724054 | hypothetical protein |
| XNC1_3214 | 57 | X6 | 300724068 | hypothetical protein |
| XNC1_3281 | 53 | X6 | 300724134 | hypothetical protein |
| XNC1_3410 | 139 | X6 | 300724253 | hypothetical protein |
| XNC1_3433 | 62 | X6 | 300724275 | hypothetical protein |
| XNC1_3436 | 38 | X6 | 300724278 | hypothetical protein |
| XNC1_3437 | 57 | X6 | 300724279 | hypothetical protein |
| XNC1_3478 | 139 | X6 | 300724313 | hypothetical protein |
| XNC1_3498 | 149 | X6 | 300724331 | hypothetical protein |
| XNC1_3499 | 153 | X6 | 300724332 | hypothetical protein |
| XNC1_3517 | 114 | X6 | 300724344 | hypothetical protein |
| XNC1_3567 | 263 | X6 | 300724389 | hypothetical protein |
| XNC1_3592 | 99 | X6 | 300724409 | putative antirestriction protein |
| XNC1_3593 | 177 | X6 | 300724410 | hypothetical protein |
| XNC1_3679 | 118 | X6 | 300724488 | hypothetical protein |
| XNC1_3691 | 263 | X6 | 300724498 | hypothetical protein |
| XNC1_3725 | 120 | X6 | 300724528 | hypothetical protein |
| XNC1_3765 | 75 | X6 | 300724566 | hypothetical protein |
| XNC1_3772 | 75 | X6 | 300724572 | putative transposase (fragment) |
| XNC1_3824 | 122 | X6 | 300724620 | Putative transposase (fragment) |
| XNC1_3989 | 39 | X6 | 300724777 | hypothetical protein |
| XNC1_4109 | 68 | X6 | 300724895 | hypothetical protein |
| XNC1_4129 | 34 | X6 | 300724907 | hypothetical protein |
| XNC1_4141 | 451 | X6 | 300724917 | hypothetical protein |
| XNC1_4284 | 47 | X6 | 300725052 | hypothetical protein |
| XNC1_4352 | 47 | X6 | 300725116 | hypothetical protein |
| XNC1_4382 | 58 | X6 | 300725146 | hypothetical protein |
| XNC1_4384 | 95 | X6 | 300725148 | hypothetical protein |
| XNC1_4402 | 49 | X6 | 300725162 | hypothetical protein |
| XNC1_4406 | 127 | X6 | 300725164 | hypothetical protein |
| XNC1_4422 | 399 | X6 | 300725180 | hypothetical protein; putative membrane protein |
| XNC1_4423 | 229 | X6 | 300725181 | hypothetical protein; putative membrane protein |
| XNC1_4424 | 339 | X6 | 300725182 | hypothetical protein |
| XNC1_4440 | 177 | X6 | 300725198 | hypothetical protein |
| XNC1_4468 | 88 | X6 | 300725226 | hypothetical protein |
| XNC1_4470 | 43 | X6 | 300725228 | hypothetical protein |
| XNC1_4483 | 124 | X6 | 300725241 | hypothetical protein; putative exported protein |
| XNC1_4484 | 45 | X6 | 300725242 | hypothetical protein |
| XNC1_4523 | 75 | X6 | 300725278 | hypothetical protein; putative exported protein |
| XNC1_4529 | 196 | X6 | 300725282 | hypothetical protein |
| XNC1_4561 | 51 | X6 | 300725313 | putative Transposase (fragment) |
| XNC1_4587 | 159 | X6 | 300725332 | hypothetical protein; putative exported protein |
| XNC1_4610 | 53 | X6 | 300725355 | hypothetical protein |
| XNC1_p0106 | 126 | X7 | 296491920 | hypothetical protein |
| XNC1_0869 | 58 | X7 | 300721880 | putative Transposase |
| XNC1_1667 | 58 | X7 | 300722625 | hypothetical protein |
| XNC1_1668 | 139 | X7 | 300722626 | putative transposase (fragment) |
| XNC1_1901 | 58 | X7 | 300722850 | hypothetical protein |
| XNC1_1902 | 115 | X7 | 300722851 | hypothetical protein |
| XNC1_2004 | 58 | X7 | 300722950 | putative transposase |
| XNC1_2005 | 115 | X7 | 300722951 | hypothetical protein |
| XNC1_2091 | 115 | X7 | 300723033 | putative transposase (fragment) |
| XNC1_2092 | 58 | X7 | 300723034 | hypothetical protein |
| XNC1_2539 | 85 | X7 | 300723453 | putative transposase (fragment) |
| XNC1_2637 | 58 | X7 | 300723534 | hypothetical protein |
| XNC1_3019 | 82 | X7 | 300723885 | hypothetical protein |
| XNC1_3200 | 115 | X7 | 300724055 | putative transposase (fragment) |
| XNC1_3201 | 58 | X7 | 300724056 | putative transposase (fragment) |
| XNC1_3825 | 115 | X7 | 300724621 | hypothetical protein |
| XNC1_4383 | 115 | X7 | 300725147 | putative transposase |
| XNC1_0580 | 210 | X8 | 300721611 | Aec51 |
| XNC1_1010 | 176 | X8 | 300722017 | putative transposase |
| XNC1_2012 | 210 | X8 | 300722956 | Aec51 |
| XNC1_2126 | 399 | X8 | 300723068 | hypothetical protein (Similarities with unknown protein from a prophage) |
| XNC1_2312 | 210 | X8 | 300723246 | hypothetical protein |
| XNC1_2348 | 210 | X8 | 300723281 | putative transposase |
| XNC1_2928 | 210 | X8 | 300723800 | transposase |
| XNC1_3135 | 210 | X8 | 300723993 | putative transposase |
| XNC1_3393 | 466 | X8 | 300724237 | putative DNA circulation protein |
| XNC1_3394 | 362 | X8 | 300724238 | putative tail protein |
| XNC1_3395 | 181 | X8 | 300724239 | putative baseplate assembly protein |
| XNC1_3396 | 149 | X8 | 300724240 | putative bacteriophage protein |
| XNC1_3398 | 193 | X8 | 300724242 | putative prophage protein |
| XNC1_4270 | 205 | X8 | 300725038 | Putative transposase |
| XNC1_0857 | 72 | X9 | 300721868 | Site-specific recombinase, phage integrase family (fragment) |
| XNC1_3425 | 553 | X9 | 300724267 | putative terminase large subunit |
| XNC1_3426 | 117 | X9 | 300724268 | hypothetical protein |
| XNC1_3427 | 144 | X9 | 300724269 | Putative holin protein |
| XNC1_3428 | 100 | X9 | 300724270 | hypothetical protein |
| XNC1_3429 | 91 | X9 | 300724271 | Putative head-tail adaptor |
| XNC1_3965 | 91 | X9 | 300724756 | Putative head-tail adaptor (fragment) |
| XNC1_3966 | 100 | X9 | 300724757 | hypothetical protein |
| XNC1_3967 | 144 | X9 | 300724758 | hypothetical protein |
| XNC1_3969 | 119 | X9 | 300724760 | hypothetical protein |
| XNC1_3970 | 553 | X9 | 300724761 | putative terminase large subunit |
| XNC1_4100 | 553 | X9 | 300724886 | putative terminase large subunit |
| XNC1_4101 | 117 | X9 | 300724887 | hypothetical protein |
| XNC1_4102 | 142 | X9 | 300724888 | hypothetical protein |
| XNC1_4103 | 98 | X9 | 300724889 | hypothetical protein |
| XNC1_4104 | 112 | X9 | 300724890 | putative head-tail adaptor |
