# Supplementary material for: The Entomopathogenic Bacterial Endosymbionts Xenorhabdus and Photorhabdus: Convergent Lifestyles from Divergent Genomes
Source: PLoS One. 2011 Nov 18;6(11):e27909. doi: 10.1371/journal.pone.0027909 (PMC3220699; doi:10.1371/journal.pone.0027909)
Supplement: Text S1 — General Metabolism. (DOC) [file pone.0027909.s009.doc]

**Text S1: General Metabolism**

John M. Chaston^1^, Garret Suen^1^, Kelsea A. Jewell^1^, Xiaojun Lu^1^, Cathy Wheeler^2^, Brad Goodner^2^ and Heidi Goodrich-Blair^1^

^1^Department of Bacteriology, University of Wisconsin-Madison, Madison, Wisconsin, United States of America

^2^Department of Biology, Hiram College, Hiram, Ohio, United States of America

E-mail: hgblair@bact.wisc.edu

Presence or absence of *X. nematophila* and *X. bovienii* metabolic pathways, as determined by the Kyoto Encyclopedia of Genes and Genomes (KEGG) [1] is reported in Table 1. In general, both species are able to perform a variety of respiratory metabolic pathways (glycolysis, etc.) and synthesize most of their own amino acids. This is consistent with both bacteria being facultative anaerobes, with free-living, host-associated, and pathogenic stages in their life cycles. In addition to a shared pyridine biosynthesis deficiency, we noticed several pathways were present in only *X. nematophila* or *X. bovienii*, and relate their presence or absence to possible growth and virulence phenotypes below.

Although both *X. nematophila* and *X. bovienii* encode complete pathways for amino acid biosynthesis, neither has *nadA, nadB,* or *nadC* genes encoding enzymes necessary for synthesis of pyridine (NAD). Consistent with this, *X. nematophila* requires nicotinate supplementation for growth in minimal medium [2]. In contrast, both *P. luminescens* and *P. asymbiotica* encode *nadA, nadB,* and *nadC*. Since host, but not soil or water, environments are likely sources of pyridine, it has been suggested that microbes that lack an environmental phase have less selective pressure to maintain pyridine prototrophy [3]. Our data suggest that *Xenorhabdus* spp., but not *Photorhabdus* spp. are restricted for growth outside their animal hosts.

*X. nematophila*, but not *X. bovienii*, contains a putative cellobiose transport system. Many *Xenhorhabdus* spp. can produce acid when grown on cellobiose as a sole carbon source [4], but *X. nematophila* and *X. bovienii* both lack cellulases, suggesting that this carbon source may be used opportunistically rather than as part of a cellulolytic consortium. For example, cellobiose uptake and fermentation may allow *X. nematophila* to take advantage of partially digested cellulosic material in the insect cadaver or gut of host nematodes.

*Xenorhabdus* are classified as facultative anaerobes and are expected to encode enzymes required for anaerobic respiration and/or fermentation. Neither *X. nematophila* nor *X. bovienii* encodes nitrate reductase, indicating these bacteria do not utilize the anaerobic electron acceptor preferred by *Escherichia coli*. However, both *Xenorhabdus* spp. encode fumarate reductase that should allow fumarate to serve as an electron acceptor [5]. In *E. coli* NarL and NarP transcription factors regulate of nitrate and fumarate reductase gene expression, with NarL activated at high, and NarP at low, nitrate conditions [6]. NarP activates expression of nitrate reductase, while NarL both activates one nitrate reductase pathway and suppresses a second, in addition to suppressing expression of fumarate reductase [6]. *X. bovienii* lacks NarP, consistent with the absence of nitrate reductase. It is likely that NarP/L homologs present in *Xenorhabdus* have evolved distinct regulatory functions.

*X. bovienii*, but not *X. nematophila*, is predicted to encode the YfhKA two component regulatory system. The homologous system in *E. coli* (QseEF) is necessary for fine-tuning the formation of effacing lesions on intestinal epithelial cells after attachment [7]. YfhKA may be similarly involved in *X. bovienii* virulence in insects. The two-component regulatory systems CpxRA [8,9,10] and OmpR/EnvZ [11,12,13,14] are involved in regulation of both mutualism and pathogenesis factors in *X. nematophila*. It is not known if these systems are necessary for *X. bovienii* mutualism and pathogenesis, or if other regulatory systems, such as YhfKA, may perform analogous roles.

In summary, both *X. nematophila* and *X. bovienii* have metabolic profiles as expected for bacteria capable of being free living, host-associated, and pathogenic by turns. However, there are several unique pathways to each species, including cellobiose transport and presence of a putative virulence-linked YfhKA two component system. Some differences in pathway constitution, such as the absence of NarP in the *X. bovienii* fumarate reductase pathway, do not have obvious effects on the expected phenotype (i.e. anaerobic respiration), suggesting that the function for absent genes may be substituted for by analogous genes or pathways. It is also possible that some pathway elements have been lost due to nutrients available to *Xenorhabdus* spp. through host-association with nematodes, as is the case for pyridine biosynthesis.

**References**

1. Kanehisa M, Araki M, Goto S, Hattori M, Hirakawa M, et al. (2008) KEGG for linking genomes to life and the environment. Nucleic Acids Res 36: D480-484.

2. Orchard SS, Goodrich-Blair H (2004) Identification and functional characterization of a *Xenorhabdus nematophila* oligopeptide permease. Appl Environ Microbiol 70: 5621-5627.

3. Bergthorsson U, Roth JR (2005) Natural isolates of *Salmonella enterica* serovar Dublin carry a single *nadA* missense mutation. J Bacteriol 187: 400-403.

4. Akhurst RJ (1983) Taxonomic study of *Xenorhabdus*, a genus of bacteria symbiotically associated with insect pathogenic nematodes. Int J Syst Bacteriol 33: 38-45.

5. Iverson TM, Luna-Chavez C, Cecchini G, Rees DC (1999) Structure of the *Escherichia coli* fumarate reductase respiratory complex. Science 284: 1961-1966.

6. Jones SA, Chowdhury FZ, Fabich AJ, Anderson A, Schreiner DM, et al. (2007) Respiration of *Escherichia coli* in the mouse intestine. Infect Immun 75: 4891-4899.

7. Reading NC, Torres AG, Kendall MM, Hughes DT, Yamamoto K, et al. (2007) A novel two-component signaling system that activates transcription of an enterohemorrhagic *Escherichia coli* effector involved in remodeling of host actin. J Bacteriol 189: 2468-2476.

8. Herbert EE, Cowles KN, Goodrich-Blair H (2007) CpxRA regulates mutualism and pathogenesis in *Xenorhabdus nematophila*. Appl Environ Microbiol 73: 7826-7836.

9. Herbert Tran EE, Andersen AW, Goodrich-Blair H (2009) CpxRA influences *Xenorhabdus nematophila* colonization initiation and outgrowth in *Steinernema carpocapsae* nematodes through regulation of the nil locus. Appl Environ Microbiol 75: 4007-4014.

10. Herbert Tran EE, Goodrich-Blair H (2009) CpxRA contributes to *Xenorhabdus nematophila* virulence through regulation of *lrhA* and modulation of insect immunity. Appl Environ Microbiol 75: 3998-4006.

11. Forst S, Boylan B (2002) Characterization of the pleiotropic phenotype of an *ompR* strain of *Xenorhabdus nematophila*. Antonie Van Leeuwenhoek 81: 43-49.

12. Kim DJ, Boylan B, George N, Forst S (2003) Inactivation of *ompR* promotes precocious swarming and *flhDC* expression in *Xenorhabdus nematophila*. J Bacteriol 185: 5290-5294.

13. Park D, Forst S (2006) Co-regulation of motility, exoenzyme and antibiotic production by the EnvZ-OmpR-FlhDC-FliA pathway in *Xenorhabdus nematophila*. Mol Microbiol 61: 1397-1412.

14. Tabatabai N, Forst S (1995) Molecular analysis of the two-component genes, *ompR* and *envZ*, in the symbiotic bacterium *Xenorhabdus nematophilus*. Mol Microbiol 17: 643-652.

**Table 1. Metabolic pathways present in *X. nematophila* and *X. bovienii*.**

|  | ***X. nematophila*** | ***X. bovienii*** |
| --- | --- | --- |
| **Metabolism** | | |
| **Carbohydrate metabolism** | | |
| *Glycolysis/Gluconeogenesis* | Yes | Yes |
| *TCA* | Yes | Yes |
| *Pentose Phosphate* | Yes | Yes |
| *Entner Duoderoff* | No | No |
| **Energy metabolism** | | |
| *Oxidative phosphorylation* | | |
| NADH Dehydrogenase | Yes | Yes |
| Succinate dehydrogenase | Yes | Yes |
| Cytochrome c oxidase | Yes | Yes |
| Cytochrome c reductase | No | No |
| Cytochrome c oxidase, cbb-3-type | No | No |
| Cytochrome bd complex | Yes | Yes |
| F-type ATPase | Yes | Yes |
| *Methane metabolism* | No | No |
| *Nitrogen metabolism* | No | No |
| *Sulfur metabolism* | No | No |
| **Lipid metabolism** | | |
| *Fatty acid biosynthesis* | Yes | Yes |
| *Fatty acid metabolism* | Yes | Yes |
| **Nucleotide metabolism** | | |
| *Purine metabolism* | Yes | Yes |
| *Pyrimidine metabolism* | Yes | Yes |
| **Amino acid metabolism** | | |
| *Alanine, aspartate, glutamate metabolism* | Yes | Yes |
| *Glycine, serine, threonine metabolism* | Yes | Yes |
| *Cysteine, methionine metabolism* | Yes | Yes |
| *Valine, leucine, isoleucine biosynthesis* | Yes | Yes |
| *Lysine biosynthesis* | Yes | No |
| *Arginine, proline metabolism* | Yes | Yes |
| *Histidine metabolism* | Yes | Yes |
| *Tyrosine metabolism* | No | No |
| *Phenylalanine metabolism* | No | No |
| *Tryptophan metabolism* | No | No |
| *Phenylalanine, tyrosine, and tryptophan biosynthesis* | Yes | Yes |
| **Metabolism of cofactors and vitamins** | | |
| *Thiamine* | Yes | Yes |
| *Riboflavin* | Yes | Yes |
| *B6* | Yes | Yes |
| *Nicotinic acid / nicotinimate* | No | No |
| *Pathothenate / coA* | Yes | Yes |
| *Biotin* | No | No |
| *Folate* | Yes | Yes |
| *B12* | Yes | Yes |
| *Chlorophyll and porphyrin* | No | No |
| *Ubiquinone* | Yes | Yes |
| *Other terpenes* | No | No |
| **Environmental Information Processing** | | |
| **Membrane Transport** | | |
| *ABC transporters* | | |
| Sulfate | Yes | Yes |
| Molybdate | Yes | Yes |
| Iron (III) | *No* | Yes |
| Thiamin | Yes | Yes |
| Spermidine/putrescine | Yes | Yes |
| Glutamate/aspartate | Yes | Yes |
| Arginine | Yes | Yes |
| Phosphate | Yes | Yes |
| Antibiotics | Yes | Yes |
| Lipoprotein | Yes | Yes |
| Cell division | Yes | Yes |
| Lipopolysaccharide | Yes | Yes |
| General l amino acids | 1 of 4 | 0 of 4 |
| Branched chain amino acid | Yes | *No* |
| Glycine/betaine | *No* | Yes |
| Maltose/maltodextrin | Yes | Yes |
| D-methionine | Yes | Yes |
| Dipeptide/heme/aminolevulinic acid | Yes | Yes |
| Peptide/nickel | Yes | Yes |
| Iron complex | Yes | Yes |
| B12 | Yes | Yes |
| Zinc | Yes | Yes |
| Ribose | *No* | Yes |
| Autoinducer | 1 of 4 | 1 of 4 |
| Iron (II) manganese | Yes | Yes |
| Cobalt | Yes | Yes |
| Nickel | 3 of 4 | 3 of 4 |
| Pyoverdine | *No* | Yes |
| *Phosphotransferase systems* | | |
| Trehalose | Yes | Yes |
| N-acetyl muramic acid | Yes | Yes |
| Mannose | Yes | Yes |
| L-ascorbate | Yes | *No* |
| Cellobiose | *No* | Yes |
| Nitrogen regulation | Yes | Yes |
| N-acetyl-D-glucosamine | Yes | Yes |
| **Signal Transduction** | | |
| *Two component* | |  |
| Phosphate limitation 🡪 Phosphate assimilation | Yes, no endpoint response | Yes, no endpoint response |
| Mg2+ starvation/Antimicrobial peptide 🡪 Virulence, AMP resistance | Yes | Yes |
| Osmotic upshift (K+) | Yes, no endpoint response | Yes, no endpoint response |
| Misfolded proteins | Yes | Yes |
| Secretion stress/Misfolded proteins 🡪 Multidrug efflux | No membrane component | Yes |
| Cell density 🡪 flagella regulon | Downstream only | Downstream only |
| Turgor pressure low 🡪 potassium (K) transport | Yes, but missing one of 4 complex members for K transport | Yes, but missing one of 4 complex members for K transport |
| Redox state of the quinone pool 🡪 anaerobic respiration | Yes | Yes |
| Catabolite repression 🡪 tricarboxylates transport | Yes, but only one of three members of the downstream complex | Yes, but only one of three members of the downstream complex |
| Glucose 6-P 🡪 hexose phosphate uptake | Yes | Yes |
| Glucose 🡪 capsular polysaccharide synthesis | Yes | Yes |
| Low nitrogen availability 🡪 nitrogen assimilation | Yes | Yes |
| YfhK 🡪 YfhA | No | Yes |
| Nitrate/ nitrite 🡪 fumarate reductase | No: NarP only and the full fumarate reductase pathway | Yes: NarX and NarP, and the full fumarate reductase pathway |
