# Supplementary material for: The Entomopathogenic Bacterial Endosymbionts Xenorhabdus and Photorhabdus: Convergent Lifestyles from Divergent Genomes
Source: PLoS One. 2011 Nov 18;6(11):e27909. doi: 10.1371/journal.pone.0027909 (PMC3220699; doi:10.1371/journal.pone.0027909)
Supplement: Text S2 — Transposases. (DOC) [file pone.0027909.s010.doc]

**Text S2: Transposases**

Nydia Morales Soto and Steven Forst

Department of Biological Sciences, University of Wisconsin-Milwaukee, Milwaukee, Wisconsin, United States of America

E-mail: sforst@uwm.edu

*X. nematophila* and *X. bovienii* both contain relatively large numbers of transposases (4.9% and 3.6% of each genome, respectively), which made genome sequence assembly difficult . Both genomes also contain prophage and phage remnants. *X. nematophila* contains two P2-related prophage remnant loci, a Mu prophage and several phage fragments containing phi-31 related genes. Most of the phage genes are located between 3.0 and 4.0 Mb and represent 2.4% of the *X. nematophila* genome. *X. bovienii* contains one P2-related prophage and numerous other prophage fragments but unlike *X. nematophila*, lacks a Mu prophage locus. In *X. bovienii,* most of the phage genes, which represent 3.4% of the genome, are located between 0.6 and 2.0 Mb. Thus, the P2-related phage is the only phage locus that is conserved in these *Xenorhabdus* species. P2-type phage tail structures have been purified from cultures of *X. nematophila* and possess bacteriocin activity against closely related species . Each of the P2 prophage loci in *X. nematophila* contains two separate tail assembly regions but lacks capsid-synthesis, lysis, lysogeny, replication, and late-control genes. Bacteriocin activity has not yet been studied in *X. bovienii*.

**References**

1. Latreille P, Norton S, Goldman BS, Henkhaus J, Miller N, et al. (2007) Optical mapping as a routine tool for bacterial genome sequence finishing. BMC Genomics 8: 321.

2. Thaler JO, Baghdiguian S, Boemare N (1995) Purification and characterization of xenorhabdicin, a phage tail-like bacteriocin, from the lysogenic strain F1 of *Xenorhabdus nematophilus*. Appl Environ Microbiol 61: 2049-2052.
