# Supplementary material for: The Entomopathogenic Bacterial Endosymbionts Xenorhabdus and Photorhabdus: Convergent Lifestyles from Divergent Genomes
Source: PLoS One. 2011 Nov 18;6(11):e27909. doi: 10.1371/journal.pone.0027909 (PMC3220699; doi:10.1371/journal.pone.0027909)
Supplement: Text S3 — Secretion Systems. (DOC) [file pone.0027909.s011.doc]

**Text S3: Secretion Systems**

John M. Chaston^1^, Kimberly N. Cowles^1^, Creg Darby^2^, Kevin Drace^2^, Steven Forst^3^, Barry S. Goldman^4^ and Heidi Goodrich-Blair^1^

^1^Department of Bacteriology, University of Wisconsin-Madison, Madison, Wisconsin, United States of America

^2^Department of Cell and Tissue Biology, University of California, San Francisco, California, United States of America

^3^Department of Biological Sciences, University of Wisconsin-Milwaukee, Milwaukee, Wisconsin, United States of America

^4^Monsanto Company, St. Louis, Missouri, United States of America

E-mail: hgblair@bact.wisc.edu

Symbiotic microorganisms can influence host physiology and gene expression using proteins that are surface localized or secreted, either into the extracellular milieu or directly into host cells. Since *Xenorhabdus* spp. interact with two distinct animal hosts, and appear not to have a free-living life cycle stage, we examined their secretion potential (Table 1, below). Bacterial secretion systems are categorized as 6 types. Type II and V secretion systems utilize N-terminal signal sequences that direct proteins across the cytoplasmic membrane through the Sec secretion system. Signal sequence predictions (0.85 probability greater than 15 aa) for proteins that would utilize the Sec system to cross the membrane reveal 381 proteins in *X. bovienii* and 373 proteins in *X. nematophila*. Of those proteins that are unique to *X. bovienii* or *X. nematophila* (relative to each other, *Y. pestis, P. luminescens,* and *E. coli*) there are 44 and 42 proteins, respectively. The latter includes NilB and NilC, which are known to be *X. nematophila* outer membrane specificity determinants required for colonization of *S. carpocapsae* nematodes.

Type III and IV secretion systems, which transport proteins directly from the bacterium into the host cell, are utilized by both pathogens and mutualists to communicate with and manipulate hosts [1,2], but are lacking in *Xenorhabdus* spp. *P. luminescens* encodes a type III secretion system (T3SS), and the YopT effector was shown to play a role in its anti-phagocytic ability in insects [3]. The *Yersinia* genus includes three species that are pathogenic to humans and other mammals: *Y. pseudotuberculosis* and *Y. enterocolitica*, which cause gastroenteritis, and *Y. pestis,* the agent of bubonic and pneumonic plague. Each of these three contains a virulence plasmid encoding both Yop proteins that disrupt host immune functions and a T3SS that translocates the Yops directly into the cytoplasm of host cells. Furthermore, the T3SS system of *Y. pestis* is responsible for secretion of its insecticidal-toxin-like proteins [4], similar to members of the Tc toxin family discovered in *Photorhabdus* and *Xenorhabdus* [5]. Given the important role of the T3SS in governing insect pathogenic functions in these bacteria, it is surprising that neither *Xenorhabdus* spp. appears to encode this system [3]. Also, there are no Yops encoded in the *Xenorhabdus* sp. genomes, consistent with the fact that these bacteria are not pathogens of mammals. The flagellar secretion system, which is evolutionarily related to the T3SS [6] may be responsible for secretion of virulence determinants. Indeed, XlpA lipase is secreted through the flagellar export apparatus [7]. Further, *X. nematophila* motility mutants lacking flagellar secretion components display a virulence defect, while a mutant lacking only the flagellin subunit does not [7].

*X. nematophila* lacks Type V secretory adhesin genes, whereas *X. bovienii* contains 2, relative to 11 found in *Y. pestis*. Finally, *Xenorhabdus* contains significantly fewer ABC transport proteins relative to other enteric bacteria.  *X. nematophila* and *X. bovienii* encode 18 and 20 ABC-transporters, or efflux pumps, and 5 and 6 biosynthesis gene clusters include such genes, respectively. For comparison, *E. coli* harbours about 80 ABC transporters [8]).

Type VI secretion systems (T6SS) were only recently classified as such, and are known to play a role in virulence in several pathogens including *V. cholerae, L. pneumophila,* and *P. aeruginosa* (reviewed in [9]) and negatively influence mutualistic nodulation of pea by *R. leguminosarum* [10]. Both *Xenorhabdus* spp. encode two T6SS secretion loci, one of Class A and the other of Class D [9], as well as homologs of the T6SS secreted putative effectors Hcp and VgrG. However, deletion of one (Class D) of the two *X. nematophila* T6SS system loci did not influence virulence toward *M. sexta* insects or colonization of *S. carpocapsae* nematodes (K. Cowles and H. Goodrich-Blair, unpublished). This may indicate that the two T6SS systems are functionally redundant, or that the Class D T6SS system is necessary for interaction with a different insect host. Both *P. luminescens* and *P. asymbiotica* encode the class D T6SS, while *P. asymbiotica* encodes all but one factor in the Class A T6SS system and *P. luminescens* encodes only 8 of the 17 genes present in the Class A system of the other three species (Table 2, below). In *P. luminescens* class A remnants are generally located near each other on the chromosome, perhaps suggesting that both islands were present in an ancestor and gradually lost in *P. luminescens*.

Secretion of surface components may also contribute to host interaction phenotypes of *Xenorhabdus* spp. Both *Xenorhabdus* genomes encode homologs of the *Y. pestis* polysaccharide biosynthetic operon *hmsHFRS* [11,12]. *Y. pestis* colonizes fleas and uses them as a vector. *Y. pestis* makes a biofilm in the digestive tract of its vector, the flea; by colonizing the insect in this mode, it is able to resist voiding by peristalsis and defecation [13]. *hmsHFRS* is required for the *in* *vivo* biofilm [11,12]. Furthermore, *Y. pestis* and *Y. pseudotuberculosis* both make an *hmsHFRS-*dependent biofilm that persists tenaciously on the exterior surface of the model nematode *C. elegans* [14,15].

The *X. nematophila* *hmsHFRS* operon identities to *Y. pestis* proteins are: HmsH, 46%; HmsF, 65%; HmsR, 67%; and HmsS, 41%). Like *Yersinia* sp., *X. nematophila* can make biofilms on the head of *C. elegans* [16,17]. Because of this interaction with another nematode, we hypothesized that the *X. nematophila* biofilm is involved in symbiotic colonization of its *S. carpocapsae* host. However, deletion of *hmsR* and *hmsS* produced no defect in either initial colonization or persistence in IJs. These mutants, introduced as either free bacteria injected into insect hemocoel or as symbionts of IJs, were also not attenuated for pathogenicity against *G. mellonella* nor *M. sexta* larvae [17].

It is unusual to have pathogenic bacteria lacking in major virulence secretion systems such as Tat (of which parts exist in *X. nematophila* and *X. bovienii*)[18], and the Type II and III secretion systems [19,20]. It is possible that, in addition to the flagellar export apparatus, the Type Vb system present in these bacteria is playing a role in exporting extracellular virulence-related proteins [21].

**References**

1. Dale C, Plague GR, Wang B, Ochman H, Moran NA (2002) Type III secretion systems and the evolution of mutualistic endosymbiosis. Proc Natl Acad Sci U S A 99: 12397-12402.

2. Galan JE, Wolf-Watz H (2006) Protein delivery into eukaryotic cells by type III secretion machines. Nature 444: 567-573.

3. Brugirard-Ricaud K, Duchaud E, Givaudan A, Girard PA, Kunst F, et al. (2005) Site-specific antiphagocytic function of the Photorhabdus luminescens type III secretion system during insect colonization. Cell Microbiol 7: 363-371.

4. Gendlina I, Held KG, Bartra SS, Gallis BM, Doneanu CE, et al. (2007) Identification and type III-dependent secretion of the Yersinia pestis insecticidal-like proteins. Mol Microbiol 64: 1214-1227.

5. ffrench-Constant R, Waterfield N (2006) An ABC guide to the bacterial toxin complexes. Advances in Applied Microbiology 58: 169-183.

6. Aizawa SI (2001) Bacterial flagella and type III secretion systems. FEMS Microbiol Lett 202: 157-164.

7. Richards GR, Herbert EE, Park Y, Goodrich-Blair H (2008) Xenorhabdus nematophila lrhA is necessary for motility, lipase activity, toxin expression, and virulence in Manduca sexta insects. J Bacteriol 190: 4870-4879.

8. Linton KJ, Higgins CF (1998) The Escherichia coli ATP-binding cassette (ABC) proteins. Mol Microbiol 28: 5-13.

9. Bingle LE, Bailey CM, Pallen MJ (2008) Type VI secretion: a beginner's guide. Curr Opin Microbiol 11: 3-8.

10. Roest HP, Mulders IH, Spaink HP, Wijffelman CA, Lugtenberg BJ (1997) A Rhizobium leguminosarum biovar trifolii locus not localized on the sym plasmid hinders effective nodulation on plants of the pea cross-inoculation group. Mol Plant Microbe Interact 10: 938-941.

11. Hinnebusch BJ, Perry RD, Schwan TG (1996) Role of the Yersinia pestis hemin storage (hms) locus in the transmission of plague by fleas. Science 273: 367-370.

12. Jarrett CO, Deak E, Isherwood KE, Oyston PC, Fischer ER, et al. (2004) Transmission of Yersinia pestis from an infectious biofilm in the flea vector. J Infect Dis 190: 783-792.

13. Hinnebusch BJ (2005) The evolution of flea-borne transmission in Yersinia pestis. Curr Issues Mol Biol 7: 197-212.

14. Darby C, Hsu JW, Ghori N, Falkow S (2002) Caenorhabditis elegans: plague bacteria biofilm blocks food intake. Nature 417: 243-244.

15. Darby C, Ananth SL, Tan L, Hinnebusch BJ (2005) Identification of gmhA, a Yersinia pestis gene required for flea blockage, by using a Caenorhabditis elegans biofilm system. Infect Immun 73: 7236-7242.

16. Couillault C, Ewbank JJ (2002) Diverse bacteria are pathogens of Caenorhabditis elegans. Infect Immun 70: 4705-4707.

17. Drace K, Darby C (2008) The hmsHFRS operon of Xenorhabdus nematophila is required for biofilm attachment to Caenorhabditis elegans. Appl Environ Microbiol 74: 4509-4515.

18. Yuan J, Zweers JC, van Dijl JM, Dalbey RE (2010) Protein transport across and into cell membranes in bacteria and archaea. Cell Mol Life Sci 67: 179-199.

19. Mecsas JJ, Strauss EJ (1996) Molecular mechanisms of bacterial virulence: type III secretion and pathogenicity islands. Emerg Infect Dis 2: 270-288.

20. Sandkvist M (2001) Type II secretion and pathogenesis. Infect Immun 69: 3523-3535.

21. Desvaux M, Parham NJ, Henderson IR (2004) Type V protein secretion: simplicity gone awry? Curr Issues Mol Biol 6: 111-124.

22. Khandelwal P, Banerjee-Bhatnagar N (2003) Insecticidal activity associated with outer membrane vesicles of *Xenorhabdus nematophilus*. Applied and Environmental Microbiology 69: 2032-2037.

23. Sergeant M, Baxter L, Jarrett P, Shaw E, Ousley M, et al. (2006) Identification, typing, and insecticidal activity of *Xenorhabdus* isolates from entomopathogenic nematodes in United Kingdom soil and characterization of the *xpt* toxin loci. Applied and Environmental Microbiology 72: 5895-5907.

24. Waterfield N, Kamita SG, Hammock BD, ffrench-Constant R (2005) The *Photorhabdus* Pir toxins are similar to a developmentally regulated insect protein but show no juvenile hormone esterase activity. FEMS Microbiology Letters 245: 47-52.

25. Pardo-Lopez L, Munoz-Garay C, Porta H, Rodriguez-Almazan C, Soberon M, et al. (2009) Strategies to improve the insecticidal activity of Cry toxins from Bacillus thuringiensis. Peptides 30: 589-595.

26. Wilkinson P, Waterfield NR, Crossman L, Corton C, Sanchez-Contreras M, et al. (2009) Comparative genomics of the emerging human pathogen Photorhabdus asymbiotica with the insect pathogen Photorhabdus luminescens. BMC Genomics 10: 302.

27. Iverson KL, Bromel MC, Anderson AW, Freeman TP (1984) Bacterial symbionts in the sugar beet root maggot *Tetanops myopaeformis* (von Roder). Applied and Environmental Microbiology 47: 22-27.

28. Waterfield N, Bowen DJ, Fetherston JD, Perry RD, ffrench-Constant RH (2001) The toxin complex genes of *Photorhabdus*: a growing gene family. Trends in Microbiology 9: 185-191.

29. Brüssow H, Canchay C, Hardt W-D (2004) Phages and the evolution of bacterial pathogens: from genomic rearrangements to lysogenic conversion. Microbiology and Molecular Biology Reviews 68: 560-602.

30. Yang G, Dowling AJ, Gerike U, ffrench-Constant RH, Waterfield NR (2006) *Photorhabdus* virulence cassettes confer injectable insecticidal activity against the wax moth. Journal of Bacteriology 188: 2254-2261.

31. Turlin E, Pascal G, Rousselle J-C, Lenormand P, Ngo S, et al. (2006) Proteome analysis of the phenotypic variation process in *Photorhabdus luminescens*. Proteomics.

32. Schaumburg J, Diekmann O, Hagendorff P, Bergmann S, Rohde M, et al. (2004) The cell wall subproteome of *Listeria monocytogenes*. Proteomics 4: 2991-3006.

33. Huang LJ, Chen SX, Huang Y, Luo WJ, Jiang HH, et al. (2006) Proteomics-based identification of secreted protein dihydrodiol dehydrogenase as a novel serum markers of non-small cell lung cancer. Lung Cancer 54: 87-94.

34. Barbosa MS, Bao SN, Andreotti PF, de Faria FP, Felipe MS, et al. (2006) Glyceraldehyde-3-phosphate dehydrogenase of *Paracoccidioides brasiliensis* is a cell surface protein involved in fungal adhesion to extracellular matrix proteins and interactions with cells. Infection and Immunity 74: 382-389.

35. Terao Y, Yamaguchi M, Hamada S, Kawabata S (2006) Multifunctional glyceraldehyde-3-phosphate dehydrogenase of *Sterptococcus pyogenes* is essential for evasion from neutrophils. The Journal of Biological Chemistry 281.

36. Raje CI, Kumar S, Harle A, Nanda JS, Raje M (2007) The macrophage cell surface glyceraldehyde-3-phosphate dehydrogenase is a novel transferrin receptor. Journal of Biological Chemistry 282: 3252-3261.

37. Sicard M, Brugirard-Ricaud K, Pages S, Lanois A, Boemare NE, et al. (2004) Stages of infection during the tripartite interaction between *Xenorhabdus nematophila*, its nematode vector, and insect hosts. Applied and Environmental Microbiology 70: 6473-6480.

38. Nandakumar MP, Cheung A, Marten MR (2006) Proteomic Analysis of Extracellular Proteins from Escherichia coli W3110. Journal of Proteome Research 5: 1155-1161.

39. Målen H, Berven FS, Fladmark KE, Wiker HG (2007) Comprehensive analysis of exported proteins from Mycobacterium tuberculosis H37Rv. Proteomics 7: 1702-1718.

40. Turlin E, Pascal G, Rousselle JC, Lenormand P, Ngo S, et al. (2006) Proteome analysis of the phenotypic variation process in Photorhabdus luminescens. Proteomics 6: 2705-2725.

41. Seul KJ, Park SH, Ryu CM, Lee YH, Ghim SY (2007) Proteome analysis of Paenibacillus polymyxa E681 affected by barley. J Microbiol Biotechnol 17: 934-944.

42. Mattinen L, Nissinen R, Riipi T, Kalkkinen N, Pirhonen M (2007) Host-extract induced changes in the secretome of the plant pathogenic bacterium Pectobacterium atrosepticum. Proteomics 7: 3527-3537.

43. Schaumburg J, Diekmann O, Hagendorff P, Bergmann S, Rohde M, et al. (2004) The cell wall subproteome of Listeria monocytogenes. Proteomics 4: 2991-3006.

44. Trost M, Wehmhöner D, Kärst U, Dieterich G, Wehland J, et al. (2005) Comparative proteome analysis of secretory proteins from pathogenic and nonpathogenic Listeria species. Proteomics 5: 1544-1557.

45. Watt SA, Wilke A, Patschkowski T, Niehaus K (2005) Comprehensive analysis of the extracellular proteins from Xanthomonas campestris pv. campestris B100. Proteomics 5: 153-167.

**Table 1. Secretion systems in *X. nematophila* and *X. bovienii***

|  | ***X. nematophila*** | ***X. bovienii*** | ***P. luminescens*** | ***P. asymbiotica*** |
| --- | --- | --- | --- | --- |
| Type I | 2 of 3 components*^e^* | 1 of 3 components*^b^* | Yes | 1 of 3 components*^b^* |
| Type II | No | No | No | No |
| Type III | No | No | Yes | Yes |
| Type IV | No | No | No | No |
| Type Va | No | No | No | No |
| Type Vb | Yes | Yes | Yes | Yes |
| Type Vc | No | No | No | No |
| Type VI | Yes*^c^* | Yes*^c,d^* | Yes*^c^* | Yes*^c^* |
| Sec-SRP | Yes | Yes | Yes | Yes |
| TAT | 4 of 5 components*^e^* | 4 of 5 components*^e^* | 4 of 5 components*^e^* | 4 of 5 components*^e^* |

*^a^* missing the ABC transporter HlyB

*^b^* only has the OMP TolC

*^c^* missing regulatory proteins

*^d^* missing VgrG (manual searches and Table 2 reveal that all four species have multiple VgrG-like proteins)

*^e^* missing TatE

**Table 2.** Distribution of Class A Type VI Secretion System Genes in *Xenorhabdus* and *Photorhabdus*

| **Locus Tag ^b^** | **Predicted protein product** | ***X. nematophila*^b^** | ***X. bovienii*** | ***P. asymbiotica*** | ***P. luminescens*** |
| --- | --- | --- | --- | --- | --- |
| XNC1_2515 | Vgr family protein | 4 | 9 | 17 | 14 |
| XNC1_2516 | Pentapeptide repeat protein | 2 | 2 | 3 | 0 |
| XNC1_2517 | Pentapeptide repeat protein | 2 | 2 | 3 | 0 |
| XNC1_2518 | conserved hypothetical protein | 1 | 1 | 1 | 0 |
| XNC1_2519 | conserved hypothetical protein | 1 | 1 | 1 | 0 |
| XNC1_2520 | putative lipoprotein of SST VI cluster | 1 | 1 | 0 | 0 |
| XNC1_2521 | probable component of SST VI cluster | 2 | 2 | 5 | 4 |
| XNC1_2222 | Conserved hypothetical protein with OmpA/MotB domains | 2 | 2 | 5 | 4 |
| XNC1_2523 | IcmF-like domain protein of SST VI cluster | 2 | 2 | 5 | 7 |
| XNC1_2524 | probable component of SST VI cluster | 1 | 1 | 1 | 0 |
| XNC1_2525 | probable component of SST VI cluster | 2 | 2 | 5 | 3 |
| XNC1_2526 | probable component of SST VI cluster | 3 | 2 | 5 | 4 |
| XNC1_2527 | putative Type VI secretion system effector (Hcp1 family) | 1 | 1 | 1 | 0 |
| XNC1_2528 | probable component of SST VI cluster | 1 | 1 | 1 | 0 |
| XNC1_2529 | probable component of SST VI cluster | 1 | 1 | 1 | 0 |
| XNC1_2530 | probable component of SST VI cluster) | 4 | 6 | 7 | 5 |
| XNC1_2531 | conserved hypothetical protein (probable component of SST VI cluster | 2 | 2 | 4 | 2 |
| XNC1_2532 | putative ClpA/B-type chaperone (Putative ATPase with chaperone activity) | 3 | 4 | 6 | 5 |

^a^ Locus tag IDs are according to the *X. nematophila* genome annotation

^b^ Number of hits retrieved when *X. nematophila* ORF used in BlastP analysis of whole genome with cutoff score of 1e-5.
