# Supplementary material for: The Entomopathogenic Bacterial Endosymbionts Xenorhabdus and Photorhabdus: Convergent Lifestyles from Divergent Genomes
Source: PLoS One. 2011 Nov 18;6(11):e27909. doi: 10.1371/journal.pone.0027909 (PMC3220699; doi:10.1371/journal.pone.0027909)
Supplement: Text S4 — Small RNAs. (DOC) [file pone.0027909.s012.doc]

# Text S4: Small RNAs

Brian C. Tjaden^1^ and Heidi Goodrich-Blair^2^

^1^Department of Computer Science, Wellesley College, Wellesley, Massachusetts, United States of America

^2^Department of Bacteriology, University of Wisconsin-Madison, Madison, Wisconsin, United States of America

E-mail: hgblair@bact.wisc.edu

Small RNAs have recently been recognized as important regulatory factors controlling gene expression in bacteria. Many are part of regulatory hierarchies that control adaptations to environmental changes [1], and it is likely that they will be increasingly found to control symbiotic gene expression in response to the host environment. In *E. coli*, small RNAs regulate translation of the sigma transcription factor RpoS (reviewed in [2]). This sigma factor is essential for *X. nematophila* colonization of its nematode host [3,4]. sRNAs can modulate RNA polymerase, protein degradation, and translation regulation [5].

Prediction algorithms were used to examine the small RNA coding capacity of *Xenorhabdus* spp. Using conserved RNA structure and primary sequence similarity search parameters, of the 59 *E. coli* small RNAs reported in the Rfam database [6], 23 were found either in *X. nematophila* or *X. bovienii* (Table 1, below). These 23 include those involved in gene expression machinery (e.g. 6S RNA, a regulator of transcription [7]; RNaseP necessary for tRNA processing; tmRNA, which releases stalled ribosomes; 4.5S RNA, a component of the secretion apparatus). Of those small RNAs known to regulate RpoS in *E. coli*, DsrA, RprA, and OxyS [8], only an RprA homolog was apparent in *Xenorhabdus* spp., with E-values 0.00046 and 0.0011 in *X. nematophila* and *X. bovienii* respectively and truncated lengths relative to *E. coli*. Therefore, regulatory control of RpoS expression in *Xenorhabdus* is distinct from that occurring in *E. coli*.

Small RNA genes were predicted throughout the genomes of *X. nematophila* and *X. bovienii* using a Markov model approach combining primary sequence data (e.g., dinucleotide frequency information, transcription termination signals) with conserved RNA structure information (e.g., compensatory basepair mutations, which conserve RNA secondary structure across related genomes) [9]. For the comparative genomics analysis, the following genomes were employed: *X. nematophila*, *X. bovienii*, *P. luminescens*, *Y. pestis*, *S. typhimurium*, *E. coli*, and *Klebsiella pneumoniae*. The computational approach predicted 93 and 105 small RNA genes in the intergenic regions of the *X. nematophila* (Table 2, below) and *X. bovienii* (Table 3, below) genomes respectively. Five of the 93 small RNA predictions in *X. nematophila* correspond to putative RNA genes in *E. coli* - RyeE, GcvB, RNaseP, Spot42, and RyhB. Three of the 105 small RNA predictions in *X. bovienii* correspond to putative RNA genes in *E. coli* - SraG, RprA, and RNaseP. Fewer computationally predicted small RNAs in *Xenorhabdus* spp. correspond to small RNA genes in *E. coli* than reported in Table 1 owing to stricter requirements for the computationally predicted small RNAs, namely a more stringent similarity requirement for a candidate RNA gene when compared to other genomes and the additional requirement of primary sequence evidence, such as a transcription termination signal. Nearly half of the small RNAs predicted in either *X. nematophila* or *X. bovienii* have apparent homologs in the other genome.

**References**

1. Repoila F, Majdalani N, Gottesman S (2003) Small non-coding RNAs, co-ordinators of adaptation processes in *Escherichia coli:* the RpoS paradigm. Mol Microbiol 48: 855-861.

2. Hengge-Aronis R (2002) Signal transduction and regulatory mechanisms involved in control of the sigma(S) (RpoS) subunit of RNA polymerase. Microbiol Mol Biol Rev 66: 373-395, table of contents.

3. Heungens K, Cowles CE, Goodrich-Blair H (2002) Identification of *Xenorhabdus nematophila* genes required for mutualistic colonization of *Steinernema carpocapsae* nematodes. Mol Microbiol 45: 1337-1353.

4. Vivas EI, Goodrich-Blair H (2001) *Xenorhabdus nematophilus* as a model for host-bacterium interactions: *rpoS* is necessary for mutualism with nematodes. J Bacteriol 183: 4687-4693.

5. Majdalani N, Vanderpool CK, Gottesman S (2005) Bacterial small RNA regulators. Crit Rev Biochem Mol Biol 40: 93-113.

6. Gardner PP, Daub J, Tate JG, Nawrocki EP, Kolbe DL, et al. (2009) Rfam: updates to the RNA families database. Nucleic Acids Res 37: D136-140.

7. Wassarman KM (2007) 6S RNA: a small RNA regulator of transcription. Curr Opin Microbiol 10: 164-168.

8. Gottesman S (2004) The small RNA regulators of *Escherichia coli:* roles and mechanisms*. Annu Rev Microbiol 58: 303-328.

9. Tjaden B (2008) TargetRNA: a tool for predicting targets of small RNA action in bacteria. Nucleic Acids Res 36: W109-113.

**Table 1.** *E. coli* small RNA predictions in *Xenorhabdus* spp.

| ***E. coli* RNA** | ***E. coli* sRNA length** | ***X. nem***  **E value** | ***X. nem* start** | ***X. nem* stop** | ***X. nem* length** | **Strand** | **GC%** | ***X. bov***  **E value** | ***X. bov* start** | ***X. bov* stop** | ***X. bov* length** | **Strand** | **GC%** |
| --- | --- | --- | --- | --- | --- | --- | --- | --- | --- | --- | --- | --- | --- |
| SRP_bact | 100 | 4.60E-11 | 835312 | 835411 | 100 | - | 0.55 | 4.40E-11 | 1585628 | 1585727 | 100 | - | 0.55 |
| RybB | 79 | 1.90E-06 | 1411566 | 1411643 | 78 | + | 0.435897 | 6.90E-06 | 918221 | 918295 | 75 | - | 0.413333 |
| SraB | 169 | 0.38 | 2714051 | 2714131 | 81 | - | 0.308642 | 0.023 | 2812642 | 2812725 | 84 | - | 0.321429 |
| RprA | 108 | 0.00046 | 1784712 | 1784789 | 78 | - | 0.358974 | 0.0011 | 2484600 | 2484663 | 64 | + | 0.390625 |
| SraC_RyeA | 145 | 2.80E-06 | 1827391 | 1827532 | 142 | + | 0.401408 | NA | NA | NA | NA | NA | NA |
| RyeB | 100 | 0.00016 | 1827395 | 1827476 | 82 | + | 0.439024 | NA | NA | NA | NA | NA | NA |
| CyaR_RyeE | 86 | 0.02 | 2650874 | 2650960 | 87 | + | 0.448276 | 0.031 | 2719512 | 2719596 | 85 | + | 0.411765 |
| IS128 | 209 | 0.036 | 356985 | 357152 | 168 | - | 0.196429 | 0.16 | 132643 | 132767 | 125 | + | 0.2 |
| GlmZ_SraJ | 185 | 6.70E-08 | 3211484 | 3211605 | 122 | - | 0.491803 | 0.00036 | 3003278 | 3003374 | 97 | + | 0.453608 |
| GlmY_tke1 | 148 | 8.30E-08 | 3211484 | 3211598 | 115 | - | 0.504348 | 0.00021 | 3003278 | 3003375 | 98 | + | 0.44898 |
| tmRNA | 362 | 8.90E-70 | 3175270 | 3175632 | 363 | - | 0.53719 | 3.10E-72 | 3103104 | 3103466 | 363 | + | 0.534435 |
| GcvB | 206 | 1.50E-19 | 3891299 | 3891497 | 199 | + | 0.41206 | 2.40E-18 | 495592 | 495790 | 199 | + | 0.386935 |
| 6S | 184 | 2.80E-20 | 983488 | 983671 | 184 | - | 0.48913 | 6.40E-22 | 3346709 | 3346892 | 184 | + | 0.516304 |
| QUAD | 151 | 0.15 | 743102 | 743202 | 101 | + | 0.425743 | NA | NA | NA | NA | NA | NA |
| RNaseP | 377 | 1.00E-37 | 4012491 | 4012703 | 213 | + | 0.586854 | 1.40E-37 | 3737153 | 3737368 | 216 | - | 0.583333 |
| SraG | 172 | 0.016 | 439865 | 439961 | 97 | - | 0.484536 | 3.40E-08 | 379366 | 379486 | 121 | - | 0.438017 |
| SraH | 108 | 0.00096 | 4019891 | 4019953 | 63 | + | 0.492063 | 0.00054 | 3730855 | 3730900 | 46 | - | 0.608696 |
| RyhB | 65 | 0.0002 | 4393187 | 4393257 | 71 | + | 0.352113 | 8.60E-07 | 2451030 | 2451096 | 67 | + | 0.432836 |
| istR | 130 | 0.98 | 4237257 | 4237327 | 71 | - | 0.521127 | 0.36 | 236246 | 236368 | 123 | - | 0.536585 |
| GlmZ_SraJ | 207 | 0.003 | 334566 | 334659 | 94 | + | 0.404255 | 1.10E-11 | 4000191 | 4000399 | 209 | - | 0.406699 |
| GlmY_tke1 | 149 | 0.42 | 334566 | 334600 | 35 | + | 0.514286 | 1.90E-09 | 4000250 | 4000397 | 148 | - | 0.439189 |
| Spot_42 | 119 | 1.10E-15 | 4163052 | 4163170 | 119 | + | 0.420168 | 3.80E-16 | 3941706 | 3941821 | 116 | - | 0.413793 |
| P26 | 62 | NA | NA | NA | NA | NA | NA | 0.0073 | 3911154 | 3911210 | 57 | - | 0.45614 |

**Table S2.** *X. nematophila* small RNA predictions

| **RNA** | **RNA start** | **RNA end** | **Strand** | ***E. coli* Gene** | **GC%** | **Left Gene Name** | **Left Gene Product** | **Right Gene Name** | **Right Gene Product** | ***X. bovienii* homolog** |
| --- | --- | --- | --- | --- | --- | --- | --- | --- | --- | --- |
| XnsRNA1 | 28035 | 28142 | + | - | 35.2 | glmU | bifunctional N-acetyl glucosamine-1-phosphate uridyltransferase/glucosamine-1-phosphate acetyl transferase | atpC | membrane-bound ATP synthase, F1 sector subunit epsilon | XbsRNA2 |
| XnsRNA2 | 55538 | 55697 | - | - | 47.5 | XNC1_0051 | inner membrane protein YjcH | acs | acetyl-CoA synthetase | - |
| XnsRNA3 | 103206 | 103422 | - | - | 40.1 | XNC1_0107 | hypothetical protein | cpxP | putative negative regulator | XbsRNA101 |
| XnsRNA4 | 160284 | 160425 | + | - | 38.7 | XNC1_0172 | WalR protein | rpmG | 50S ribosomal subunit protein L33 | XbsRNA103 |
| XnsRNA5 | 277448 | 277511 | - | - | 35.9 | rpmJ | 50S ribosomal subunit protein X | rpsM | 30S ribosomal subunit protein S13 | XbsRNA98 |
| XnsRNA6 | 280480 | 280657 | - | - | 34.8 | rplQ | 50S ribosomal subunit protein L17 | zntR | Zn(II)-responsive transcriptional regulator | XbsRNA97 |
| XnsRNA7 | 389707 | 389767 | + | - | 34.4 | hfq | host factor I for bacteriophage Q beta replication | hflX | putative GTPase | XbsRNA10 |
| XnsRNA8 | 393729 | 393790 | + | - | 38.7 | hflC | hypothetical protein | purA | adenylosuccinate synthetase | XbsRNA11 |
| XnsRNA9 | 433014 | 433087 | - | - | 39.2 | XNC1_0502 | hypothetical protein | XNC1_0503 | hypothetical protein | XbsRNA13 |
| XnsRNA10 | 570006 | 570152 | + | - | 36.1 | XNC1_0656 | small ubiquitous RNA-binding protein required for normal growth, cytoplasmic | zntA | Pb/Cd/Zn/Hg transporting ATPase (P-type ATPase family) | XbsRNA16 |
| XnsRNA11 | 600531 | 600589 | - | - | 44.1 | arcA | response regulator in two-component regulatory system with ArcB (or CpxA) | thrA | bifunctional aspartokinase I/homoserine dehydrogenase I , threonine sensitive | - |
| XnsRNA12 | 748161 | 748235 | - | - | 37.3 | XNC1_0877 | hypothetical protein | eno | enolase | - |
| XnsRNA13 | 847916 | 848025 | + | - | 41.8 | ppiD | peptidyl-prolyl cis/trans isomerase | hupB | DNA-binding protein HU-beta, NS1 | XbsRNA50 |
| XnsRNA14 | 854140 | 854400 | + | - | 38.3 | clpP | proteolytic subunit of clpA-clpP ATP-dependent serine protease | tig | peptidyl-prolyl cis/trans isomerase | XbsRNA53 |
| XnsRNA15 | 856003 | 856082 | - | - | 31.2 | tig | peptidyl-prolyl cis/trans isomerase | bolA | morphogenic pathway transcriptional activator | XbsRNA54 |
| XnsRNA16 | 858861 | 858930 | + | - | 35.7 | ampG | muropeptide transport protein | XNC1_0989 | hypothetical protein | - |
| XnsRNA17 | 915721 | 916165 | + | - | 36.2 | leuA | 2-isopropylmalate synthase | XNC1_1042 | hypothetical protein | - |
| XnsRNA18 | 922407 | 922471 | + | - | 36.9 | ilvH | acetolactate synthase III, valine-sensitive small subunit | fruR | carbon and energy metabolism transcriptional regulator | XbsRNA89 |
| XnsRNA19 | 961112 | 961273 | + | - | 45.7 | aceF | pyruvate dehydrogenase, dihydrolipoyltransacetylase subunit | lpd | dihydrolipoamide dehydrogenase , component of the 2-oxoglutarate dehydrogenase and the pyruvate dehydrogenase complexes | XbsRNA88 |
| XnsRNA20 | 1013925 | 1014175 | + | - | 43.0 | recJ | ssDNA exonuclease, 5'-> 3'-specific, Mg-dependent | prfB | peptide chain release factor RF-2 | - |
| XnsRNA21 | 1047711 | 1047792 | + | - | 35.4 | fabG | 3-oxoacyl-[acyl-carrier-protein] reductase | XNC1_1174 | hypothetical protein | XbsRNA41 |
| XnsRNA22 | 1126888 | 1126940 | + | - | 37.7 | recA | DNA strand exchange and recombination protein | alaS | alanyl-tRNA synthetase | - |
| XnsRNA23 | 1129821 | 1129877 | + | - | 43.9 | alaS | alanyl-tRNA synthetase | csrA | carbon storage regulator | XbsRNA83 |
| XnsRNA24 | 1133127 | 1133283 | - | - | 37.6 | gshA | gamma-glutamate-cysteine ligase | luxS | quorum-sensing protein, produces autoinducer-acyl-homoserine lactone-signaling molecules | XbsRNA82 |
| XnsRNA25 | 1137694 | 1137814 | + | - | 39.7 | ffh | 4.5S-RNP protein, GTP-binding export factor, part of signal recognition particle with 4.5 RNA | rpsP | 30S ribosomal subunit protein S16 | XbsRNA81 |
| XnsRNA26 | 1150654 | 1150777 | + | - | 29.8 | clpB | ATP-dependent protease | XNC1_1284 | hypothetical protein | XbsRNA79 |
| XnsRNA27 | 1161558 | 1161857 | - | - | 47.7 | XNC1_1295 | hypothetical protein | yfiF | putative tRNA /rRNA methyltransferase | - |
| XnsRNA28 | 1182330 | 1182496 | - | - | 35.3 | emrA | multidrug resistance secretion protein | mprA | multidrug resistance pump transcriptional repressor | - |
| XnsRNA29 | 1220047 | 1220211 | + | - | 30.3 | nagE | PTS family N-acetylglucosamine-specific transporter subunit IICBA | glnS | glutamine tRNA synthetase | - |
| XnsRNA30 | 1286985 | 1287315 | - | - | 34.1 | sdhB | succinate dehydrogenase, Fe-S protein | sucA | 2-oxoglutarate decarboxylase, component of the 2-oxoglutarate dehydrogenase complex, thiamin-binding | XbsRNA34 |
| XnsRNA31 | 1404786 | 1405260 | - | - | 32.2 | hisG | ATP phosphoribosyltransferase | yeeZ | hypothetical protein | XbsRNA30 |
| XnsRNA32 | 1419622 | 1419878 | + | - | 35.0 | cspD | DNA replication inhibitor | clpS | modulator of ClpA substrate specificity | - |
| XnsRNA33 | 1439046 | 1439109 | + | - | 37.5 | pflA | pyruvate formate lyase activating enzyme 1 | pflB | pyruvate formate lyase I | - |
| XnsRNA34 | 1454844 | 1455021 | - | - | 37.6 | ihfB | integration host factor (IHF) subunit beta | ycaI | putative recombination protein with metallo-hydrolase domain | - |
| XnsRNA35 | 1475099 | 1475340 | + | - | 36.8 | aspC | aspartate aminotransferase, PLP-dependent | opnP | outer membrane protein, OpnP | - |
| XnsRNA36 | 1476700 | 1476758 | - | - | 35.6 | opnP | outer membrane protein, OpnP | asnS | asparagine tRNA synthetase | XbsRNA25 |
| XnsRNA37 | 1517878 | 1517994 | + | - | 37.6 | motA | proton conductor component of motor, torque generator | flhC | flagellar class II biosynthesis transcriptional regulator | - |
| XnsRNA38 | 1747246 | 1747367 | - | - | 32.8 | XNC1_1819 | hypothetical protein | XNC1_1820 | Chelated iron transport system membrane protein yfeD | - |
| XnsRNA39 | 1852085 | 1852266 | - | - | 39.6 | XNC1_1952 | hypothetical protein | XNC1_1953 | hypothetical protein | XbsRNA73 |
| XnsRNA40 | 2018049 | 2018253 | + | - | 26.8 | XNC1_2106 | hypothetical protein | prs | phosphoribosylpyrophosphate synthetase | - |
| XnsRNA41 | 2184091 | 2184227 | + | - | 31.4 | fnr | Fumarate and nitrate reduction regulatory protein | XNC1_2243 | putative Tyrosine decarboxylase | - |
| XnsRNA42 | 2204802 | 2205089 | + | - | 33.0 | mmsA | methylmalonate-semialdehyde dehydrogenase, oxidoreductase protein | XNC1_2265 | hypothetical protein | - |
| XnsRNA43 | 2320296 | 2320771 | + | - | 34.0 | XNC1_2389 | hypothetical protein | XNC1_2390 | hypothetical protein | XbsRNA71 |
| XnsRNA44 | 2332715 | 2332765 | + | - | 35.3 | rnb | RNase II, mRNA degradation | XNC1_2405 | hypothetical protein | - |
| XnsRNA45 | 2438546 | 2438774 | - | - | 27.1 | hns | transcriptional regulator | XNC1_2486 | putative epimerase | XbsRNA63 |
| XnsRNA46 | 2514965 | 2515094 | - | - | 43.8 | XNC1_2554 | hypothetical protein | XNC1_2557 | putative transposase | XbsRNA96 |
| XnsRNA47 | 2563005 | 2563117 | - | - | 32.7 | XNC1_2586 | putative phage-like protein | intB | KpLE2 phage-like element; P4-like integrase | - |
| XnsRNA48 | 2564483 | 2564556 | - | - | 29.7 | intB | KpLE2 phage-like element; P4-like integrase | pgsA | phosphatidylglycerophosphate synthetase | XbsRNA70 |
| XnsRNA49 | 2580446 | 2580685 | + | - | 36.2 | XNC1_2613 | putative ATPase | ydaN | putative membrane associated protein | - |
| XnsRNA50 | 2589431 | 2589692 | - | - | 37.4 | pspF | phage shock protein transcriptional activator | sapA | peptide ABC transporter periplasmic-binding protein | - |
| XnsRNA51 | 2602741 | 2602801 | - | - | 26.2 | CjrC | putative outer membrane siderophore receptor CjrC | XNC1_2643 | protease II (fragment) | - |
| XnsRNA52 | 2650796 | 2650962 | + | RyeE | 35.3 | yegQ | putative protease | fbaB | fructose-bisphosphate aldolase | - |
| XnsRNA53 | 2653459 | 2653579 | - | - | 25.6 | thiD | bifunctional hydroxy-methylpyrimidine kinase (HMP kinase)/ hydroxy-phosphomethylpyrimidine kinase (HMP-P kinase) | cspC | cold shock protein, transcription antiterminator | - |
| XnsRNA54 | 2719389 | 2719562 | + | - | 29.3 | rne | RNase E: endoribonuclease for rRNA processing and mRNA degradation | XNC1_2744 | hypothetical protein | XbsRNA74 |
| XnsRNA55 | 2731979 | 2732330 | - | - | 28.1 | XNC1_2757 | hypothetical protein | cirA | outer membrane pore protein, receptor for colicin I, requires TonB | XbsRNA75 |
| XnsRNA56 | 2734299 | 2734393 | + | - | 30.5 | cirA | outer membrane pore protein, receptor for colicin I, requires TonB | XNC1_2759 | putative ferric enterobactin ABC transporter ATP-binding protein | - |
| XnsRNA57 | 2802933 | 2803182 | + | - | 40.8 | pta | phosphotransacetylase | ackA | acetate kinase A | - |
| XnsRNA58 | 2865408 | 2865655 | + | - | 35.9 | rcsC | hybrid sensory histidine kinase | gyrA | DNA gyrase subunit A, type II topoisomerase | XbsRNA23 |
| XnsRNA59 | 3023557 | 3023719 | + | - | 42.9 | XNC1_3048 | bifunctional prepilin peptidase leader peptidase/ methyl transferase (fragment) | XNC1_3049 | hypothetical protein | XbsRNA85 |
| XnsRNA60 | 3080045 | 3080173 | - | - | 34.1 | mltC | lytic murein transglycosylase C, membrane-bound | XNC1_3129 | putative phage integrase | - |
| XnsRNA61 | 3080250 | 3080420 | - | - | 31.0 | mltC | lytic murein transglycosylase C, membrane-bound | XNC1_3129 | putative phage integrase | - |
| XnsRNA62 | 3094034 | 3094124 | - | - | 44.0 | yejK | nucleotide associated protein, present in spermidine nucleoids | rplY | 50S ribosomal subunit protein L25 | XbsRNA76 |
| XnsRNA63 | 3103473 | 3103553 | - | - | 46.9 | yfcG | putative glutathione S-transferase | yfcH | putative cell division inhibitor | - |
| XnsRNA64 | 3128052 | 3128129 | + | - | 28.2 | aroC | chorismate synthase | prmB | N5-glutamine methyltransferase | - |
| XnsRNA65 | 3150547 | 3150640 | + | - | 36.2 | gltX | glutamate tRNA synthetase, catalytic subunit | XNC1_3206 | hypothetical protein | - |
| XnsRNA66 | 3157897 | 3158205 | - | - | 31.7 | cysK | subunit of cysteine synthase A and O-acetylserine sulfhydrolase A, PLP-dependent enzyme | XNC1_3212 | hypothetical protein | XbsRNA77 |
| XnsRNA67 | 3229584 | 3229738 | - | - | 33.5 | iscX | putative Fe-S clusters assembly protein | pepB | aminopeptidase B | - |
| XnsRNA68 | 3240606 | 3240679 | + | - | 45.9 | ndk | nucleoside diphosphate kinase | XNC1_3312 | hypothetical protein | - |
| XnsRNA69 | 3268132 | 3268262 | - | - | 37.4 | int | integrase | alpA | prophage CP4-57 regulatory protein | - |
| XnsRNA70 | 3268738 | 3268808 | - | - | 50.7 | alpA | prophage CP4-57 regulatory protein | Alpha | putative P4-specific DNA primase | XbsRNA39 |
| XnsRNA71 | 3329120 | 3329276 | + | - | 47.8 | XNC1_3424 | putative sugar fermentation stimulation protein | XNC1_3425 | putative terminase large subunit | XbsRNA95 |
| XnsRNA72 | 3353381 | 3353520 | + | - | 39.3 | insB | insertion element IS1 | XNC1_3458 | hypothetical protein | - |
| XnsRNA73 | 3390562 | 3390640 | + | - | 49.4 | XNC1_3502 | hypothetical protein | XNC1_3505 | hypothetical protein | XbsRNA47 |
| XnsRNA74 | 3426726 | 3426816 | - | - | 50.5 | XNC1_3551 | hypothetical protein | XNC1_3552 | hypothetical protein | XbsRNA87 |
| XnsRNA75 | 3482964 | 3483134 | + | - | 30.4 | XNC1_3598 | putative phage integrase | yjdC | putative regulator with DNA-binding domain | - |
| XnsRNA76 | 3509969 | 3510063 | + | - | 56.8 | XNC1_3638 | transposase | XNC1_3639 | putative phage integrase | - |
| XnsRNA77 | 3511590 | 3511872 | - | - | 36.7 | XNC1_3639 | putative phage integrase | XNC1_3640 | putative Protein-tyrosine kinase | XbsRNA42 |
| XnsRNA78 | 3528783 | 3529213 | - | - | 38.7 | XNC1_3661 | hypothetical protein | XNC1_3662 | Sugar fermentation stimulation protein B | XbsRNA45 |
| XnsRNA79 | 3561517 | 3561640 | + | - | 44.4 | XNC1_3690 | putative invasin | XNC1_3691 | hypothetical protein | - |
| XnsRNA80 | 3660911 | 3660988 | - | - | 37.2 | XNC1_3802 | hypothetical protein | mrxA | major fimbrial subunit polypeptide, mrfA | - |
| XnsRNA81 | 3661541 | 3661900 | - | - | 29.4 | mrxA | major fimbrial subunit polypeptide, mrfA | deoD | purine-nucleoside phosphorylase | - |
| XnsRNA82 | 3876663 | 3877063 | + | - | 39.2 | XNC1_4016 | hypothetical protein | syd | SecY interacting protein Syd | - |
| XnsRNA83 | 3891176 | 3891496 | + | GcvB | 38.9 | gcvA | transcriptional regulator | csdA | cysteine sulfinate desulfinase | - |
| XnsRNA84 | 3950716 | 3950801 | + | - | 33.7 | tolC | outer membrane channel | XNC1_4078 | hypothetical protein | - |
| XnsRNA85 | 3954629 | 3954987 | - | - | 42.3 | ribB | 3,4 dihydroxy-2-butanone-4-phosphate synthase | XNC1_4083 | hypothetical protein | - |
| XnsRNA86 | 3967571 | 3967656 | + | - | 47.7 | rpsU | 30S ribosomal subunit protein S21 | dnaG | DNA primase | - |
| XnsRNA87 | 4012450 | 4012951 | + | RNaseP | 53.0 | XNC1_4155 | hypothetical protein | yraL | hypothetical protein | XbsRNA93 |
| XnsRNA88 | 4039113 | 4039175 | - | - | 46.0 | yrbA | putative BolA family transcriptional regulator | XNC1_4185 | hypothetical protein | XbsRNA92 |
| XnsRNA89 | 4162751 | 4163169 | + | Spot42 | 36.5 | polA | 3' exonuclease | yihA | putative GTPase | - |
| XnsRNA90 | 4207073 | 4207187 | - | - | 54.8 | rpoB | RNA polymerase subunit beta | rpoC | RNA polymerase subunit beta' | - |
| XnsRNA91 | 4278257 | 4278494 | + | - | 34.9 | XNC1_4468 | hypothetical protein | glpD | sn-glycerol-3-phosphate dehydrogenase FAD/NAD(P)-binding | XbsRNA5 |
| XnsRNA92 | 4304556 | 4304923 | - | - | 31.8 | avtA | valine-pyruvate aminotransferase | XNC1_4501 | hypothetical protein | - |
| XnsRNA93 | 4393189 | 4393341 | + | RyhB | 34.6 | XNC1_4597 | protein yhhW | XNC1_4598 | transposase | - |

**Table 3.** *X. bovienii* small RNA predictions

| **RNA** | **RNA start** | **RNA end** | **Strand** | ***E. coli* Gene** | **GC%** | **Left Gene Name** | **Left Gene Product** | **Right Gene Name** | **Right Gene Product** | ***X. nematophila* homolog** |
| --- | --- | --- | --- | --- | --- | --- | --- | --- | --- | --- |
| XbsRNA1 | 24603 | 24707 | - | - | 42.9 | glmS | L-glutamine:D-fructose-6-phosphate aminotransferase | glmU | bifunctional N-acetyl glucosamine-1-phosphate uridyltransferase/glucosamine-1-phosphate acetyl transferase | - |
| XbsRNA2 | 26088 | 26195 | + | - | 34.3 | glmU | bifunctional N-acetyl glucosamine-1-phosphate uridyltransferase/glucosamine-1-phosphate acetyl transferase | atpC | membrane-bound ATP synthase, F1 sector subunit epsilon | XnsRNA1 |
| XbsRNA3 | 51613 | 51935 | + | - | 49.5 | hsrA | putative MFS family transport protein | yjcG | putative SSS family transport protein | - |
| XbsRNA4 | 97781 | 98037 | - | - | 32.3 | uspA | universal stress protein A, possibly linked to resistance to DNA-damage and respiratory uncoupling | gdhA | glutamate dehydrogenase, NADP-specific | - |
| XbsRNA5 | 140649 | 140869 | + | - | 31.7 | XBJ1_0137 | putative Acetyltransferase, GNAT family | glpD | sn-glycerol-3-phosphate dehydrogenase FAD/NAD(P)-binding (aerobic) | XnsRNA91 |
| XbsRNA6 | 184775 | 184825 | + | - | 43.1 | phoU | transcriptional repressor for high-affinity phosphate uptake | XBJ1_0193 | putative amino acid-binding protein (ABC superfamily, peri_bind) | - |
| XbsRNA7 | 250961 | 251021 | + | - | 47.5 | dppA | dipeptide ABC transporter periplasmic binding protein | dppB | dipeptide ABC transporter membrane protein | - |
| XbsRNA8 | 268412 | 268594 | + | - | 24.6 | XBJ1_0260 | hypothetical protein | XBJ1_0261 | hemolysin-coregulated protein Hcp | - |
| XbsRNA9 | 269814 | 270060 | + | - | 32.0 | XBJ1_0262 | hypothetical protein | XBJ1_0263 | hypothetical protein | - |
| XbsRNA10 | 331508 | 331568 | + | - | 37.7 | hfq | host factor I for bacteriophage Q beta replication, plays a role in degradation of RNA transcripts | hflX | putative GTPase subunit of protease with nucleoside triP hydrolase domain, together with HflC-HflK involved in stability of phage lambda cII repressor | XnsRNA7 |
| XbsRNA11 | 335498 | 335559 | + | - | 46.8 | hflC | FtsH phage lambda cII repressor protease | purA | adenylosuccinate synthetase | XnsRNA8 |
| XbsRNA12 | 362511 | 362561 | + | - | 49.0 | rpmA | 50S ribosomal subunit protein L27 | obgE | putative GTP-binding protein with nucleoside triP hydrolase domain | - |
| XbsRNA13 | 372446 | 372650 | + | - | 38.5 | secG | preprotein translocase membrane protein transport across inner membrane | XBJ1_0366 | YhbC-like protein (fragment) | XnsRNA9 |
| XbsRNA14 | 379369 | 379586 | - | SraG | 41.7 | rpsO | 30S ribosomal subunit protein S15 | pnp | polynucleotide phosphorylase, has polyadenylase activity | - |
| XbsRNA15 | 409860 | 410425 | - | - | 34.1 | pyrB | aspartate carbamoyltransferase, catalytic subunit | XBJ1_0401 | putative hydrolase | - |
| XbsRNA16 | 552269 | 552421 | + | - | 37.3 | sirA | small ubiquitous RNA-binding protein required for normal growth, cytoplasmic | zntA | Pb/Cd/Zn/Hg transporting ATPase (P-type ATPase family) | XnsRNA10 |
| XbsRNA17 | 633307 | 633459 | + | - | 41.8 | rcsF | regulator in colanic acid synthesis; overexpression confers mucoid phenotype, increases capsule synthesis | metQ | D-methionine ABC transporter periplasmic binding protein | - |
| XbsRNA18 | 645710 | 646032 | + | - | 49.8 | gmhB | D,D-heptose 1,7-bisphosphate phosphatase | XBJ1_0630 | hypothetical protein | - |
| XbsRNA19 | 652255 | 652313 | - | - | 57.6 | XBJ1_0632 | transposase | XBJ1_0633 | hypothetical protein | XnsRNA46 |
| XbsRNA20 | 672103 | 672160 | - | - | 56.9 | XBJ1_0663 | transposase | XBJ1_0664 | putative phage tail assembly protein | XnsRNA46 |
| XbsRNA21 | 697902 | 698057 | + | - | 41.0 | XBJ1_0704 | hypothetical protein | XBJ1_0705 | putative anti-repressor protein | - |
| XbsRNA22 | 707127 | 707375 | + | - | 33.3 | XBJ1_0717 | hypothetical protein | XBJ1_0718 | chitin-binding protein (fragment) | - |
| XbsRNA23 | 718654 | 718903 | - | - | 32.4 | gyrA | DNA gyrase subunit A, type II topoisomerase | rcsC | hybrid sensory histidine kinase in two-component regulatory system with RcsB and YojN, regulates capsule biosynthesis, cell division genes, OsmC expression | XnsRNA58 |
| XbsRNA24 | 741630 | 741695 | + | - | 51.5 | XBJ1_0750 | hypothetical protein | XBJ1_0751 | transposase | XnsRNA46 |
| XbsRNA25 | 809414 | 809472 | + | - | 30.5 | asnS | asparagine tRNA synthetase | ompN | outer membrane pore protein N, non-specific | XnsRNA36 |
| XbsRNA26 | 834479 | 834538 | - | - | 41.7 | rpsA | 30S ribosomal subunit protein S1 | cmk | cytidine monophosphate (CMP) kinase | - |
| XbsRNA27 | 835423 | 835521 | - | - | 38.4 | cmk | cytidine monophosphate (CMP) kinase | aroA | 3-enolpyruvylshikimate-5-phosphate synthetase | - |
| XbsRNA28 | 844984 | 845104 | + | - | 34.7 | pflB | pyruvate formate lyase I | pflA | pyruvate formate lyase activating enzyme 1 | XnsRNA33 |
| XbsRNA29 | 900574 | 900673 | - | - | 40.0 | XBJ1_0889 | putative cell division protein, required for chromosome partitioning (FstK) | lrp | transcriptional regulator of lrp regulon and for high-affinity branched-chain amino acid transport system (AsnC family) | - |
| XbsRNA30 | 924558 | 924767 | + | - | 36.2 | XBJ1_0915 | hypothetical protein | hisG | ATP phosphoribosyltransferase | XnsRNA31 |
| XbsRNA31 | 948195 | 948292 | - | - | 34.7 | metG | methionine tRNA synthetase | yohJ | putative transmembrane protein | - |
| XbsRNA32 | 950582 | 950768 | + | - | 27.3 | cdd | cytidine/deoxycytidine deaminase | sfcA | NAD-linked malate dehydrogenase | - |
| XbsRNA33 | 1015738 | 1016355 | - | - | 43.7 | XBJ1_1020 | hypothetical protein | XBJ1_1021 | hypothetical protein | - |
| XbsRNA34 | 1051641 | 1051920 | - | - | 35.4 | sucA | 2-oxoglutarate decarboxylase | sdhB | succinate dehydrogenase, Fe-S protein | XnsRNA30 |
| XbsRNA35 | 1132453 | 1132624 | + | - | 27.9 | XBJ1_1109 | chitobiase | glnS | glutamine tRNA synthetase | - |
| XbsRNA36 | 1134418 | 1134504 | - | - | 37.9 | glnS | glutamine tRNA synthetase | XBJ1_1111 | aminotransferase | - |
| XbsRNA37 | 1145581 | 1145670 | - | - | 36.7 | XBJ1_1123 | hypothetical protein | TetR | transcriptional regulator | - |
| XbsRNA38 | 1272049 | 1272108 | + | - | 48.3 | XBJ1_1289 | hypothetical protein | XBJ1_1290 | hypothetical protein | - |
| XbsRNA39 | 1299006 | 1299078 | - | - | 47.9 | alpA | CP4-57 prophage; transcriptional acitvator of a P4-like cryptic prophage | XBJ1_1318 | putative prophage primase | XnsRNA70 |
| XbsRNA40 | 1352190 | 1352309 | - | - | 36.7 | XBJ1_1385 | baseplate assembly protein GpV | XBJ1_1386 | putative endopeptidase | - |
| XbsRNA41 | 1363903 | 1363984 | - | - | 36.6 | XBJ1_1402 | hypothetical protein | XBJ1_1403 | 3-ketoacyl-CoA reductase | XnsRNA21 |
| XbsRNA42 | 1429109 | 1429267 | + | - | 34.6 | yjdC | putative regulatory protein | XBJ1_1459 | putative phage integrase | XnsRNA77 |
| XbsRNA43 | 1434500 | 1434589 | - | - | 55.6 | XBJ1_1465 | hypothetical protein | XBJ1_1466 | hypothetical protein | - |
| XbsRNA44 | 1435511 | 1435882 | - | - | 48.1 | XBJ1_1467 | putative Antirestriction plasmid protein | XBJ1_1469 | reverse transcriptase-like protein (fragment) | - |
| XbsRNA45 | 1499231 | 1499438 | + | - | 51.9 | sfsB | transcriptional activator of maltose metabolism | XBJ1_1541 | hypothetical protein | XnsRNA78 |
| XbsRNA46 | 1505884 | 1506338 | - | - | 44.2 | XBJ1_1549 | hypothetical protein | XBJ1_1550 | hypothetical protein | - |
| XbsRNA47 | 1518670 | 1518783 | - | - | 42.1 | XBJ1_1563 | hypothetical protein | XBJ1_1564 | hypothetical protein | XnsRNA73 |
| XbsRNA48 | 1567986 | 1568054 | - | - | 39.1 | hemH | ferrochelatase | adk | adenylate kinase | - |
| XbsRNA49 | 1595372 | 1595518 | + | - | 39.5 | XBJ1_1632 | putative DNA uptake protein and/or related DNA-binding protein | ppiD | peptidyl-prolyl cis-trans isomerase, for periplasmic folding of outer membrane proteins | - |
| XbsRNA50 | 1597392 | 1597572 | - | - | 40.3 | ppiD | peptidyl-prolyl cis-trans isomerase, for periplasmic folding of outer membrane proteins | hupB | DNA-binding protein HU-beta | XnsRNA13 |
| XbsRNA51 | 1598011 | 1598089 | - | - | 31.6 | hupB | DNA-binding protein HU-beta | lon | DNA-binding ATP-dependent protease La; heat shock K-protein | - |
| XbsRNA52 | 1600452 | 1600652 | + | - | 35.3 | lon | DNA-binding ATP-dependent protease La; heat shock K-protein | clpX | ATPase, chaperone subunit of serine protease | - |
| XbsRNA53 | 1602693 | 1602950 | - | - | 37.6 | clpP | clpA-clpP ATP-dependent serine protease proteolytic subunit | tig | peptidyl-prolyl cis/trans isomerase (trigger factor), molecular chaperone involved in cell division | XnsRNA14 |
| XbsRNA54 | 1604556 | 1604638 | - | - | 30.1 | XBJ1_1639 | hypothetical protein | bolA | transcriptional activator of morphogenic pathway (BolA family), important in general stress response | XnsRNA15 |
| XbsRNA55 | 1608397 | 1608819 | + | - | 33.3 | XBJ1_1645 | hypothetical protein | cyoA | cytochrome o ubiquinol oxidase subunit II | - |
| XbsRNA56 | 1704689 | 1704791 | + | - | 48.5 | dnaJ | heat shock protein (Hsp40), co-chaperone with DnaK | dnaK | chaperone Hsp70 in DNA biosynthesis/cell division | - |
| XbsRNA57 | 1825254 | 1825341 | + | - | 50.0 | XBJ1_1866 | hypothetical protein | XBJ1_1867 | transposase | - |
| XbsRNA58 | 1843603 | 1843816 | + | - | 28.0 | XBJ1_1898 | putative colanic acid capsular biosynthesis activation protein A | XBJ1_1899 | hypothetical protein | - |
| XbsRNA59 | 2026266 | 2026323 | + | - | 56.9 | XBJ1_2059 | putative phage tail assembly protein | XBJ1_2060 | transposase | XnsRNA46 |
| XbsRNA60 | 2212285 | 2212396 | + | - | 33.9 | XBJ1_2229 | putative peptidase protein YebA | lpxM | myristoyl transferase in lipid A biosynthesis, suppressor of htrB (lpxL) | - |
| XbsRNA61 | 2253466 | 2253860 | - | - | 38.2 | trpE | anthranilate synthase component I | XBJ1_2280 | protein trpH | - |
| XbsRNA62 | 2364456 | 2364700 | - | - | 29.8 | minE | cell division topological specificity factor, reverses MinC inhibition of FtsZ ring formation | XBJ1_2386 | transposase | - |
| XbsRNA63 | 2410279 | 2410503 | - | - | 29.3 | hns | transcriptional regulator, DNA-binding protein HLP-II (HU, BH2, HD, NS), increases DNA thermal stability | XBJ1_2433 | putative epimerase | XnsRNA45 |
| XbsRNA64 | 2413919 | 2414059 | - | - | 41.1 | galU | glucose-1-phosphate uridylyltransferase | rssB | response regulator involved in protein turnover, controls stability of RpoS | - |
| XbsRNA65 | 2484321 | 2484763 | + | RprA | 31.6 | ydiK | putative transport protein | pps | phosphoenolpyruvate synthase | - |
| XbsRNA66 | 2514496 | 2514712 | - | - | 40.1 | pheS | phenylalanine tRNA synthetase subunit alpha | rplT | 50S ribosomal subunit protein L20 | - |
| XbsRNA67 | 2522069 | 2522230 | + | - | 29.6 | htpX | heat shock protein, integral membrane protein | prc | carboxy-terminal protease for penicillin-binding protein 3 | - |
| XbsRNA68 | 2565990 | 2566080 | - | - | 44.0 | XBJ1_2589 | iron-regulated protein (cjrA) | maeB | putative bifunctional malic oxidoreductase /phosphotransacetylase | - |
| XbsRNA69 | 2619603 | 2619667 | + | - | 29.2 | XBJ1_2662 | putative major capsid protein (fragment) | XBJ1_2663 | hypothetical protein | - |
| XbsRNA70 | 2627917 | 2627990 | - | - | 28.4 | intB | prophage like-integrase | pgsA | phosphatidylglycerophosphate synthetase | XnsRNA48 |
| XbsRNA71 | 2757388 | 2757797 | + | - | 34.1 | XBJ1_2792 | hypothetical protein | XBJ1_2793 | hypothetical protein | XnsRNA43 |
| XbsRNA72 | 2758540 | 2758810 | - | - | 46.1 | XBJ1_2794 | hypothetical protein | XBJ1_2797 | hypothetical protein | - |
| XbsRNA73 | 2770285 | 2770444 | + | - | 33.1 | XBJ1_2812 | hypothetical protein | XBJ1_2813 | hypothetical protein | XnsRNA39 |
| XbsRNA74 | 2818005 | 2818066 | + | - | 32.3 | rne | RNase E | XBJ1_2868 | hypothetical protein | - |
| XbsRNA75 | 2824919 | 2825124 | + | - | 28.2 | XBJ1_2874 | hypothetical protein | cirA | outer membrane pore protein, receptor for colicin I, requires TonB | XnsRNA55 |
| XbsRNA76 | 2847567 | 2847656 | - | - | 42.2 | rplY | 50S ribosomal subunit protein L25 | yejK | nucleotide associated protein, present in spermidine nucleoids | XnsRNA62 |
| XbsRNA77 | 3070865 | 3070964 | - | - | 31.0 | cysK | subunit of cysteine synthase A and O-acetylserine sulfhydrolase A, PLP-dependent enzyme | XBJ1_3123 | hypothetical protein | XnsRNA66 |
| XbsRNA78 | 3104049 | 3104360 | - | - | 37.8 | XBJ1_3166 | hypothetical protein | XBJ1_3167 | putative PA-I galactophilic lectin (PA-IL) (Galactose-binding lectin) | - |
| XbsRNA79 | 3186012 | 3186135 | - | - | 32.3 | XBJ1_3253 | hypothetical protein | clpB | ATP-dependent protease, Hsp 100, part of multi-chaperone system with DnaK, DnaJ, and GrpE | XnsRNA26 |
| XbsRNA80 | 3192271 | 3192467 | - | - | 28.4 | yfiA | ribosome associated factor, stabilizes ribosomes against dissociation | pheA | bifunctional chorismate mutase P/prephenate dehydratase | - |
| XbsRNA81 | 3199110 | 3199227 | - | - | 36.4 | rpsP | 30S ribosomal subunit protein S16 | ffh | 4.5S-RNP protein, GTP-binding export factor, part of signal recognition particle with 4.5 RNA | XnsRNA25 |
| XbsRNA82 | 3206768 | 3206925 | + | - | 36.7 | luxS | quorum-sensing protein, produces autoinducer-acyl-homoserine lactone-signaling molecules | gshA | gamma-glutamate-cysteine ligase | XnsRNA24 |
| XbsRNA83 | 3209999 | 3210056 | - | - | 48.3 | csrA | carbon storage regulator, post-translational activator of flhDC expression, regulates biofilm formation, RNA-binding | alaS | alanyl-tRNA synthetase | XnsRNA23 |
| XbsRNA84 | 3248124 | 3248458 | - | - | 38.8 | XBJ1_3324 | Na(+)-translocating NADH-quinone reductase subunit A | XBJ1_3325 | hypothetical protein | - |
| XbsRNA85 | 3260542 | 3260699 | + | - | 44.3 | XBJ1_3336 | bifunctional prepilin peptidase: leader peptidase/methyl transferase (General Secretory Pathway) (fragment) | XBJ1_3337 | hypothetical protein | XnsRNA59 |
| XbsRNA86 | 3297722 | 3297930 | - | - | 49.8 | XBJ1_3382 | hypothetical protein | XBJ1_3383 | transcriptional activator of maltose metabolism | XnsRNA78 |
| XbsRNA87 | 3303772 | 3303863 | - | - | 54.3 | XBJ1_3389 | hypothetical protein | XBJ1_3390 | hypothetical protein | XnsRNA74 |
| XbsRNA88 | 3358669 | 3358846 | - | - | 41.6 | lpd | dihydrolipoamide dehydrogenase , component of the 2-oxoglutarate dehydrogenase and the pyruvate dehydrogenase complexes | aceF | pyruvate dehydrogenase, dihydrolipoyltransacetylase subunit | XnsRNA19 |
| XbsRNA89 | 3398040 | 3398103 | - | - | 35.9 | fruR | transcriptional regulator of the control of carbon and energy metabolism (GalR/LacI family) | ilvH | acetolactate synthase III, valine-sensitive, small subunit | XnsRNA18 |
| XbsRNA90 | 3473229 | 3473310 | - | - | 54.9 | XBJ1_3566 | transposase | efp | elongation factor P (EF-P) | XnsRNA46 |
| XbsRNA91 | 3475593 | 3475673 | - | - | 42.0 | yjeI | FAD/NAD(P)-binding domain-containing protein | groL | chaperone Hsp60 (GroEL), part of GroE chaperone system | - |
| XbsRNA92 | 3711925 | 3711978 | + | - | 42.6 | XBJ1_3834 | hypothetical protein | yrbA | putative transcriptional regulator | XnsRNA88 |
| XbsRNA93 | 3736938 | 3737409 | - | RNaseP | 54.2 | yraL | cobalt precorrin-4 methyltransferase domain-containing protein | XBJ1_3862 | hypothetical protein | XnsRNA87 |
| XbsRNA94 | 3790705 | 3790766 | - | - | 41.9 | XBJ1_3914 | putative capsid protein of prophage CP-933C (putative major head protein) (fragment) | XBJ1_3915 | hypothetical protein | - |
| XbsRNA95 | 3958692 | 3958836 | - | - | 39.3 | XBJ1_4114 | transcriptional activator of maltose metabolism | XBJ1_4115 | hypothetical protein | XnsRNA71 |
| XbsRNA96 | 3965857 | 3966101 | + | - | 43.7 | XBJ1_4125 | transposase | XBJ1_4126 | transposase | XnsRNA46 |
| XbsRNA97 | 4053428 | 4053580 | + | - | 32.0 | zntR | Zn(II)-responsive transcriptional regulator | rplQ | 50S ribosomal subunit protein L17 | XnsRNA6 |
| XbsRNA98 | 4056553 | 4056766 | - | - | 40.2 | rpsM | 30S ribosomal subunit protein S13 | secY | preprotein translocase membrane protein transport across inner membrane | XnsRNA5 |
| XbsRNA99 | 4067295 | 4067575 | + | - | 40.2 | rpsJ | 30S ribosomal subunit protein S10 | bfr | bacterioferritin, an iron storage homoprotein | - |
| XbsRNA100 | 4078552 | 4078874 | - | - | 49.5 | murB | UDP-N-acetylenolpyruvoylglucosamine reductase, FAD-binding | murI | glutamate racemase | - |
| XbsRNA101 | 4129667 | 4129761 | - | - | 43.2 | XBJ1_4309 | hypothetical protein | XBJ1_4310 | periplasmic protein | XnsRNA3 |
| XbsRNA102 | 4132480 | 4132828 | + | - | 31.8 | cpxA | sensory histidine kinase in two-component regulatory system with CpxR, regulates genes involved in folding/degrading periplasmic proteins, senses changes in the cell envelope | XBJ1_4313 | UDP-glucose 6-dehydrogenase | - |
| XbsRNA103 | 4176356 | 4176501 | + | - | 40.4 | XBJ1_4357 | WalR protein | rpmG | 50S ribosomal subunit protein L33 | XnsRNA4 |
| XbsRNA104 | 4206053 | 4206442 | - | - | 24.6 | XBJ1_4392 | putative Carboxylesterase | XBJ1_4393 | putative isochorismatase hydrolase | - |
| XbsRNA105 | 4211107 | 4211330 | + | - | 40.2 | XBJ1_4400 | hypothetical protein | XBJ1_4401 | replication initiation protein | - |
