# Supplementary material for: The Entomopathogenic Bacterial Endosymbionts Xenorhabdus and Photorhabdus: Convergent Lifestyles from Divergent Genomes
Source: PLoS One. 2011 Nov 18;6(11):e27909. doi: 10.1371/journal.pone.0027909 (PMC3220699; doi:10.1371/journal.pone.0027909)
Supplement: Text S5 — Toxins, Cytotoxins, and Invasins. (DOC) [file pone.0027909.s013.doc]

# NEEDS REFERENCES FORMATTED

# Text S5: Toxins, Cytotoxins, and Invasins

Alain Givaudan^1,2^, Jean-Claude Ogier^1,2^, Sophie Gaudriault^1,2^, Kelsea A. Jewell^3^, Garret Suen^3^, and Heidi Goodrich-Blair^3^

^1^Institut National de la Recherche Agronomique-Université de Montpellier II, Montpellier, France

^2^Université Montpellier, Montpellier, France

^3^Department of Bacteriology, University of Wisconsin-Madison, Madison, Wisconsin, United States of America

E-mail: hgblair@bact.wisc.edu

***Predicted* Xenorhabdus *insecticidal Tc toxins.*** The virulence activities of *Xenorhabdus* spp. are attributed to insecticidal toxins that allow them to suppress or evade immunity and kill the insect host. The toxin complex (Tc) family of large molecular weight protein toxins was initially discovered in *Photorhabdus* and *Xenorhabdus* spp., and was subsequently identified in other insect pathogens as well as in bacteria with no known association with insects . The Tc toxins comprise three components, A, B, and C. *X. nematophila* and *X. bovienii* encode seven and three intact A subunit genes; three and two intact B subunit genes; and three and two intact C subunit genes, respectively (Figure 1, Tables 1 and 2, below). Thus, *X. bovienii* appears to have the most limited Tc toxin repertoire among the sequenced strains of *Photorhabdus* and *Xenorhabdus* (Table 1, below). The A component of Tc complexes has toxin activity potentiated by the B and C components . Indeed, the oral toxicities encoded by the *X. nematophila* *xptA1* and *xptA2* A subunit genes have been previously described and reveal insect host specificity (the former is active against *Pierus brassicae* and *P. rapae* while the latter is active against *Heliothis virescens* ). These data suggest that the range of insects capable of being killed by *X. nematophila* may be broader than that of *X. bovienii*.

In fulfillment of an early prediction that the A and B subunits act as a delivery system to deliver the C subunit as the actual toxin , C subunits (TccC3 and TccC5 of *P. luminescens*) were shown recently to have ADP-ribosylating activity and effects on actin polymerization or Rho GTP-ase activity in host cells . Residues required for ADP-ribosylating activity are in the C-terminal region of TccC3 and TccC5 as part of an "RSE-motif" . To gain further insight into the relationship of the *Xenorhabdus* and *Photorhabdus* toxin subunits, we performed a phylogenetic analysis of the A subunit protein sequences. We found that these A subunits can be divided into three classes (I-III), with *X. bovienii* having a single representative from each class (Figure 1A and Table 2, below). An early analysis of C subunits We also performed an alignment of the C subunits and found that they consist of a highly conserved N-terminal region that spans the first ~750 amino acids followed by a highly variable region C-terminal region of ~300 amino acids. A phylogenetic analysis of these two regions reveals different evolutionary topologies. For the N-terminal region, our analyses show that with one exception, proteins from *Photorhabdus* clade together and away from a clade of *Xenorhabdus* proteins (Figure 1B, below). Furthermore, there is one *X. bovienii* N-terminal domain, XBJ1_3085 that forms its own distinct clade. In contrast to the N-terminal region, the 300 aa C-terminal domains of the C subunits are not conserved, and fall into 2 groups (I and II, Figure 1B, below). The larger (II) of these groups includes the *P. luminescens* ADP-ribosylating TccC3 and TccC5 homologs (plu0964 and plu0967) and only one *Xenorhabdus* homolog: XBJ1_3085. It will be of interest to determine if ADP-ribosylating activity is a defining characteristic of members of Group II, and if members of Group I encode a distinct activity.

A single Tc locus, containing two A subunits, one each of classes II and III, is conserved among *P. luminescens, P. asymbiotica, X. nematophila*, and *X. bovienii* (Figure 1C, below). This locus is distinct from other Tc coding regions in that the two Tc toxin-coding genes are flanked by genes predicted to encode chitinases. Chitinases can synergize insecticidal toxins , making this conserved genetic association particularly intriguing. Chitin is a major component of the insect peritrophic matrix separating the gut lumen from the hemocoel. Wilkinson *et al.* (2009) suggested that chitinase digestion of such chitinous tissues could facilitate Tc toxin access to epithelial target cells. Consistent with this idea, during natural *S. carpocapsae* infection, the *X. nematophila* bacterial symbiont can be released by defecation before the nematode vector has completely entered the hemocoel . Therefore, at least some *X. nematophila* cells may be released into the gut lumen, where degradation of the peritrichous membrane by a chitinase could contribute to disease progression. The recent finding that invertebrate hemocytes harbor chitin raises the further intriguing possibility that combined Tc toxin and chitinase activities may be more effective in killing and utilizing energy resources available in circulating immune cells. The conserved genomic linkage of A subunits from groups I and III may indicate synergistic function of these proteins.

In *Yersinia enterocolitica*, another insecticidal Tc toxin-encoding bacterium , Tc toxin is secreted through the Type 3 secretion system and regulated by a LysR-type transcriptional regulator . Since the *Xenorhabdus* spp. genomes lack genes encoding type III secretion components the Tc toxins must be secreted through a different system (See Text S3 and Discussion).

***Cytotoxins.*** *Xenorhabdus* genomes carry several CDSs that are similar to cytotoxins. However, genes and operons encoding putative toxins are often singular in *Xenorhabdus* genomes, while a striking feature of the genome of the closely related bacterium, *P. luminescens,* is the presence of repeated genes encoding cytolysin-related proteins . For instance, only one locus in each *Xenorhabdus* genome encodes the putative ABC transporter for RTX secretion, putative RTX toxin activating protein and a large protein homologous to the *Vibrio cholerae* RtxA toxin (XNC1_1381 and XBJ1_1089). In contrast, six full-length and four truncated genes homologous to RtxA toxins were identified in *Photorhabdus* TT01 genome. The *P. asymbiotica* genome encodes two full length and one truncated genes with similarity to *rtxA*. As another example, two contiguous CDSs, *xaxA* and *xaxB,* encode the active prototype of a new family of binary toxins, the XaxAB haemolysins, in *X. nematophila* . One couple of *xaxAB* homologs was also found in *X. bovienii* genome (XBJ1_1711 and XBJ1_1710, respectively). The XaxAB hemolytic locus is repeated twice in *P. luminescens* TT01 genome but only once in *P. asymbiotica*.

***Invasins.*** Analyses of *X. nematophila* and *X. bovienii* genomes revealed several similarities between these organisms in genes that encode putative invasins. Using the MaGe platform (http://www.genoscope.cns.fr/agc/microscope/xenorhabduscope), we found nine and four genes in *X. bovienii* and *X. nematophila* genomes, respectively, which encode putative invasins (Table 3). Each of these putative invasins has a “predicted invasin domain” (PRK10177) in their N-terminal region (For synthesis on invasins ). Among them, seven proteins share one or several “Ig-like domains” (BID_1 motif), and five proteins share a “salmonella repeat domain” (DUF823) in the C-terminal region. A phylogenetic tree based on the deduced amino acid sequences allows the segregation of these proteins into three distinct clusters (Figure 2): (i) uncharacterized invasins with representatives from both *Xenorhabdus* and *Photorhabdus* species (ii) *Xenorhabdus* (but not *Photorhabdus*) putative invasins characterized by a DUF domain (iii) *Xenorhabdus* (but not *Photorhabdus*) putative invasins characterized by at least five Ig-like domains and related to an *E. coli* Ig family protein. The members of this family are found in bacterial surface proteins such as intimins and invasins. The invasin proteins (Inv) of *Yersinia spp* are homologous to intimins (Int) and both proteins play a role in uptake into mammal cells. Because no study has been performed to give a function to ‘invasin-like’ annotated genes in *Xenorhabdus*, their occurrence in bacteria having an extracellular life cycle within insect bodies and nematodes remains intriguing.

A growing family of hemolysin/hemagglutinin-related proteins secreted by the two-partner secretion (TPS) pathway has been the focus of much interest in recent years, due to their role in virulence of Gram-negative pathogens. TpsA (the exoprotein) is translocated across the outer membrane by a dedicated β-barrel protein (TpsB) pore . A hallmark of TpsA proteins is their N-terminal TPS domain containing the presence of the NPNGI and NPNL conserved motifs. A survey of *Xenorhabdus* and *Photorhabdus* genomes revealed that the *X. nematophila* and *X. bovienii* genomes have only three and two couples of genes predicted to encode TpsAB proteins, respectively, whereas the *P. luminescens* and *P. asymbiotica* genomes each contain six couples of *tpsAB* genes (Table 4, below). TpsA proteins are often involved in virulence due to their roles in bacterial adherence, cytolysis, and iron uptake , but they are also involved in other processes like the contact inhibition of growth described in *E. coli* . A phylogenic tree based on TpsA sequences of *Xenorhabdus*, *Photorhabdus,* and functionally characterized TpsA from other bacteria allows the classification of TpsA exoproteins from *Xenorhabdus* into three groups (Figure 3, below). Cluster II comprises active haemolysins that have been described, such as XhlA from *X. nematophila (*XNC1_4556), PhlA from *P. luminescens (Plu0316)* , ShlA, the first haemolysin of the TPSS family to be characterized and haemagglutinins from *Bordetella*. As XBJ1_0258 is closely related to haemolysins, it is likely that this gene encode a functional TPS haemolysin in *X. bovienii*. No CDSs from *Xenorhabdus* are strongly related to the filamentous haemagglutinins of *Bordetella*. Cluster I contains CdiA, which is involved in the contact-dependent inhibition system of *E. coli.* No putative protein from *X. nematophila* belongs to cluster I. The presence of XBJ1_1979 in this group may suggest that the CdiAB system may occur in *X. bovienii*. However, *xbj1_1977* and *xbj1_1979* are probably gene fragments of a *tpsA* pseudogene disrupted by a transposase (*xbj1_1978*). Interestingly, cluster III contains TpsA proteins with unknown functions from *X. nematophila* (XNC1_3564 and XNC1_3688), *P. luminescens* and *P. asymbiotica*. As cluster III is only composed of TpsA proteins from bacterial symbionts of entomopathogenic nematodes, we can suggest that these proteins may be involved in specific interactions with the invertebrates.

**References**

1. Blackburn MB, Martin PA, Kuhar D, Farrar RR, Jr., Gundersen-Rindal DE (2011) The occurrence of *Photorhabdus*-Like toxin complexes in *Bacillus thuringiensis*. PLoS One 6: e18122.

2. ffrench-Constant R, Waterfield N (2006) An ABC guide to the bacterial toxin complexes. Adv Appl Microbiol 58: 169-183.

3. Waterfield N, Bowen DJ, Fetherston JD, Perry RD, ffrench-Constant RH (2001) The toxin complex genes of *Photorhabdus*: a growing gene family. Trends Microbiol 9: 185-191.

4. Waterfield N, Hares M, Yang G, Dowling A, ffrench-Constant R (2005) Potentiation and cellular phenotypes of the insecticidal toxin complexes of *Photorhabdus* bacteria. Cell Microbiol 7: 373-382.

5. Sergeant M, Jarrett P, Ousley M, Morgan JA (2003) Interactions of insecticidal toxin gene products from Xenorhabdus nematophilus PMFI296. Appl Environ Microbiol 69: 3344-3349.

6. Sheets JJ, Hey TD (2007) Isolation and characterization of two toxin complexes from *Xenorhabdus nematophilus*. Abstract, 40th Annual Meeting of the Society for Invertebrate Pathology.

7. Sheets JJ, Hey TD, Fencil KJ, Burton SL, Ni W, et al. (2011) Insecticidal toxin complex proteins from Xenorhabdus nematophilus: Structure and pore formation. J Biol Chem.

8. Morgan JA, Sergeant M, Ellis D, Ousley M, Jarrett P (2001) Sequence analysis of insecticidal genes from *Xenorhabdus* *nematophilus* PMFI296. Appl Environ Microbiol 67: 2062-2069.

9. Lang AE, Schmidt G, Schlosser A, Hey TD, Larrinua IM, et al. (2010) *Photorhabdus luminescens* toxins ADP-ribosylate actin and RhoA to force actin clustering. Science 327: 1139-1142.

10. Fieldhouse RJ, Merrill AR (2008) Needle in the haystack: structure-based toxin discovery. Trends Biochem Sci 33: 546-556.

11. Pardo-Lopez L, Munoz-Garay C, Porta H, Rodriguez-Almazan C, Soberon M, et al. (2009) Strategies to improve the insecticidal activity of Cry toxins from *Bacillus thuringiensis*. Peptides 30: 589-595.

12. Wilkinson P, Waterfield NR, Crossman L, Corton C, Sanchez-Contreras M, et al. (2009) Comparative genomics of the emerging human pathogen *Photorhabdus asymbiotica* with the insect pathogen *Photorhabdus luminescens*. BMC Genomics 10: 302.

13. Sicard M, Brugirard-Ricaud K, Pages S, Lanois A, Boemare NE, et al. (2004) Stages of infection during the tripartite interaction between *Xenorhabdus nematophila*, its nematode vector, and insect hosts. Appl Environ Microbiol 70: 6473-6480.

14. Heath-Heckman EAC, McFall-Ngai MJ (2011) The occurrence of chitin in the hemocytes of invertebrates. Zoology In press.

15. Pinheiro VB, Ellar DJ (2007) Expression and insecticidal activity of *Yersinia pseudotuberculosis* and *Photorhabdus luminescens* toxin complex proteins. Cell Microbiol 9: 2372-2380.

16. Gendlina I, Held KG, Bartra SS, Gallis BM, Doneanu CE, et al. (2007) Identification and type III-dependent secretion of the *Yersinia pestis* insecticidal-like proteins. Mol Microbiol 64: 1214-1227.

17. Duchaud E, Rusniok C, Frangeul L, Buchrieser C, Givaudan A, et al. (2003) The genome sequence of the entomopathogenic bacterium *Photorhabdus luminescens*. Nat Biotechnol 21: 1307-1313.

18. Vigneux F, Zumbihl R, Jubelin G, Ribeiro C, Poncet J, et al. (2007) The *xaxAB* genes encoding a new apoptotic toxin from the insect pathogen *Xenorhabdus nematophila* are present in plant and human pathogens. J Biol Chem 282: 9571-9580.

19. Niemann HH, Schubert WD, Heinz DW (2004) Adhesins and invasins of pathogenic bacteria: a structural view. Microbes Infect 6: 101-112.

20. Herbert EE, Goodrich-Blair H (2007) Friend and foe: the two faces of *Xenorhabdus nematophila*. Nat Rev Microbiol 5: 634-646.

21. Mazar J, Cotter PA (2007) New insight into the molecular mechanisms of two-partner secretion. Trends Microbiol 15: 508-515.

22. Hertle R (2005) The family of *Serratia* type pore forming toxins. Curr Protein Pept Sci 6: 313-325.

23. Molina MA, Ramos JL, Espinosa-Urgel M (2006) A two-partner secretion system is involved in seed and root colonization and iron uptake by *Pseudomonas putida* KT2440. Environ Microbiol 8: 639-647.

24. Aoki SK, Pamma R, Hernday AD, Bickham JE, Braaten BA, et al. (2005) Contact-dependent inhibition of growth in *Escherichia coli*. Science 309: 1245-1248.

25. Cowles KN, Goodrich-Blair H (2005) Expression and activity of a *Xenorhabdus nematophila* haemolysin required for full virulence towards *Manduca sexta* insects. Cell Microbiol 7: 209-219.

26. Brillard J, Duchaud E, Boemare N, Kunst F, Givaudan A (2002) The PhlA hemolysin from the entomopathogenic bacterium *Photorhabdus luminescens* belongs to the two-partner secretion family of hemolysins. J Bacteriol 184: 3871-3878.

27. Richards GR, Herbert EE, Park Y, Goodrich-Blair H (2008) *Xenorhabdus nematophila lrhA* is necessary for motility, lipase activity, toxin expression, and virulence in *Manduca sexta* insects. J Bacteriol 190: 4870-4879.

28. Galtier N, Gouy M, Gautier C (1996) SEAVIEW and PHYLO_WIN: two graphic tools for sequence alignment and molecular phylogeny. Comput Appl Biosci 12: 543-548.

29. Guindon S, Delsuc F, Dufayard JF, Gascuel O (2009) Estimating maximum likelihood phylogenies with PhyML. Methods Mol Biol 537: 113-137.

**Table 1.** Number of Tc toxin coding genes in *Xenorhabdus* and *Photorhabdus*

|  | **Subunit A** | **Subunit B** | **Subunit C** |
| --- | --- | --- | --- |
| ***X. nematophila*** | 7 | 3 | 3 |
| ***X. bovienii*** | 3 | 2 | 2 |
| ***P. luminescens*** | 11 | 3 | 7 |
| ***P. asymbiotica*** | 4 | 2 | 3 |

**Table 2**. Insecticidal gene clusters in *X. nematophila* and *X. bovienii*.

| **Locus** | **Gene** | **Left coordinate** | **Strand** | **Subunit type (class)** | **Amino Acid Length** | **Previously identified genes** | ***Photorhabdus* Ortholog** |
| --- | --- | --- | --- | --- | --- | --- | --- |
| ***X. nematophila*** | | | | | | | |
| Locus 1 | XNC1_2186 | 2106427 | - | B | 1516 | *-* | plu0961 *tcdB1* |
|  | XNC1_2187 | 2111038 | - | A (III) | 2417 | *-* | plu0965 *tcdA4*  (C-term only) |
|  | XNC1_2188 | 2119050 | - | C | 1030 | *-* | plu4167 *tccC1* |
| Locus 2 | XNC1_2333 | 2272819 | + | A (I) | 1021 | *-* | plu4169 *tccA* |
|  | XNC1_2334 | 2275956 | + | A (II) | 1541 | *-* | plu4168 *tccB1*  (C-term only) |
|  | XNC1_2335 | 2280669 | + | B | 1519 | *-* | plu0515 *tcaC* |
|  | XNC1_2336 | 2285221 | + | C | 969 | *-* | plu4488 *tccC7* |
| Locus 3 | XNC1_2560 | 2518637 | + | A (I) | 1156 | *xptE1* | plu2460 *tccA2* |
|  | XNC1_2561 | 2522114 | + | A (II) | 1391 | *xptD1^b^* | plu2459 *tccB2* |
| Locus 4^a^ | XNC1_2566 | 2531280 | + | A (III) | 2523 | *xptA1/sepA^b^* | plu0962 *tcdA1* |
|  | XNC1_2567 | 2538951 | - | C | 1016 | *xptB1* | plu4167 *tccC1* |
|  | XNC1_2568 | 2542055 | - | B | 1475 | *xptC1/sepB* | plu0515 *tcaC* |
|  | XNC1_2569 | 2546604 | - | A (III) | 2524 | *xptA2* | plu0962 *tcdA1* |
| Locus 5 | XNC1_3020 | 2995058 | + | A’ | 366 | *-* | *-* |
|  | XNC1_3021 | 2996104 | + | A’ | 345 | *-* | *-* |
|  | XNC1_3022 | 2997030 | + | A’ | 210 | *-* | *-* |
|  | XNC1_3023^c^ | 2997592 | + | A’ | 168 | *-* | *-* |
|  | XNC1_3024 | 2997959 | + | A’ | 197 | *-* | *-* |
| ***X. bovienii*** | | | | | | | |
| Locus 1 | XBJ1_0568 | 576005 | - | A (II) | 1368 | *-* | plu2459 *tccB2* |
|  | XBJ1_0569 | 580104 | - | A (I) | 1184 | *-* | plu2460 *tccA2* |
| Locus 2 | XBJ1_1572 | 1524551 | + | A (III) | 2517 | *-* | plu0962 *tcdA1* |
|  | XBJ1_1573 | 1532156 | + | B | 1481 | *-* | plu0961 *tcdB2/tcaC1* |
|  | XBJ1_1574 | 1536681 | + | C | 932 | *-* | plu0964 *tccC5* |
| Locus 3 | XBJ1_1934 | 1865759 | + | B | 1506 | *-* | *-* |
| Locus 4 | XBJ1_2397 | 2371437 | + | B' | 50 | *-* | *-* |
| Locus 5 | XBJ1_3085 | 3039594 | - | C | 901 | *-* | plu0964 *tccC5* |

**^a^** Locus identified previously

^b^ XptA1 was shown to be central for insect toxin activity . *xptD1* mutants have reduced virulence toward *Manduca sexta* larvae .

**^c^** Protein product was detected in *X. nematophila* supernatants.

**Table 3.** Putative invasin genes in the genomes of *Xenorhabdus nematophila* (XNC1) an *X. bovienii* (XBJ1).

| **Gene** | **Comments** | **Product** | **Cluster** |
| --- | --- | --- | --- |
| XNC1_0514 | Contains conserved domains (PRK10177: invasin domain and DUF823 domain) | putative invasin | II |
| XNC1_2538 | Contains conserved domains (PRK10177: invasin domain and BID_1 domain:Ig-like) | putative invasin (fragment) | I |
| XNC1_3566 | Contains conserved domains (PRK10177: invasin domain and five BID_1 domains:Ig-like) | putative invasin | III |
| XNC1_3690 | Contains conserved domains (PRK10177: invasin domain and two BID_1 domains:Ig-like) | putative invasin | III |
| XBJ1_0197 | Contains conserved domains (PRK10177: invasin domain and two BID_1 domains:Ig-like) | putative invasin | I |
| XBJ1_0377 | Contains conserved domains (PRK10177: invasin domain and DUF823 domain) | putative invasin | II |
| XBJ1_1098 | Contains conserved domains (PRK10177: invasin domain and DUF823 domain) | putative invasin | II |
| XBJ1_1104 | Contains conserved domains (PRK10177: invasin domain and DUF823 domain) | putative invasin | II |
| XBJ1_1508 | Contains conserved domains (PRK10177: invasin domain and five BID_1 domains:Ig-like) | putative invasin | III |
| XBJ1_2149 | Contains conserved domains (PRK10177: invasin domain; two Big_1 and four BID_1 domains: Ig-like) | putative invasin | III |
| XBJ1_2726 | Contains conserved domains (PRK10177: invasin domain) | putative invasin | I |
| XBJ1_3402 | Contains conserved domains (PRK10177: invasin domain and five BID_1 domains:Ig-like) | putative invasin | III |
| XBJ1_3796 | Contains conserved domains (PRK10177: invasin domain and DUF823 domain) | putative invasin | II |

**Table 4.**  Inventory of Tps-like proteins (TpsA/TpsB proteins) in the *X. bovienii*, *X. nematophila*, *P. asymbiotica,* and *P. luminescens* genomes.

| **Organism** | **Locus Tag** | **Length (AA)** | **Product** |
| --- | --- | --- | --- |
| *X. bovienii* | XBJ1_1977 (TpsA) | 642 | Putative TpsA-related protein (fragment) |
|  | XBJ1_1975 (TpsB) | 453 | TpsB protein |
|  | XBJ1_0258 (TpsA) | 1482 | Hemolysin (TpsA-related protein) |
|  | XBJ1_0259 (TpsB) | 556 | Hemolysin secretion protein (TpsB) |
| *X. nematophila* | XNC1_3688 (TpsA) | 906 | Putative TpsA-related protein (fragment) |
|  | XNC1_3689 (TpsB) | 565 | TpsB protein |
|  | XNC1_4556 (TpsA) | 1470 | hemolysin XhlA (TpsA) |
|  | XNC1_4555 (TpsB) | 557 | XhlB, hemolysin secretion protein (TpsB) |
|  | XNC1_3564 (TpsA) | 1554 | Putative TpsA-related protein |
|  | XNC1_3565 (TpsB) | 568 | TpsB protein |
| *P. asymbiotica* | PHA_0303 (TpsA) | 3029 | Putative TpsA-related protein |
|  | PHA_0302 (TpsB) | 91 | putative TpsB protein (fragment) |
|  | PHA_1972 (TpsA) | 2679 | TpsA-related protein |
|  | PHA_1973 (TpsB) | 91 | putative TpsB protein (fragment) |
|  | PHA_2333 (TpsA) | 2953 | TpsA-related protein |
|  | PHA_2334 (TpsB) | 91 | putative TpsB protein (fragment) |
|  | PHA_2590 (TpsA) | 1480 | Hemolysin PhlA (TpsA) |
|  | PHA_2589 (TpsB) | 526 | PhlB hemolysin secretion protein (TpsB) |
|  | PHA_4567 (TpsA) | 2989 | TpsA-related protein |
|  | PHA_4566 (TpsB) | 468 | TpsB protein |
|  | PHA_4934 (TpsA) | 2930 | TpsA-related protein |
|  | PHA_4935 (TpsB) | 559 | TpsB protein |
|  | PHA_3712 (TpsA) | 2952 | TpsA-related protein |
|  | PHA_4498 (TpsA) | 1753 | Putative TpsA-related protein |
| *P. luminescens* | plu0225 (TpsA) | 1738 | Putative TpsA-related protein |
|  | plu0226 (TpsB) | 559 | TpsB protein |
|  | plu0316 (TpsA) | 1480 | Hemolysin PhlA (TpsA) |
|  | plu0317 (TpsB) | 555 | PhlB hemolysin secretion protein (TpsB) |
|  | plu1149 (TpsA) | 2937 | TpsA-related protein |
|  | plu1150 (TpsB) | 554 | TpsB protein |
|  | plu1367 (TpsA) | 2961 | TpsA-related protein |
|  | plu1368 (TpsB) | 554 | TpsB protein |
|  | plu3718 (TpsA) | 3027 | TpsA-related protein |
|  | plu3719 (TpsB) | 554 | TpsB protein |
|  | plu3064 (TpsA) | 1695 | Putative TpsA-related protein |
|  | plu3065 (TpsB) | 559 | TpsB protein |
|  | plu3569 (TpsB) | 559 | TpsB protein |
|  | plu3577 (TpsA) | 928 | Putative TpsA-related protein |
|  | plu3594 (TpsA) | 2135 | Putative TpsA-related protein |
|  | plu2453 (TpsA) | 1687 | Putative TpsA-related protein |

**Figure 1. Tc toxins of *Xenorhabdus* spp.** Phylogenetic trees of *Photorhabdus* and *Xenorhabdus* Tc toxin A subunits (A) and N-terminal 750 aa and C-terminal 300 aa regions of C subunits (B). The A subunit phylogeny shows three distinct classes (I-III). Numbers at each node indicate bootstrap values (100 replicates). Asterisks indicate homologs for which ADP ribosylating activity has been demonstrated. Genomic regions of *Xenorhabdus* and select *Photorhabdus* tc loci are shown in (C) with color coding corresponding to the phylogenies shown in A and B.

**
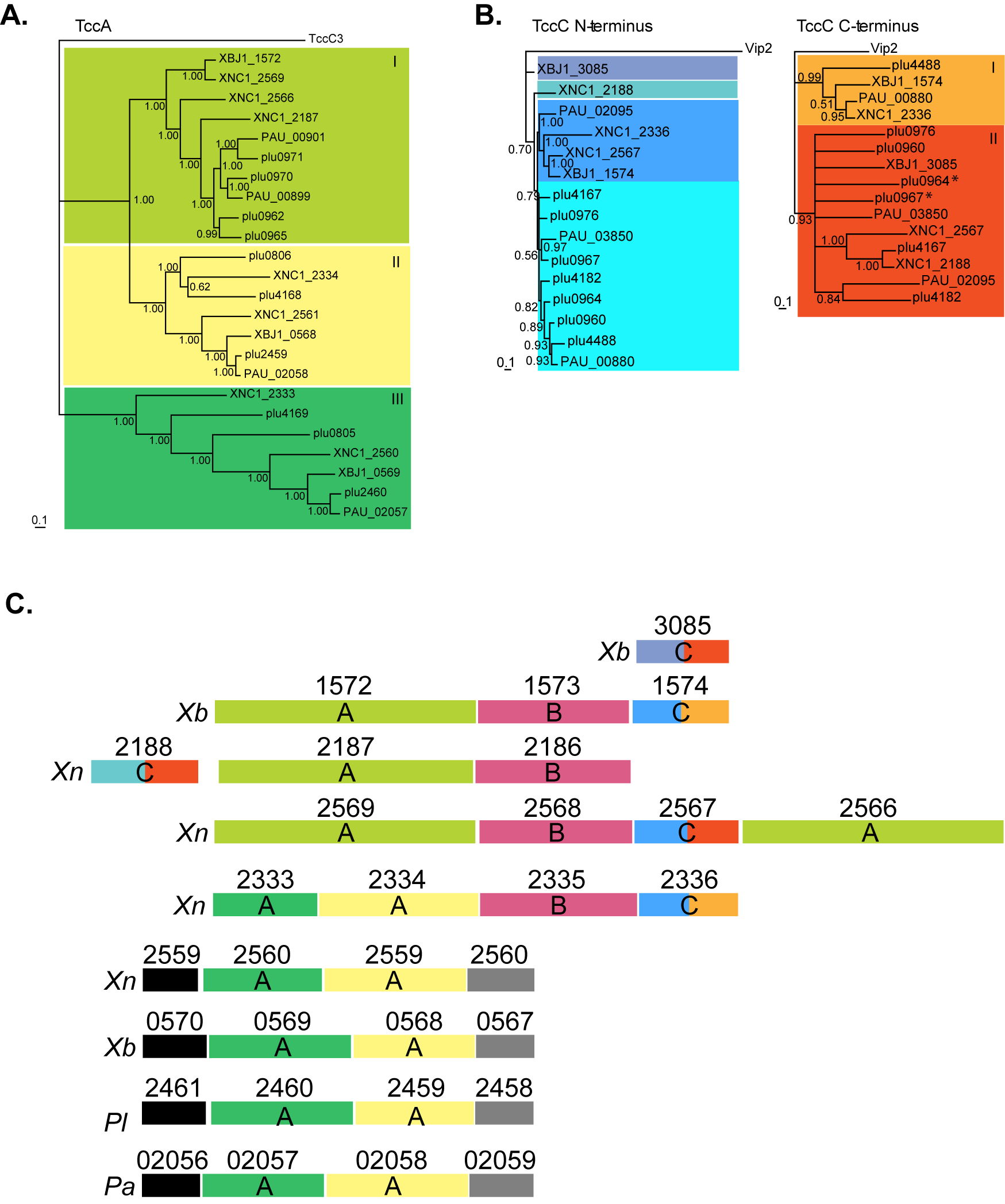
**

**Figure 2.** **Repartition of putative invasins of *Xenorhabdus* in a phylogenetic tree.** A phylogenetic tree showing the invasin proteins of *Xenorhabdus* cluster into three groups. Amino acid sequences were aligned using the CLUSTAL W program implemented in SEAVIEW . Using the SEAVIEW platform, phylogenetic trees were made using PhyML , a fast and accurate maximum likelihood heuristic method, under the LG substitution model, with 100 bootstrap replicates.

**Figure 3. Repartition of putative TpsA proteins of *Xenorhabdus* and *Photorhabdus*.** A phylogenetic tree showing TpsA proteins from *Xenorhabdus* cluster into three groups. The functional characterizations of subsets of TpsA proteins are marked in blue Amino acid sequences were aligned using the CLUSTAL W program implemented in SEAVIEW . Using the SEAVIEW platform, phylogenetic trees were made using PhyML , a fast and accurate maximum likelihood heuristic method, under the LG substitution model, with 100 bootstrap replicates.
