# Supplementary material for: The Entomopathogenic Bacterial Endosymbionts Xenorhabdus and Photorhabdus: Convergent Lifestyles from Divergent Genomes
Source: PLoS One. 2011 Nov 18;6(11):e27909. doi: 10.1371/journal.pone.0027909 (PMC3220699; doi:10.1371/journal.pone.0027909)
Supplement: Text S6 — Secondary Metabolites. (DOC) [file pone.0027909.s014.doc]

**Text S6: Secondary metabolites**

Edna Bode, Helge B. Bode, and Alexander O. Brachmann

Institut für Molekulare Biowissenschaften, Goethe Universität Frankfurt, Frankfurt am Main, Germany

E-mail: h.bode@bio.uni-frankfurt.de

Seven different structural classes of secondary metabolites have been isolated from different *Xenorhabdus* species indicating a rich repertoire of small molecules that may function in host interactions. Both *Xenorhabdus bovienii* and *Xenorhabdus nematophila* genomes encode a large number of different biosynthesis gene clusters responsible for the production of secondary metabolites (Table 1, below), and the overall percentage of the genome dedicated to secondary metabolism (*X. bovienii* 6.1%, *X. nematophila* 7.5%) is more than the *Streptomycetes* (*S. coelicolor* 4.5% ; *S. avermitilis* (6.6% ) but comparable to *Photorhabdus luminescens* TT01 (5.9% ). In *X. nematophila,* all clusters except one are located between 1.1 and 2.8 Mb in the genome (Figure 1, below), similar to the observed super-clustering of biosynthesis gene clusters in *Myxococcus xanthus* . No such super-clustering is present in *X. bovienii* (Figure 1, below) or *P. luminescens* TT01 .

A large biosynthesis gene cluster encoding three NRPSs that are predicted to produce a large lipopeptide with several lipophilic amino acids is located at similar locations in the genomes of *X. bovienii* and *X. nematophila*; the cluster size is 60 kb and 43 kb in *X. bovienii* and *X. nematophila*, respectively.

Nematophin , xenocoumacins 1-6 , xenematide, and xenortides have been isolated from *X. nematophila* ATCC 19061 (Bode, unpublished data), whereas two substituted indoles have been isolated from *X. bovienii* SS-2004 (Bode, unpublished data) (Figure 1, below). Additionally, the biosynthesis gene clusters for the production of xenocoumacins, xenematide, xenortides have all been identified in the genome of *X. nematophila* and the biosynthesis genes for the production of the indole derivatives in *X. bovienii* (Bode, unpublished): No biosynthesis gene cluster could be identified for the production of nematophin, which might be an NRPS-derived dipeptide, and putative clusters involved in its biosynthesis are currently being analyzed in detail. A biosynthesis gene cluster involved in the production of the isonitril compound rhabduscin is highly conserved among *Xenorhabdus* and *Photorhabdus* strains and has been identified in both *X. nematophila* *and X. bovienii* (Bode, unpublished).

One and two gene clusters involved in the production of iron-siderophores have been identified in the genomes of *X. nematophila* and *X. bovienii*, respectively. Whereas a highly similar gene cluster most likely involved in the production of a hydroxamate-like siderophore could be found in *X. nematophila* , *X. bovienii* and *P. luminescens* TT01, *X. bovienii* seems to be able to produce a second enterochelin-like siderophore (Figure 2, below) which shows no similarity to the NRPS-derived siderophore compound postulated from *P. luminescens* TT01. However, all three strains have been shown to produce siderophores by the usual CAS-agar test. Furthermore, disruption of *tonB* in *X. nematophila* led to a siderophore overproducer which accumulates a compound that can also be found in *X. bovienii,* as shown by HPLC-MS. This might indicate that *X. nematophila* and *X. bovienii* do actually produce the same hydroxamate siderophore. This siderophore is currently isolated for structure elucidation (Bode, unpublished data).

As already known from other Gram-negative bacteria (e.g. myxobacteria ), pure PKS gene clusters are rare and peptides derived from NRPS dominate. The genome of *P. luminescens* encodes a gene cluster for a polyketide derived from a type II PKS , which is currently the only example of a type II PKS encoding biosynthesis gene cluster from Gram-negative bacteria and which is not found in *P. asymbiotica* . Furthermore, biosynthesis gene cluster for the production of isopropylstilbenes and diketopiperazines has been identified in *P. luminescens* which are not present in both *Xenorhabdus* strains*.* In general a high number of NRPS clusters putatively involved in the biosynthesis of lipopeptides and peptides have been identified in all four genomes. Interestingly, amino acids with non-polar side chains are often the most abundant amino acids in these lipopeptides and would make these compounds even more hydrophobic, which might indicate a need for such compounds in the life cycle of *Xenorhabdus* and *Photorhabdus*. This is additionally supported by the finding that most compounds isolated from *Xenorhabdus* or *Photorhabdus* to date are very lipophilic .

Several of the NRPS-encoding gene clusters from *X. nematophila* and *X. bovienii* show no terminal thioesterase domain, as is usually present, but do have a condensation domain that might be involved in product release and/or cyclisation as postulated for cyclosporin formation in the fungus *Tolypocladium* *niveum* . Additionally, the specificities of several adenylation (A) domains could not be assigned using standard prediction tools. The reason for this might be that most A-domains found in the databases are derived from Gram-positive organisms (*Streptomyces*, *Bacillus*) and the specificities for the activation of not all but some amino acids might be different from *Xenorhabdus*. Similar results have been observed for *P. luminescens* TT01 but the number of non-assigned A-domains is much higher in *X. nematophila* and *X. bovienii*. A second possibility is that these clusters are non-functional and have been changed during evolution. However, this possibility begs the further question of why these clusters are maintained in the *Xenorhabdus* spp. genomes. Moreover, the finding that clusters with up to four identical A-domain specificities have been identified which could not be assigned to any known specificity in the databases makes an evolutionary loss of function unlikely.

**Methods**

Secondary metabolite gene clusters were identified by BLASTP searches of the annotated genomes using different adenylation (A), condensation (C), ketosynthase (KS), acyltransferase (AT), and thiolation (T) domains as bait. The functional annotation of the identified loci was performed using BLASTP and a program written by Jaques Ravel (http://www.tigr.org/jravel/nrps) for the prediction of polyketide synthase (PKS) and non-ribosomal synthetase (NRPS) domain structures. Adenylation domain specificities were predicted using the program NRPSpredictor . As the specificity of several A-domains could not be determined this way, several of these domains were exemplarily aligned with the phenylalanine-activating A domain of the gramicidin producing NRPS (GrsA) using ClustalW and identification of the conserved residues that form the specific binding pocket by comparison of the unknown sequences with GrsA (phe) .

**References**

1. Piel J (2004) Metabolites from symbiotic bacteria. Nat Prod Rep 21: 519-538.

2. Brachmann AO, Forst S, Furgani GM, Fodor A, Bode HB (2006) Xenofuranones A and B: phenylpyruvate dimers from *Xenorhabdus szentirmaii*. J Nat Prod 69: 1830-1832.

3. Lang G, Kalvelage T, Peters A, Wiese J, Imhoff JF (2008) Linear and cyclic peptides from the entomopathogenic bacterium *Xenorhabdus nematophilus*. J Nat Prod 71: 1074-1077.

4. Gualtieri M, Aumelas A, Thaler JO (2009) Identification of a new antimicrobial lysine-rich cyclolipopeptide family from *Xenorhabdus nematophila*. J Antibiot (Tokyo) 62: 295-302.

5. Fuchs SW, Proschak A, Jaskolla TW, Karas M, Bode HB (2011) Structure elucidation and biosynthesis of lysine-rich cyclic peptides in *Xenorhabdus nematophila*. Org Biomol Chem 9: 3130-3132.

6. Bode HB (2009) Entomopathogenic bacteria as a source of secondary metabolites. Curr Opin Chem Biol 13: 224-230.

7. Bentley SD, Chater KF, Cerdeno-Tarraga AM, Challis GL, Thomson NR, et al. (2002) Complete genome sequence of the model actinomycete *Streptomyces coelicolor* A3(2). Nature 417: 141-147.

8. Ikeda H, Ishikawa J, Hanamoto A, Shinose M, Kikuchi H, et al. (2003) Complete genome sequence and comparative analysis of the industrial microorganism *Streptomyces avermitilis*. Nat Biotechnol 21: 526-531.

9. Duchaud E, Rusniok C, Frangeul L, Buchrieser C, Givaudan A, et al. (2003) The genome sequence of the entomopathogenic bacterium *Photorhabdus luminescens*. Nat Biotechnol 21: 1307-1313.

10. Goldman BS, Nierman WC, Kaiser D, Slater SC, Durkin AS, et al. (2006) Evolution of sensory complexity recorded in a myxobacterial genome. Proc Natl Acad Sci U S A 103: 15200-15205.

11. Li J, Chen G, Webster JM (1997) Nematophin, a novel antimicrobial substance produced by *Xenorhabdus nematophilus* (Enterobactereaceae). Can J Microbiol 43: 770-773.

12. Reimer D, Luxenburger E, Brachmann AO, Bode HB (2009) A new type of pyrrolidine biosynthesis is involved in the late steps of xenocoumacin production in *Xenorhabdus nematophila*. Chembiochem 10: 1997-2001.

13. Thaler JO, Baghdiguian S, Boemare N (1995) Purification and characterization of xenorhabdicin, a phage tail-like bacteriocin, from the lysogenic strain F1 of *Xenorhabdus nematophilus*. Appl Environ Microbiol 61: 2049-2052.

14. Crawford JM, Kontnik R, Clardy J (2010) Regulating alternative lifestyles in entomopathogenic bacteria. Curr Biol 20: 69-74.

15. Martens EC, Russell FM, Goodrich-Blair H (2005) Analysis of *Xenorhabdus nematophila* metabolic mutants yields insight into stages of *Steinernema carpocapsae* nematode intestinal colonization. Mol Microbiol 51: 28-45.

16. Brachmann AO, Joyce SA, Jenke-Kodama H, Schwar G, Clarke DJ, et al. (2007) A type II polyketide synthase is responsible for anthraquinone biosynthesis in *Photorhabdus luminescens*. Chembiochem 8: 1721-1728.

17. Wilkinson P, Waterfield NR, Crossman L, Corton C, Sanchez-Contreras M, et al. (2009) Comparative genomics of the emerging human pathogen *Photorhabdus asymbiotica* with the insect pathogen *Photorhabdus luminescens*. BMC Genomics 10: 302.

18. Joyce SA, Brachmann AO, Glazer I, Lango L, Schwar G, et al. (2008) Bacterial biosynthesis of a multipotent stilbene. Angew Chem Int Ed Engl 47: 1942-1945.

19. Lautru S, Gondry M, Genet R, Pernodet JL (2002) The albonoursin gene Cluster of S noursei biosynthesis of diketopiperazine metabolites independent of nonribosomal peptide synthetases. Chem Biol 9: 1355-1364.

20. Piel J (2009) Metabolites from symbiotic bacteria. Nat Prod Rep 26: 338-362.

21. Weber G, Leitner E (1994) Disruption of the cyclosporin synthetase gene of *Tolypocladium niveum*. Curr Genet 26: 461-467.

22. Altschul SF, Madden TL, Schaffer AA, Zhang J, Zhang Z, et al. (1997) Gapped BLAST and PSI-BLAST: a new generation of protein database search programs. Nucleic Acids Res 25: 3389-3402.

23. Challis GL, Ravel J, Townsend CA (2000) Predictive, structure-based model of amino acid recognition by nonribosomal peptide synthetase adenylation domains. Chem Biol 7: 211-224.

24. Rausch C, Weber T, Kohlbacher O, Wohlleben W, Huson DH (2005) Specificity prediction of adenylation domains in nonribosomal peptide synthetases (NRPS) using transductive support vector machines (TSVMs). Nucleic Acids Res 33: 5799-5808.

25. Larkin MA, Blackshields G, Brown NP, Chenna R, McGettigan PA, et al. (2007) Clustal W and Clustal X version 2.0. Bioinformatics 23: 2947-2948.

26. Stachelhaus T, Mootz HD, Marahiel MA (1999) The specificity-conferring code of adenylation domains in nonribosomal peptide synthetases. Chem Biol 6: 493-505.

**Table 1.** Number of putative compounds encoded by biosynthesis gene clusters in *Xenorhabdus bovienii*, *X. nematophila*, and *Photorhabdus luminescens*.

| **Biosynthetic Cluster Type** | ***X. bovienii*** | ***X. nematophila*** | ***P. luminescens*** |
| --- | --- | --- | --- |
| peptides | 2 | 8 | 8 |
| lipopeptides | 6 | 5 | 2 |
| Polyketide/peptide hybrids | 1 | 3 | 2 |
| polyketides | - | - | 1 |
| NRPS-siderophores | 1(2) | - | 1 |
| hydroxamate-siderophores | 1 | 1 | 1 |
| Others/unknown ^a^ | 3 | - | 6 |
| Total number of clusters | 15 | 17 | 21 |
| Percent of genome (%) | 6.1 | 7.5 | 5.9 |

^a^clusters that do not fit in any of the other categories.

**Figure 1.** Known compounds isolated from *X. nematophila* and *X. bovienii*. Structures were determined after preparative isolation using NMR, MS and/or high-resolution MS.

**Figure 2.** Selection of predicted compounds from *X. nematophila* and *X. bovienii*.
