# Supplementary material for: The Entomopathogenic Bacterial Endosymbionts Xenorhabdus and Photorhabdus: Convergent Lifestyles from Divergent Genomes
Source: PLoS One. 2011 Nov 18;6(11):e27909. doi: 10.1371/journal.pone.0027909 (PMC3220699; doi:10.1371/journal.pone.0027909)
Supplement: Text S7 — A proteomic analysis of the Xenorhabdus nematophila supernatant. (DOC) [file pone.0027909.s015.doc]

**Text S7: A proteomic analysis of the *Xenorhabdus* *nematophila* supernatant**

Nancy Leimgruber^1^, Dongjin Park^2^, Bosong Xiang^1^, Steven Forst^2^, Barry S. Goldman^1^, and Heidi Goodrich-Blair^3^

^1^Monsanto Company, St. Louis, Missouri, United States of America

^2^Department of Biological Sciences, University of Wisconsin-Milwaukee, Milwaukee, Wisconsin, United States of America

^3^Department of Bacteriology, University of Wisconsin-Madison, Madison, Wisconsin, United States of America

Email: hgblair@bact.wisc.edu

To begin to determine which proteins might be secreted by *X. nematophila*, and therefore interact directly with either insect or nematode host cells, we analyzed the protein composition of supernatant of *X. nematophila* cultures grown for 16 h in LB broth at 30˚C. Among the 322 visible spots, the 100 most intense were picked, and of those, 67 were identified with high confidence using MALDI-TOF (Table 1, below).

Of the proteins identified, only one, MrxA, is bioinformatically predicted to be a secreted product. Twenty-one were predicted to be membrane or periplasmic, indicating the sample may contain outer membrane vesicles (OMV). *X. nematophila* OMV are toxic toward *Helicoverpa armigera* insects, have affiliated chitinase activity, and are associated with several proteins: the pilin subunit MrxA, the outer membrane protein OpnA, and the outer membrane protein OpnP . Each of these proteins was detected in the *X. nematophila* supernatant tested here, as was a predicted outer membrane chitinase (XNC1_2559, previously designated *chi-2* by ) (Table 3, below). Two toxin homologs were detected in the extracellular sample; with one of these (XNC1_1142) being 98% similar to the *P. luminescens* TT01 gene plu4092, which encodes the PirB toxin ; the other (XNC1_3023) encodes a fragment of a Tc-like toxin A subunits with similarity to *tcdA4* (AAO17209.1) from *P. luminescens* (Table 1, below) . Given the common appearance of "cytoplasmic" proteins in the extracellular supernatant protein profiles of many bacteria, including *X. nematophila* and *P. luminescens* (Table 1, below), it is possible that bacteria actively transport such proteins to outer membrane vesicles to serve specific functions in those environments.

*X. nematophila* OMV have been associated with chitinase activity, and *chi-2,* a predicted chitinase, was found in the supernatant . The *chi-2* protein is encoded on a previously reported pathogenicity island that also encodes another predicted chitinase XN_2562 (Chi-1) (with no PSortB predicted sub-cellular location) and several Toxin complex (Tc) subunits (Text S5) . *X. bovienii* encodes three predicted chitinases: XB_1652, XB_0570 (predicted by PSortB to be an outer membrane protein), and XB_0567. As in *X. nematophila,* two of these chitinases flank A-subunit-encoding *tc* genes. However, these four genes are not located on the previously identified island (Figure 2, main text) . ClustalW alignment indicates that *X. nematophila* Chi-1 and *X. bovienii* XB_0567 are homologs (58.7% identity), but that Chi-2 is an outlier (<46% identity) (data not shown). These chitinases may synergize Tc toxin activity to aid in virulence . Alternatively, chitinases may serve a mutualistic role with the nematode host. The sugar-beet root maggot utilizes *Serratia* chitinases to aid in pupal emergence . Similarly, *Xenorhabdus* spp. may aid in the development of their nematode hosts. Taken together, our data and those of Khandelwal and Banerjee-Bhatnagar (2003) suggest that the entomotoxic outer membrane vesicles of *X. nematophila* carry Chi-2 and that this activity may play a role in virulence toward insects.

Among the additional proteins in the *X. nematophila* was a phage tail tube protein and a homolog of an A subunit of the Tc toxin complex. Expression of the *P. luminescens tcdA* and *tcdB* genes in *E. coli* conferred expression of a phage like particle in the toxic fractions . Bacteriophage and their remnants are often linked to “lysogenic conversion genes,” which are not involved in the phage life cycle but instead can increase the adaptive fitness of the bacterial host. Thus, in pathogens, lysogenic conversion genes can encode factors that increase virulence and resistance to host-defenses . In *P. luminescens,* so-called “Photorhabdus virulence cassettes” (PVC), 13-15 ORFs repeated multiple times in the genome, are regions of phage-related DNA with associated genes encoding predicted or known virulence determinants . Unexpectedly, proteins identified as extracellular included 24 that either have no predicted sub-cellular location or are predicted to be cytoplasmic proteins. The presence of predicted cytoplasmic proteins in the culture supernatant may be indicative of cell death during sample processing. However, there is growing evidence that glycolytic and cytoplasmic enzymes can be surface localized in both bacteria and eukaryotic cells. For example, *P. luminescens* and *Listeria monocytogenes* both have predicted cytoplasmic proteins in the extracellular milieu, and many of these are homologs of proteins found in *X. nematophila* supernatant (Table 1, below). Furthermore, peptidyl-prolyl cis-trans isomerase, manganese superoxide dismutase, glutathione S transferase, and phosphoglycerate mutase are secreted from non-small cell lung cancer cell lines . The functions of such surface or secreted enzymes are largely unknown. One of the better-characterized examples is glyceraldehyde-3-phosphate dehydrogenase (GAPDH), which was thought to be exclusively a cytoplasmic protein but can also be present on the surface of cells. Surface-localized GAPDH of *Paracoccidioides brasiliensis* can bind plasmin, fibronectin, and laminin , while that of *Streptococcus pyogenes* cleaves human complement C5a . Furthermore, GAPDH is present on the surface of macrophages, where it serves as a transferrin receptor . Our findings therefore suggest that *X. nematophila* GAPDH, and perhaps other unexpected extracellular proteins, may help mediate host interactions. For example, during insect infection, *X. nematophila* colonizes the connective tissue , and surface expressed GAPDH may help mediate adherence to this region.

**Methods**

A 10 ml culture of *Xenorhabdus nematophila* was grown in Luria Bertani broth at 30^o^C for 18 hr. The bacteria were pelleted by centrifugation at 6,000xg (15 min) and the culture supernatant was filtered through a 0.22 μm Millipore filter at 4^o^C. The filtrate was concentrated to 400 μl by centrifugation at 5000 x g at 4^o^C (Amicon Ultra, Millipore). The concentrated sample was centrifuged at 353,000 x g at 4^o^C for 14 min (TL100 Beckman) to remove insoluble and particulate material and the resulting supernatant was concentrated and processed to remove potentially interfering substances by precipitation using the EMD-Calbiochem ProteoExtract Kit. Protein was precipitated from a 200 uL sample aliquot. The resulting washed, precipitated protein was solubilized in 1.08 mL of 2-D gel lysis/rehydration cocktail (7M urea, 2M thiourea, 16% isopropanol, 0.50% CHAPS, 0.25% Triton X-100, 0.25% SB 3-10, 0.35% pH 3-10 carrier ampholyte mixture, 100 mM DTT, 5.0% glycerol, 0.001 % bromophenol blue and 1x Roche Complete Protease Inhibitor Cocktail-EDTA free by vortexing vigorously.

Total protein was then quantified using the Cytoskeleton Advance 01 protein assay prior to 2-D Gel Electrophoresis.

For 2-D Gel Electrophoresis, Proteins were then fractionated based on charge in the first dimension and by molecular weight in the second dimension. Isoelectric focusing was performed using 18 cm, pH 3–10, non-linear, immobilized pH gradient (IPG) gel strips (GE Healthcare). Each IPG strip was rehydrated using 360 uL of sample solution containing 280 ug total protein for 22 hours under 2.0 mL of light mineral oil at room temperature. Following rehydration and brief blotting to remove excess mineral oil, each strip was placed on electrode wicks wetted with deionized water and covered with 2.0 mL of light mineral oil. Isoelectric focusing was performed at 20 °C in a BioRad Protean IEF Cell using the following program: rapid ramp to 50 volts for 2 hours, rapid ramp to 250 volts for 2 hours, rapid ramp to 500 volts for 2 hours, linear ramp to 8,000 volts over 10 hours, holding at 8,000 volts until 63,500 volt-hours was reached.

After focusing, each IPG strip was equilibrated in 4.0 mL of 62.5 mM Tris, 2.3% (w/v) SDS, 20 mM dithiothreitol, 0.001% (w/v) bromophenol blue, pH 8.8 for 8 minutes. The equilibration solution was removed and replaced with 4.0 mL of 62.5 mM Tris, 2.3% (w/v) SDS, 50 mM iodoacetamade, 0.001% (w/v) bromophenol blue, pH 6.8, for 8 minutes. Strips were embedded on the surface of three 10-18% 20 X 20 cm Tris-HCl gels using 1% melted agarose in equilibration buffer. Electrophoresis was carried out overnight at 2 watts/gel constant power until the bromophenol blue tracking dye migrated to the bottom edge of the gels. Gels were fixed in ~250 mL of 40% (v/v) methanol, 7% (v/v) acetic acid for two hours at room temperature on an orbital rotator. Each gel was washed twice with deionized water for 10 minutes then stained in 350 mL of colloidal Coomassie blue G-250 protein stain for 48 hours at room temperature on an orbital rotator.

Coomassie stained gels were then destained with three changes of de-ionized water until the gel background was clear then scanned using a Bio-Rad GS-800 calibrated densitometer. Image analyses and identification of proteins of interest were performed using PDQuest image analysis software, v8.01. An analysis set for identification was generated from the 100 most abundant protein spots of the 322 total proteins detected. These were cut into 96 well trays for enzymatic digestion using a BioRad ExQuest Spotcutter.

**References**

1. Khandelwal P, Banerjee-Bhatnagar N (2003) Insecticidal activity associated with outer membrane vesicles of *Xenorhabdus nematophilus*. Applied and Environmental Microbiology 69: 2032-2037.

2. Sergeant M, Baxter L, Jarrett P, Shaw E, Ousley M, et al. (2006) Identification, typing, and insecticidal activity of *Xenorhabdus* isolates from entomopathogenic nematodes in United Kingdom soil and characterization of the *xpt* toxin loci. Applied and Environmental Microbiology 72: 5895-5907.

3. Waterfield N, Kamita SG, Hammock BD, ffrench-Constant R (2005) The *Photorhabdus* Pir toxins are similar to a developmentally regulated insect protein but show no juvenile hormone esterase activity. FEMS Microbiology Letters 245: 47-52.

4. ffrench-Constant R, Waterfield N (2006) An ABC guide to the bacterial toxin complexes. Advances in Applied Microbiology 58: 169-183.

5. Pardo-Lopez L, Munoz-Garay C, Porta H, Rodriguez-Almazan C, Soberon M, et al. (2009) Strategies to improve the insecticidal activity of Cry toxins from Bacillus thuringiensis. Peptides 30: 589-595.

6. Wilkinson P, Waterfield NR, Crossman L, Corton C, Sanchez-Contreras M, et al. (2009) Comparative genomics of the emerging human pathogen Photorhabdus asymbiotica with the insect pathogen Photorhabdus luminescens. BMC Genomics 10: 302.

7. Iverson KL, Bromel MC, Anderson AW, Freeman TP (1984) Bacterial symbionts in the sugar beet root maggot *Tetanops myopaeformis* (von Roder). Applied and Environmental Microbiology 47: 22-27.

8. Waterfield N, Bowen DJ, Fetherston JD, Perry RD, ffrench-Constant RH (2001) The toxin complex genes of *Photorhabdus*: a growing gene family. Trends in Microbiology 9: 185-191.

9. Brüssow H, Canchay C, Hardt W-D (2004) Phages and the evolution of bacterial pathogens: from genomic rearrangements to lysogenic conversion. Microbiology and Molecular Biology Reviews 68: 560-602.

10. Yang G, Dowling AJ, Gerike U, ffrench-Constant RH, Waterfield NR (2006) *Photorhabdus* virulence cassettes confer injectable insecticidal activity against the wax moth. Journal of Bacteriology 188: 2254-2261.

11. Turlin E, Pascal G, Rousselle J-C, Lenormand P, Ngo S, et al. (2006) Proteome analysis of the phenotypic variation process in *Photorhabdus luminescens*. Proteomics.

12. Schaumburg J, Diekmann O, Hagendorff P, Bergmann S, Rohde M, et al. (2004) The cell wall subproteome of *Listeria monocytogenes*. Proteomics 4: 2991-3006.

13. Huang LJ, Chen SX, Huang Y, Luo WJ, Jiang HH, et al. (2006) Proteomics-based identification of secreted protein dihydrodiol dehydrogenase as a novel serum markers of non-small cell lung cancer. Lung Cancer 54: 87-94.

14. Barbosa MS, Bao SN, Andreotti PF, de Faria FP, Felipe MS, et al. (2006) Glyceraldehyde-3-phosphate dehydrogenase of *Paracoccidioides brasiliensis* is a cell surface protein involved in fungal adhesion to extracellular matrix proteins and interactions with cells. Infection and Immunity 74: 382-389.

15. Terao Y, Yamaguchi M, Hamada S, Kawabata S (2006) Multifunctional glyceraldehyde-3-phosphate dehydrogenase of *Sterptococcus pyogenes* is essential for evasion from neutrophils. The Journal of Biological Chemistry 281.

16. Raje CI, Kumar S, Harle A, Nanda JS, Raje M (2007) The macrophage cell surface glyceraldehyde-3-phosphate dehydrogenase is a novel transferrin receptor. Journal of Biological Chemistry 282: 3252-3261.

17. Sicard M, Brugirard-Ricaud K, Pages S, Lanois A, Boemare NE, et al. (2004) Stages of infection during the tripartite interaction between *Xenorhabdus nematophila*, its nematode vector, and insect hosts. Applied and Environmental Microbiology 70: 6473-6480.

18. Nandakumar MP, Cheung A, Marten MR (2006) Proteomic Analysis of Extracellular Proteins from Escherichia coli W3110. Journal of Proteome Research 5: 1155-1161.

19. Målen H, Berven FS, Fladmark KE, Wiker HG (2007) Comprehensive analysis of exported proteins from Mycobacterium tuberculosis H37Rv. Proteomics 7: 1702-1718.

20. Turlin E, Pascal G, Rousselle JC, Lenormand P, Ngo S, et al. (2006) Proteome analysis of the phenotypic variation process in Photorhabdus luminescens. Proteomics 6: 2705-2725.

21. Seul KJ, Park SH, Ryu CM, Lee YH, Ghim SY (2007) Proteome analysis of Paenibacillus polymyxa E681 affected by barley. J Microbiol Biotechnol 17: 934-944.

22. Mattinen L, Nissinen R, Riipi T, Kalkkinen N, Pirhonen M (2007) Host-extract induced changes in the secretome of the plant pathogenic bacterium Pectobacterium atrosepticum. Proteomics 7: 3527-3537.

23. Schaumburg J, Diekmann O, Hagendorff P, Bergmann S, Rohde M, et al. (2004) The cell wall subproteome of Listeria monocytogenes. Proteomics 4: 2991-3006.

24. Trost M, Wehmhöner D, Kärst U, Dieterich G, Wehland J, et al. (2005) Comparative proteome analysis of secretory proteins from pathogenic and nonpathogenic Listeria species. Proteomics 5: 1544-1557.

25. Watt SA, Wilke A, Patschkowski T, Niehaus K (2005) Comprehensive analysis of the extracellular proteins from Xanthomonas campestris pv. campestris B100. Proteomics 5: 153-167.

**Table 1.** *X. nematophila* proteins detected in culture supernatants

| ***Locus*** | **Gene Start** | **Gene^a^** | **Predicted function** | **PSortB/ SignalP^b^** | ***X. bovienii* ORF** | **Other features^c^** |
| --- | --- | --- | --- | --- | --- | --- |
| XNC1_0025 | 28662 | *atpD* | F_0_F_1_ ATP synthase, beta subunit | CP | 22 | Pl, MT, Ec* |
| XNC1_0029 | 33108 | *atpF* | F_0_F_1_ ATP synthase, beta subunit | CP | 26 | Pl |
| XNC1_0053 | 58071 | *sodA* | manganese superoxide dismutase | NP | 51 | Pl, Lm, Xc, Mt |
| XNC1_0381 | 316087 | *trxA* | thiol-disulfide isomerase and thioredoxins | CP | 4182 |  |
| XNC1_0481 | 414255 | *ppa* | inorganic pyrophosphatase | CP | 344 | Pl, Xc |
| XNC1_0509 | 440013 | *pnp* | polyribonucleotide nucleotidyltransferase | CP | 372 |  |
| XNC1_0616 | 526896 | *dcp* | Zn-dependent oligopeptidases | PP/CM/SP | 93 |  |
| XNC1_0643 | 555717 | *glpQ* | glycerophosphoryl diester phosphodiesterase | PP/SP | 541 |  |
| XNC1_0744 | 638542 | *oppA_3_* | ABC oligopeptide transport | PP/SP | 1694 | Pa, Lm |
| XNC1_1082 | 961331 | *lpdA* | pyruvate dehydrogenase | CP | 3453 | Lm |
| XNC1_1142 | 1019735 | *pirB* | N-terminal endotoxin, insect toxin | NP | none |  |
| XNC1_1207 | 1075474 | - | phage tail tube protein FII | CP | 1368 | Pl |
| XNC1_1215 | 1082789 | *eco* | Serine protease inhibitor ecotin | PP/SP | 1360 |  |
| XNC1_1342 | 1204340 | *gltI* | ABC-type amino acid transport/signal transduction | PP/SP | 3202 |  |
| XNC1_1373 | 1232292 | *ccdB* | Similarity to cytotoxic protein CcdB | NP | none |  |
| XNC1_1411 | 1291508 | *sucC* | succinyl coA synthetase beta subunit | CP | 1055 | Pl |
| XNC1_1421 | 1301596 | *pal* | Outer membrane protein (OmpA family); peptidoglycan associated lipoprotein | OM/SP | 1043 |  |
| XNC1_1425 | 1305730 | *gpmA* | Phosphoglycerate mutase 1 | NP | 1038 | Lm, Xc |
| XNC1_1586 | 1475341 | *ompF* | Outer membrane protein porin (OpnA)/OmpN | OM/SP | 794 | OMV; Ec |
| XNC1_1718 | 1621399 | *flgK* | Flagellar hook-associated protein | OM | 1961 | Pp, Pa, Ec |
| XNC1_1877 | 1801605 | *sodB* | iron superoxide dismutase | NP | 2494 |  |
| XNC1_1880 | 1803847 | *gloA* | lactoylglutathione lyase | NP | 2491 |  |
| XNC1_2243 | 2184530 | - | glutamate decarboxylase family | CP | 2352 |  |
| XNC1_2249 | 2190494 | - | None; putative exported protein | NC/SP | none | SP |
| XNC1_2275 | 2220532 | *pcaH* | Protocatechuate dioxygenase, putative exported | NC/SP | 1940 | SP |
| XNC1_2453 | 2379995 | - | none | CP | none |  |
| XNC1_2478 | 2429711 | *oppA_2_* | ABC-type oligopeptide transport system | PP/SP | 2423 |  |
| XNC1_2479 | 2431482 | *oppA_1_* | ABC-type oligopeptide transport system | PP/SP | 2424 |  |
| XNC1_2510 | 2457677 | *gapA* | glyceraldehyde-3-phosphate dehydrogenase | CP | 2452 | Lm, Xc, Mt, Pa |
| XNC1_2559 | 2516901 | *chi-2* | Chitinase; virulence determinant | OM | 1652 | OMV; Lm* |
| XNC1_2654 | 2613624 | *dapE* | acetylornithine deacetylase | NP | 2595 |  |
| XNC1_2702 | 2662884 | - | none | NP | 2376 |  |
| XNC1_2800 | 2800004 | *can* | Carbonic anhydrase | NC | none | SP |
| XNC1_2873 | 2881172 | - | none | CP | 1283 |  |
| XNC1_2922 | 2921953 | - | none | CP | none |  |
| XNC1_2923 | 2922365 | - | none | NP | none |  |
| XNC1_2963 | 2949217 | *terD* | tellurium resistance protein | NP | 1784 |  |
| XNC1_2976 | 2963218 | - | none | CP | 1122 | HTH |
| XNC1_3023 | 2997592 | *tcdA4* | Toxin complex | NP | 1572 |  |
| XNC1_3197 | 3143396 | *fadL* | Long chain fatty acid transport protein | OM/SP | 2994 |  |
| XNC1_3211 | 3156943 | *cysK* | cysteine synthase | NP | 3122 | Pl, Lm, Mt |
| XNC1_3216 | 3160499 | *crr* | PTS IIA | CP | 3127 | Lm* |
| XNC1_3292 | 3219396 | *glyA* | serine/glycine hydroxymethyltransferase | CP | 3033 | Pl, Lm |
| XNC1_3305 | 3229749 | *pepB* | leucyl aminopeptidase | NP | 3021 |  |
| XNC1_3338 | 3261979 | *fabH* | 3-oxoacyl-acyl carrier protein synthase | CP | 3626 | Mt (fabG4) |
| XNC1_3604 | 3489864 | *groES* | GroES | CP | 3571 | Pl, Lm |
| XNC1_3607 | 3491013 | *groEL* | GroEL | CP | 3570 | Pl, Lm, Xc, Pa |
| XNC1_3774 | 3630982 | *opnP* | Outer membrane protein, ompC/OpnP | OM/SP | 1704 | OMV; Pl |
| XNC1_3803 | 3661001 | *mrxA* | MrxA, pilin subunit | XC/SP | 3538 | OMV |
| XNC1_3804 | 3662269 | *deoD* | purine nucleoside phosphorylase | CP | 3539 |  |
| XNC1_3889 | 3747052 | *udp* | uridine phosphorylase | CP | 502 | Pl |
| XNC1_4173 | 4030469 | *sspA* | glutathione S-transferase | CP | 3846 |  |
| XNC1_4186 | 4039508 | *yrbC* | ABC-type transport system involved in resistance | NC /SP | 3833 |  |
| XNC1_4237 | 4081686 | *glyA* | glycine/serine hydroxymethyltransferase | CP | 3033 | Pl Lm |
| XNC1_4257 | 4096871 | *pepA* | leucyl aminopeptidase | NP | 447 | Xc, Mt |
| XNC1_4325 | 4155276 | *dhnA/ lsrF* | fructose 1,6, bisphosphate aldolase | NP | 4104 | Lm, Xc, Mt, Pa |
| XNC1_4330 | 4158926 | *dsbA* | thiol-disulfide isomerase | PP/SP | 4097 |  |
| XNC1_4334 | 4164342 | *ppiA* | peptidyl-prolyl cis-trans isomerase | PP/SP | 4092 | Xc, Mt, Pa |
| XNC1_4371 | 4195830 | *fusA* | translation elongation factor | CP | 4063 | Lm |
| XNC1_4494 | 4296673 | *dppA* | ABC type dipeptide transport system | PP/SP | 246 |  |
| XNC1_4596 | 4390686 | *pckA* | PEP carboxykinase | PP | 71 | Mt |

^a^ Gene designations are from *X. nematophila* published sequences (underlined) or the gene name of the closest homolog.

^b^Determined using PSortB: http://www.psort.org/psortb/. SP indicates ORF was identified with ≥ 0.85 probability of having a signal peptide at amino acid ≥ 15. Other predictions include extracellular (XC), periplasmic (PP), non-cytoplasmic (NC), no prediction (NP), and cytoplasmic (CP).

^c^The homologous protein was identified in *E. coli* K12 (W3110) , *Mycobacterium tuberculosis*  ,*Photorhabdus luminescens* , *P. polymyxa P. atrosepticum*  *Listeria monocytogenes* , or *Xanthomonas campestris* supernatants respectively. *X. campestris* superoxide dismutase is *sodM. E. coli* has the a subunit of ATP synthase. Trost study shows chitinase in *L. monocytogenes,* but this is not a homolog of *X. nematophila* chitinases. L.m. showed PtsH (Hpr) while *X. nematophila* has PTS IIA. The presence of a signal peptide (SP) or helix-turn-helix (HTH) domain is listed as predicted by PSortB. OMV indicates the protein or a related activity was detected associated with outer membrane vesicles.
