# Supplementary material for: The Entomopathogenic Bacterial Endosymbionts Xenorhabdus and Photorhabdus: Convergent Lifestyles from Divergent Genomes
Source: PLoS One. 2011 Nov 18;6(11):e27909. doi: 10.1371/journal.pone.0027909 (PMC3220699; doi:10.1371/journal.pone.0027909)
Supplement: Text S8 — Xenorhabdus multi-locus sequence analysis. (DOC) [file pone.0027909.s016.doc]

**Text S8: *Xenorhabdus* Multi-locus Sequence Analysis**

Pradeep R. Marri^1^ and Renyi Liu^1,2^

^1^Department of Ecology and Evolutionary Biology, University of Arizona, Tucson, Arizona, United States of America

^2^ Current address: Department of Botany and Plant Sciences, University of California, Riverside, CA 92521

Email: renyi.liu@ucr.edu

Although the 16S rRNA phylogeny places *Xenorhabdus nematophila* closer to *Photorhabdus luminescens* than to *X.* *bovienii* (Figure 3, main text and Figure 1, below), a tree based on 154 concatenated protein sequences indicates that the two *Xenorhabdus* congeners are actually closer to each other (Figure 1A, below). To evaluate whether the relationship of *X. nematophila* and *P. luminescens* is blurred by extensive gene exchanges, we tested whether or not the alignment of any of the 154 single-copy genes used in the concatenation supports an alternative topology. Applying the SH test , we statistically tested each of the three possible topologies for the three species, with *Yersinia pestis* as an outgroup. Only one (*rplA*) of the 154 genes significantly rejects the consensus topology (Figure 1B, below) and favors the 16S rRNA tree (Figure 3, main text). Recombination among the 16S rRNA genes of different bacterial species is rare but has been reported , and the alternative topology supported by 16S rRNA genes appears to be caused by a recombination event involving >50 nucleotides near the 5’ end of the *X. bovienii* 16S gene with that of a distantly related species.

**Methods**

To establish the phylogenetic position of *Xenorhabdus*, we selected one published genome sequence for several genera within the family Enterobacteriaceae. Analyses were based on a total of 13 genomes (Figure 1, below), including the two *Xenorhabdus* genomes reported in the present study, using ***Vibrio cholerae* as the outgroup.**

**For each species, 16S rRNA sequences were retrieved from GenBank, and an alignment was constructed in MUSCLE applying the default parameters. Gblocks was used to remove any poorly conserved regions, and phylogenetic analyses were performed in PhyML .**

**To build the phylogeny based on protein sequences, we selected 154 genes from the set 203 conserved single copy genes in *Gammaproteobacteria* identified by Lerat et al. . The alignment for each gene was constructed in MUSCLE and then concatenated to form a larger alignment, which was used to obtain a consensus tree. As above, poorly conserved regions were removed with GBlocks, and PhyML used to build the phylogenetic tree. To determine if the alignment of each individual gene significantly favors a tree topology that differs from the tree built from the concatenated protein alignment, we applied the SH-test implemented in TreePuzzle .**

**References**

1. Shimodaira H, Hasegawa M (1999) Multiple Comparisons of Log-Likelihoods with Applications to Phylogenetic Inference. Molecular Biology and Evolution 16: 1114.

2. Yap WH, Zhang Z, Wang Y (1999) Distinct types of rRNA operons exist in the genome of the actinomycete Thermomonospora chromogena and evidence for horizontal transfer of an entire rRNA operon. J Bacteriol 181: 5201-5209.

3. Badger JH, Eisen JA, Ward NL (2005) Genomic analysis of Hyphomonas neptunium contradicts 16S rRNA gene-based phylogenetic analysis: implications for the taxonomy of the orders 'Rhodobacterales' and Caulobacterales. Int J Syst Evol Microbiol 55: 1021-1026.

4. Edgar RC (2004) MUSCLE: multiple sequence alignment with high accuracy and high throughput. Nucleic Acids Res 32: 1792-1797.

5. Castresana J (2000) Selection of conserved blocks from multiple alignments for their use in phylogenetic analysis. Mol Biol Evol 17: 540-552.

6. Guindon S, Gascuel O (2003) A simple, fast, and accurate algorithm to estimate large phylogenies by maximum likelihood. Syst Biol 52: 696-704.

7. Lerat E, Daubin V, Moran NA (2003) From gene trees to organismal phylogeny in prokaryotes: the case of the gamma-Proteobacteria. PLoS Biol 1: E19.

8. Schmidt HA, Strimmer K, Vingron M, von Haeseler A (2002) TREE-PUZZLE: maximum likelihood phylogenetic analysis using quartets and parallel computing. Bioinformatics 18: 502-504.

**Figure 1. The phylogenetic relationship *Xenorhabdus* within the *Enterobacteriaceae.*** (A) 16S rRNA phylogenetic tree for *Enterobacteriaceae*. (B) Phylogenetic tree based on the concatenated protein alignments of 154 single-copy genes that are present in all genomes considered. For both trees, ***Vibrio cholerae*** was included as an outgroup. Bootstrap values are reported out of 100 replicates.

**
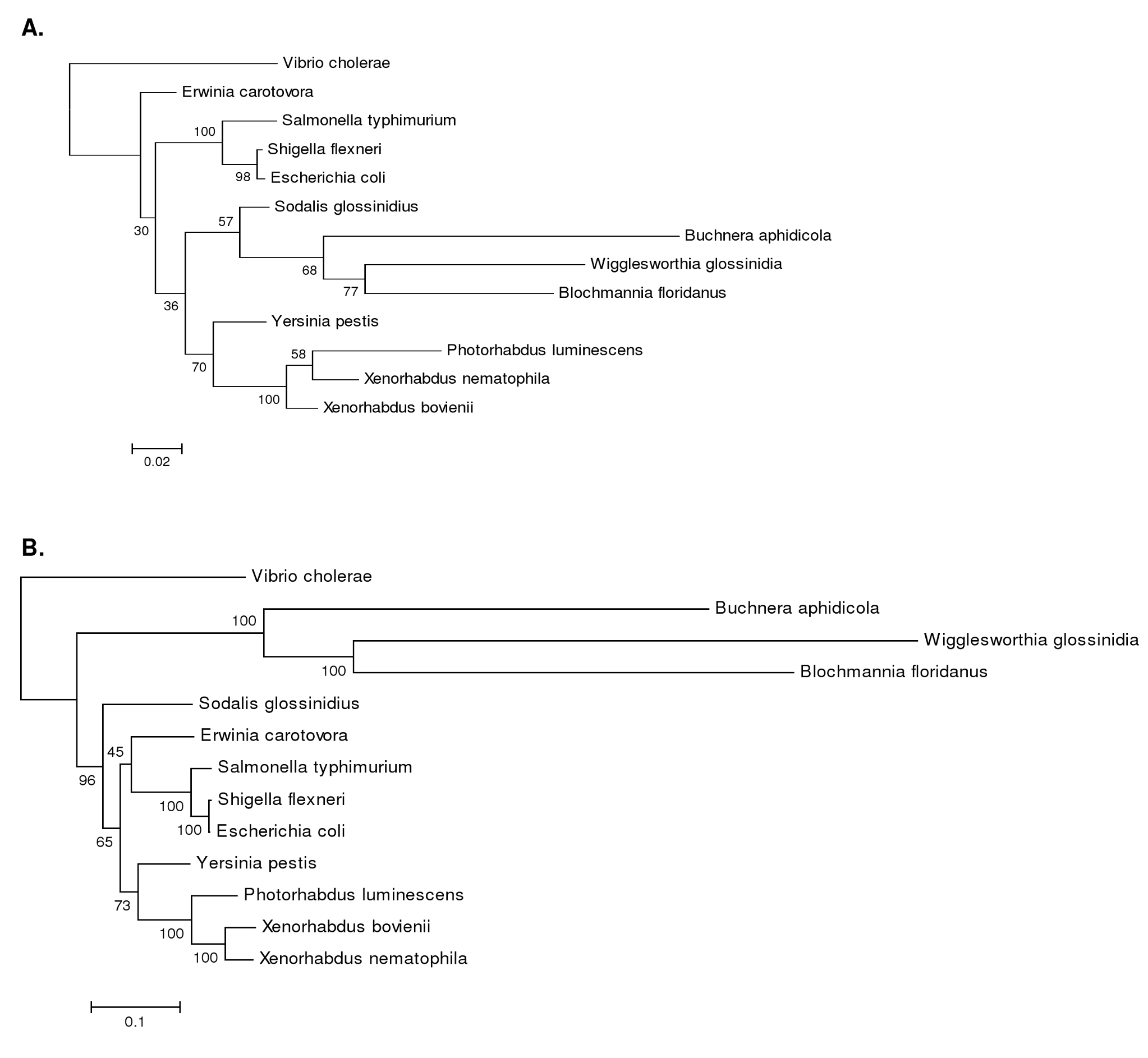
**
